# Pendulum-like hemilability in a Ti-based frustrated Lewis Trio†

**DOI:** 10.1039/d3sc06789k

**Published:** 2024-03-08

**Authors:** Errikos Kounalis, Dylan van Tongeren, Stanislav Melnikov, Martin Lutz, Daniël L. J. Broere

**Affiliations:** a Organic Chemistry and Catalysis, Institute for Sustainable and Circular Chemistry, Faculty of Science, Utrecht University, Universiteitsweg 99 3584 CG Utrecht The Netherlands d.l.j.broere@uu.nl; b Structural Biochemistry Bijvoet Centre for Biomolecular Research, Faculty of Science, Utrecht University, Universiteitsweg 99 3584 CG Utrecht The Netherlands

## Abstract

We describe the first experimental example of a theoretically predicted Frustrated Lewis Trio (FLT). A tetradentate PNNP ligand is used to stabilise a highly electrophilic [TiCl_3_]^+^ fragment in a way that results in two equally long and frustrated Ti–P bonds. A combined experimental and computational approach revealed a distinct role of each Lewis basic phosphine in the heterolytic activation of chemical bonds. This dual functionality is characterised by a pendulum-like hemilability, where one of the phosphines acts as a nucleophile while the other serves as a hemilabile ligand that dynamically tunes the Ti–P distance as a function of the required electron density at the Ti centre.

## Abstract 2

We describe the first experimental example of a theoretically predicted Frustrated Lewis Trio (FLT), which displays pendulum-like hemilability in heterolytic bond activation processes.

## Graphical abstract

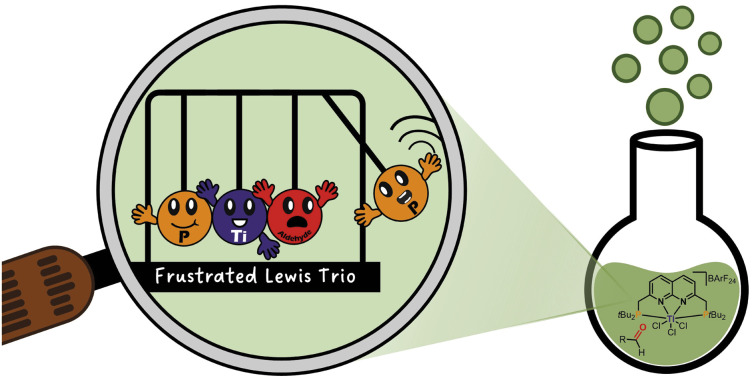

## Introduction

**Fig. 1:**
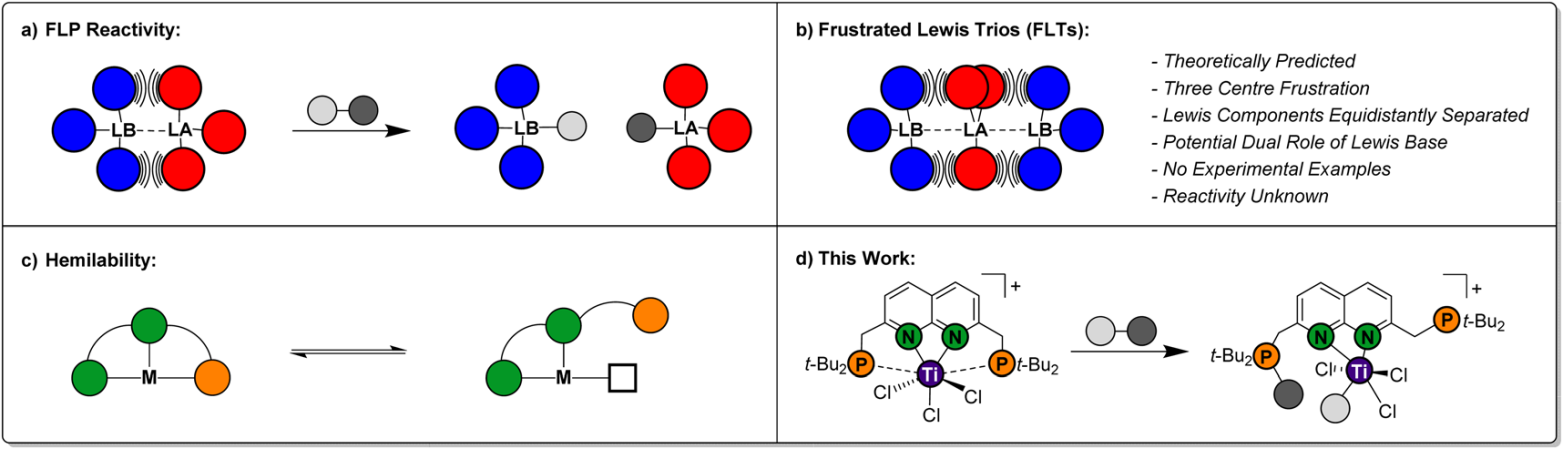
Schematic representations of: (a) heterolytic bond activation using a Frustrated Lewis Pair (FLP); (b) the Frustrated Lewis Trio (FLT) concept; (c) ligand hemilability and (d) the combination of these three concepts demonstrated in this work.

Over the past two decades, Frustrated Lewis Pairs (FLPs) have successfully been employed in a variety of stoichiometric and catalytic transformations involving the heterolytic activation of bonds in small molecules.^1–5^ This reactivity is driven by the effective quenching of the ‘frustration’ in the Lewis acid/base pair, which is caused by ring strain and/or steric hindrance preventing the formation of the classical Lewis adducts in an intra-^6^ or intermolecular^7^ manner (Fig. 1a). Seminal work mostly involved combinations of phosphines as the Lewis base and boranes as the Lewis acid. As the field evolved, a variety of FLP combinations have been developed based on combinations of different elements, such as *ansa*-aminoboranes^8^ and geminal P/Al FLPs.^9^ The scope of stoichiometric reactivity of FLPs with small molecules is vast. Selected examples include the archetypal reversible H_2_ activation,^10^ the binding of CO_2_ and CO^11^ and the ring-opening of epoxides.^12^

The FLP paradigm can be extended to transition metals (TM-FLPs), where either of the Lewis pair partners, or both, can be TM-based.^13–15^ An extensively studied class of TM-FLPs is the family of cationic group 4 metallocene phosphinoaryloxide complexes developed by the group of Wass.^16^ Similar to main group FLPs, the formation of the Lewis adduct between the group 4 metal centre and the pendant phosphine in these complexes is hampered. Remarkably, this motif extended the scope of FLP-type reactivity to the activation of the C–O bond in non-cyclic ethers and even the activation of C–F and C–Cl bonds in alkyl halides.^17^ Since these initial reports, a variety of cationic group 4 TM-FLPs have been reported by the groups of Erker, Le Gendre, Stephan, Beckhaus and others.^18–28^ Most of these TM-FLPs share a common element: solid-state structures that display long bond distances between P and a cationic metal centre, which is supported by electron-donating auxiliary ligands (often Cp-derived).

Ligand hemilability is a concept that is widely employed by both metalloenzymes and coordination chemists.^29^ It involves one of the donors of a polydentate ligand being able to reversibly dissociate from the metal centre (Fig. 1c), thereby stabilising reactive intermediates prior to binding of an exogenous substrate, or after product dissociation. Through this, hemilabile ligands can provide flatter potential energy surfaces to give higher reaction rates. To the best of our knowledge, the use of pendant, hemilabile donors has not been described for TM-FLP chemistry.^30^ We reason that this is inherent to their design, which typically entails a robust complex featuring a single ‘frustrated’ site. Although phosphinoamide-based multi-site FLPs have been reported by the groups of Stephan and Zhu, in these systems each frustrated acid–base pair reacted as an individual FLP.^24,31^

Conceptually, less rigid systems wherein the ‘frustration’ is shared over two chemical bonds, could enable one of the Lewis bases to engage in heterolytic bond activation, while the other Lewis base functions as a hemilabile donor. The possibility to generate a system that displays a frustration expressed over three atoms was computationally predicted by Echeverría, who coined them as Frustrated Lewis Trios (FLTs).^32^ In these systems, a Lewis acid is located in between two Lewis bases, which are equidistantly separated to prevent forming classic Lewis acid/base pairs. The three-centre bonding in these FLTs, was calculated to be strong, highly directional while displaying base-acid-base angles between 170°–175° (Fig. 1b). Moreover, Echeverría found hints towards their existence in the crystal lattice of reported group 13 compounds. Despite the elegant simplicity of the concept, to the best of our knowledge experimental FLTs are unknown. Molecular designs that generate isolable FLTs, can enable investigations towards the behaviour of this motif, wherein the Lewis bases could play a beneficial dual role in heterolytic bond activation.

While exploring the chemistry of the *^t^*^-Bu^PNNP ‘expanded pincer’ ligand^33^ with early metals, we serendipitously synthesised the first isolated example of a theoretically predicted Frustrated Lewis Trio (Fig. 1d). Herein, we detail its synthesis, electronic structure and demonstrate the heterolytic activation of chemical bonds along the FLT vector. Finally, we show how this system displays pendulum-like hemilability between the dual-function phosphines, demonstrating the promise the FLT motif holds for the heterolytic activation of chemical bonds.

## Results and discussion

**Fig. 2:**
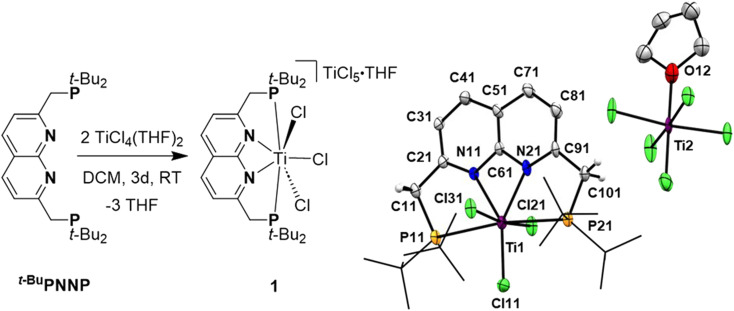
Synthesis of [*^t^*^-Bu^PNNPTiCl_3_][TiCl_5_(THF)] (1) by a reaction of the *^t^*^-Bu^PNNP ligand with two equiv. of TiCl_4_(THF)_2_ (left). On the right the displacement ellipsoid plot of 1 at 50% probability. Most hydrogen atoms, co-crystallised dichloromethane molecules and minor disorder components are omitted, and *t*-Bu groups on P are depicted as wireframe for clarity.

Reacting *^t^*^-Bu^PNNP with 2 equiv. of TiCl_4_(THF)_2_ in DCM at ambient temperature for 3 days led to a green reaction mixture. The ^1^H- and ^31^P{^1^H}-NMR spectra of the reaction mixture showed approx. 80% conversion to a new *C*_2v_-symmetric species. The chemical shifts of the ^31^P nuclei are significantly shifted downfield compared to the free ligand (*δ* = 77.4 ppm *vs.* 35.8 ppm in DCM), indicating coordination to Ti. Storing the reaction mixture for 2 h at −40 °C afforded green needle-shaped crystals suitable for single crystal X-ray diffraction analysis. The solid state structure (Fig. 2) revealed a [TiCl_3_]^+^ fragment stabilised by the PNNP ligand, and a THF-solvated TiCl_5_ anion. Similar auto-halide abstraction has been reported upon treating a bisiminopyridine–TiCl_4_ complex with an additional two equiv. of TiCl_4_, resulting in a ligated [TiCl_3_]^+^ core with a [Ti_2_Cl_9_]^−^ counterion.^34^ The striking feature of the structure we obtained is that the cationic fragment is not only bound to the N-donors of the ligand, but also to the P-donors, albeit at remarkably large Ti–P distances compared to the average of Ti–P distances reported in the literature (2.904(4) Å and 2.910(4) Å *vs.* 2.591 Å).^35^ Moreover, they are also longer than for the Ti–P FLP systems reported by the group of Wass (2.785(2) Å).^16^ Combined with the P–Ti–P angle of 171.6(2)°, this complex displays the key metric parameters of a FLT. Intrigued by this and the distinct structural dissimilarity with known transition metal-based FLPs, we set out to investigate the reactivity of the [*^t^*^-Bu^PNNPTiCl_3_]^+^ fragment.

**Fig. 3:**
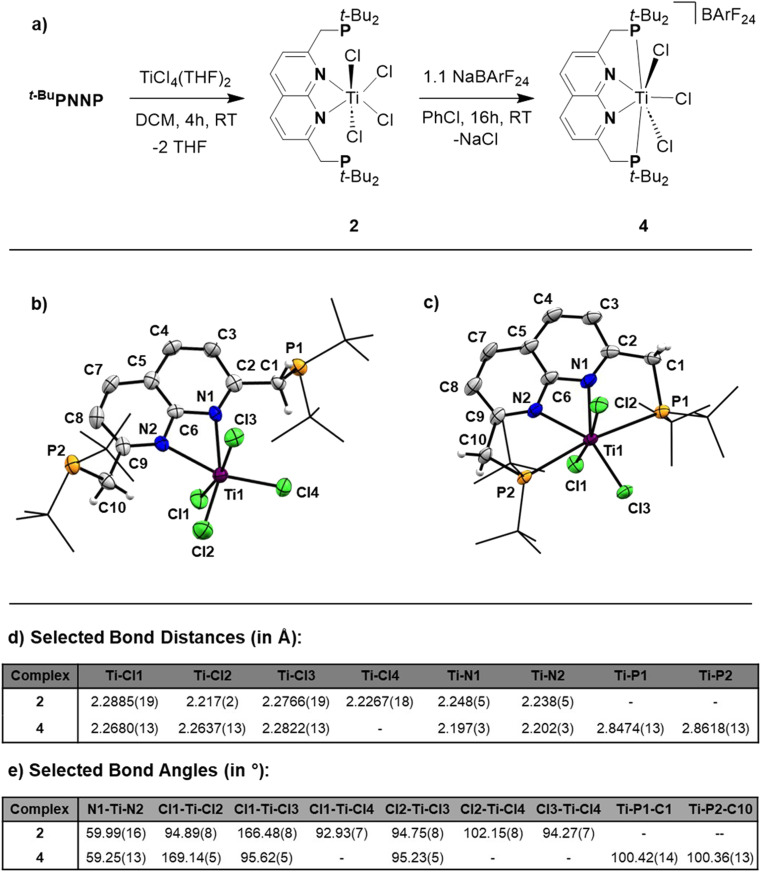
(a) Synthesis of 2 by complexation of the *^t^*^-Bu^PNNP ligand to TiCl_4_(THF)_2_. To generate the FLT motif a chloride is abstracted from 2 using NaBArF_24_ resulting in the formation of 4. (b) Displacement ellipsoid plot of 2 at 50% probability. Most hydrogen atoms, co-crystallised solvent molecules are omitted, and *t*-Bu groups on P are depicted as wireframe for clarity. (c) Displacement ellipsoid plot of 4 at 50% probability. Most hydrogen atoms and the BArF_24_ anion are omitted and *t*-Bu groups on P are depicted as wireframe for clarity. (d) Selected bond distances found in the solid-state structures of 2 and 4, denoted in Å. (e) Selected bond angles found in the solid-state structures of 2 and 4, denoted in °.

The poor solubility of [*^t^*^-Bu^PNNPTiCl_3_][TiCl_5_(THF)] and the possibility of the titanate anion engaging in side reactions prompted us to explore synthetic routes towards [*^t^*^-Bu^PNNPTiCl_3_]^+^ with a different non-coordinating anion. We hypothesised that synthesising the *^t^*^-Bu^PNNP adduct of TiCl_4_ followed by halide abstraction with NaBArF_24_ would be the most viable strategy and as such we first set out to synthesise the TiCl_4_-ligand adduct. Reacting *^t^*^-Bu^PNNP with one equiv. of TiCl_4_(THF)_2_ in CH_2_Cl_2_ at ambient temperature (Fig. 3a) resulted in the formation of complex 2, which was isolated as a dark red/brown powder in 82% yield. The ^1^H-, ^13^C{^1^H}- and ^31^P{^1^H}-NMR spectra show the expected number of resonances for a *C*_2v_-symmetric species. The ^31^P{^1^H}-NMR spectrum shows a sharp singlet at *δ* = 37.4 ppm, which is at a similar chemical shift as the free ligand (*δ* = 35.8 ppm in CD_2_Cl_2_), indicating that the pendant phosphines are not bound to the Ti centre. This is consistent with the solid-state structure of single crystals grown from a 1 : 4 benzene/pentane solution at −40 °C (Fig. 3b).

The overall geometry around the Ti centre in 2 is octahedral, with the N donors occupying proximal positions on the equatorial plane. The fact that the phosphines remain unbound in 2 is attributed to the coordinative saturation around the Ti centre. In line with this observation, the addition of 2 equiv. of AuCl·SMe_2_ to a solution of 2 in CH_2_Cl_2_ at ambient temperature resulted in the formation of heterotrinuclear 3, which was isolated as a yellow powder in 88% yield. The full structural characterisation of 3 is detailed in the ESI, section 1.4.†

To generate the [TiCl_3_]^+^ core and establish the P–Ti–P motif, a dark orange PhCl solution of 2 was treated with 1.1 equiv. NaBArF_24_ to abstract a chloride ligand (Fig. 3a). Upon addition of NaBArF_24_, the dark orange solution turned dark green and from this mixture 4 was isolated as dark-coloured needles in 72% yield. The ^1^H-, ^13^C{^1^H}- and ^31^P{^1^H}-NMR spectra show retention of the *C*_2v_-symmetry on the NMR timescale. Interestingly, the ^31^P{^1^H}-NMR spectrum in C_6_D_6_ at 298 K displays a singlet at *δ* = 76.1 ppm, which is ∼30 ppm downfield with respect to 2 (in C_6_D_6_), indicative of P–Ti bonding. The *t*-Bu and methylene resonances in ^1^H-NMR appear as overlapping doublets of doublets. These resonances appear as singlets in the ^31^P decoupled ^1^H-NMR spectrum showing that the multiplicity is due to virtual coupling or ^2^*J*_H,P_ and ^4^*J*_H,P_ coupling (see Fig. S23†). Combined with the observed *C*_2v_-symmetry, which is maintained even at 193 K, this observation shows that in solution both phosphines are bound to Ti.

Single crystals of 4 suitable for single crystal X-ray diffraction analysis were obtained from a saturated benzene solution (Fig. 3c). The solid-state structure confirmed the presence a [*^t^*^-Bu^PNNPTiCl_3_]^+^ fragment similar to that in 1, albeit with some minor differences in metric parameters (Fig. 3d and e). 4 exhibits a distorted pentagonal bipyramidal geometry around the Ti centre with two of the chloride ligands occupying the apical positions. The distortion is evident through the tilting of the least squares plane of [TiCl_3_]^+^ core compared to the least squares plane of the naphthyridine, with the angle between the two planes being 77.29(10)° (see Fig. S33a†). This goes paired with the twisting of the phosphines out of the naphthyridine plane (torsion angles of N1–C2–C1–P1 = 24.7(5)° and N2–C9–C10–P2 = 20.8(5)°) to keep the C9–C10–P2 and C2–C1–P1 angles at expected values for sp^3^ hybridised C-atoms. Similar to 1, the most remarkable feature of the solid-state structure are the unusually long Ti–P bonds of 4 (2.8474(13) Å and 2.8618(13) Å). We propose that these long bond distances are mainly due to the steric clashing of the chloride anions with the *t*-Bu substituents on the phosphines, which prevent effective stabilisation of the electrophilic [TiCl_3_]^+^ core (see Fig. S33b†). The aforementioned steric clashing being the main reason for the long Ti–P distance was also investigated computationally (see ESI, section 2.8†). Correcting the Ti–P, Ti–N and Ti–Cl bond lengths for their van der Waals radii employing the penetration index method further confirmed the remarkably long nature of the Ti–P bonds (see ESI, section S1.6†).^36^

The unusual way *^t^*^-Bu^PNNP binds the [TiCl_3_]^+^ core with both phosphines led us to investigate if a FLP-type Ti–P complex could be synthesised with only one phosphine donor and how this would affect reactivity. Using the *^t^*^-Bu^PNN^Me^ ligand we found that one phosphine cannot sufficiently stabilise the electrophilic [TiCl_3_]^+^ core, leading to side reactions with the non-coordinating anion (see ESI, sections 1.7–1.10†). These findings showcase the reactive nature of the electrophilic [TiCl_3_]^+^ fragment, which requires stabilisation by both phosphines to generate a FLT motif.

### Computational insights into the nature of the P–Ti–P motif

To obtain insights into the nature of the Ti–P interactions, we studied the cation of 4 computationally. The optimised gas phase geometry of 4^+^ (see ESI, section 2.5†) is in good agreement with the metric parameters of the solid-state structure (see Fig. S70†). Inspection of the frontier orbitals revealed bonding interactions with large coefficients along the P–Ti–P vector for the HOMO and HOMO-1 orbitals (see Fig. S71†). Both the LUMO and LUMO + 1 show large coefficients on Ti with antibonding interactions with the chloride-based orbitals.

**Fig. 4:**
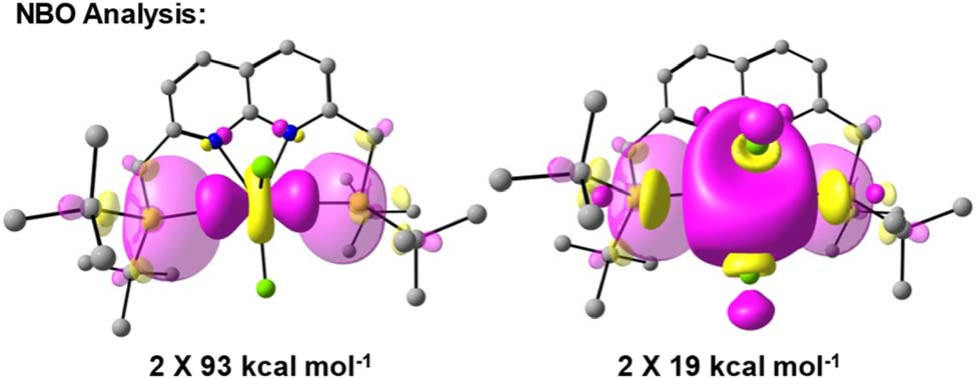
Overlap of the donor (translucent) and acceptor (opaque) NBOs found for 4^+^ with the respective delocalisation energies obtained from second order perturbation analysis. Both interactions were found in the analysis to be equivalent per Ti–P vector.

Using Natural Bond Order (NBO) analysis we investigated the electronic structure of 4^+^, which provides a more intuitive investigation of the bonding interactions compared to the highly delocalised Kohn–Sham orbitals described above. The NLMO/NPA bond order found for the Ti–P bonds was 0.4, slightly larger than the bond order found for the Ti–N bonds (0.2) and smaller than the bond orders found for the Ti–Cl bonds (0.6). Second order perturbation analysis allowed us to gauge the extent to which the P-donors interact with the Ti centre. The donor NBOs on both phosphines have a mixed s–p character (56% *vs.* 44% respectively) and donate into two acceptor NBOs on the Ti centre. The largest delocalisation energy of ∼93 kcal mol^−1^ was found towards an acceptor NBO on Ti of mainly 
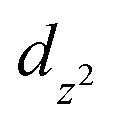
 character (97% d, 3% s, Fig. 4, left), which was significantly larger than the delocalisation energy of ∼19 kcal mol^−1^ found towards an acceptor NBO on Ti of mostly s character (96% s, 4% d, Fig. 4, right). Both these interactions were found to be equivalent for each P–Ti fragment. These computational insights combined show that an energetically appreciable bonding interaction can be found along the P–Ti–P motif, despite the steric encumbrance around the P and Ti atoms. These findings are in agreement with the abovementioned theoretical work on the FLT motif.^32^

To put these numbers in perspective we ran the same calculations on a known cationic titanocene-phosphinoaryloxide FLP reported by the group of Wass.^16^ Despite the different coordination environments around Ti in these two compounds, the electronic situation between Ti and P was found to be quite comparable (see ESI, sections 2.6 and 2.7†). These calculations show that the dissimilarity in the coordination environment around Ti such the presence of electron-withdrawing or -donating ligands on Ti is in sharp contrast with the observed similitude of electronic trends of the Ti–P motif. The main difference between the systems is that each Ti–P bond in the cation of 4 are weaker than the Ti–P bond found in the titanocene-based system. Despite the weaker bonding, the [TiCl_3_]^+^ core can still be sufficiently stabilised due to the presence of two such bonds.

### Reactivity studies

Having obtained theoretical insights into the FLT motif, we shifted our focus to explore the reactivity of 4. Exposing a solution of 4 in benzene to 1 bar of H_2_ resulted in an immediate colour change. This reaction gave an intractable mixture that was NMR-silent. We propose that H_2_ gets activated over the Ti–P bond, followed by homolytic cleavage of the Ti–H bond, as has been observed for Ti/P-based FLPs.^37^ Despite multiple attempts we were not able to isolate or characterise the formed species. Exposing a benzene solution of 4 to an atmosphere of CO_2_ also results in a rapid reaction concomitant with formation of a yellow, insoluble precipitate that prohibited fruitful analysis of the reaction products.

**Fig. 5:**
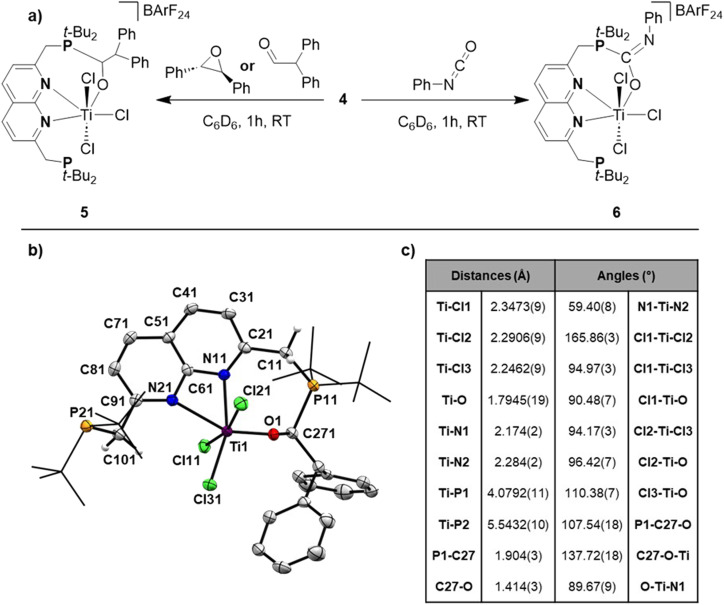
(a) Synthesis of 5 and 6 when reacting 4 with *trans*-stilbene oxide or diphenylacetaldehyde and phenyl isocyanate, respectively. (b) Displacement ellipsoid plot of 5 at 50% probability. Most hydrogen atoms, co-crystallised toluene molecules and the BArF_24_ anion are omitted, and *t*-Bu groups on P are depicted as wireframe for clarity. (c) Selected distances (in Å) and bond angles (in °) found in the solid-state structure of 5.

Inspired by earlier work on the FLP-type ring-opening of epoxides,^12^*trans*-stilbene oxide was added to a benzene solution of 4, resulting in a gradual colour change from green to red/orange. ^1^H- and ^31^P{^1^H}-NMR analysis of the mixture showed the formation of a new non-*C*_2v_ symmetric species (5) next to unreacted 4. The ^31^P{^1^H}-NMR spectrum showed two equally integrating resonances appearing at *δ* = 65.9 and 41.5 ppm. The latter lies in the region where the unbound phosphine resonance of 2 is found, whereas the former lies in between the chemical shift of the unbound phosphine and the phosphine bound to titanium. The ^1^H-NMR spectrum of this mixture gave rise to 5 new, equally integrating doublets of doublets in the region between *δ* = 3–4.5 ppm, which appeared as doublets in the ^1^H{^31^P} spectrum. This shows that the symmetry along both mirror planes of the naphthyridine is lost in 5, giving rise to 4 magnetically inequivalent methylene protons. The fifth resonance at *δ* = 4.48 ppm that displayed *J*_H,P_ coupling is magnetically coupled to a resonance at *δ* = 7.63 ppm. Although 5 could be reproducibly synthesised, isolation as a pure compound was frustrated by its instability that resulted in the formation of an intractable mixture within hours at room temperature. Despite these difficulties, storing a concentrated toluene solution of the product from the reaction of 4 with *trans*-stilbene oxide allowed us to obtain single crystals of 5 that were suitable for X-ray diffraction (Fig. 5b).

To our surprise we did not observe the expected ring-opened epoxide adduct but instead observed the FLP-type adduct of 4 with the carbonyl moiety of diphenylacetaldehyde (5, Fig. 5), which is consistent with the aforementioned NMR data. The geometry around Ti in 5 is distorted octahedral with the Ti–Cl_(ax)_ bonds being elongated compared to 2, 3, and 4. The C–O bond was found to be 1.414(3) Å, which is consistent with a single bond C–O character and shows the full extent of activation of the double bond of the aldehyde to a single bond.^38^

In the literature such a Lewis acid promoted rearrangement of epoxides to carbonyl compounds is known as the Meinwald rearrangement.^39^ We propose that in our case coordination of the epoxide to the Ti centre takes place, most likely after dissociation of one of the phosphines. After coordination, ring-opening can take place towards a zwitterionic fragment, followed by a phenyl shift and release of diphenylacetaldehyde. Monitoring this reaction at lower temperatures using VT-NMR spectroscopy showed no conversion of either 4 or the epoxide at appreciable rates below 25 °C. Moreover, at this temperature conversion to 5 was observed without any observable intermediates (see Fig. S59–S60†). We hypothesise that the [TiCl_3_]^+^-promoted Meinwald rearrangement is rapid and that the formed aldehyde gets activated over one of the Ti–P bonds. To corroborate that the diphenylacetaldehyde adduct we observed in the solid state matched the non-symmetric compound we observed in solution, we reacted 4 directly with diphenylacetaldehyde as depicted in Fig. 5.^40–42^ This also resulted in the formation of complex 5 based on NMR analysis (see Fig. S62–S63†), confirming the speciation in the solution matched that observed in the solid-state.

When exposing a benzene solution of 4 to phenyl isocyanate we observed a similar colour change as observed for the reactions of 4 with the epoxide and the aldehyde. ^1^H- and ^31^P{^1^H}-NMR analysis of the mixture revealed the formation of a new species (6) next to unreacted 4. The ^1^H-NMR spectrum of this mixture gave rise to 2 new, overlapping doublets (^2^*J*_H,P_ = 12.6 and 4.7 Hz) at the methylene region (*δ* = 3.40 ppm), which appeared as overlapping singlets in the ^1^H{^31^P} spectrum. Similarly, two new resonances were observed at *δ* = 1.05 and 0.88 ppm (^3^*J*_H,P_ = 11.8 and 16.7 Hz, respectively) for the *t-*Bu substituents on the phosphines which appeared as singlets in the ^1^H{^31^P} spectrum (see Fig. S65†). The observed number of resonances for the methylene and *t*-Bu protons are consistent with *C*_s_ symmetry, where only the symmetry of the mirror plane along the naphthyridine motif is retained. Resonances belonging to unreacted phenyl isocyanate obfuscated the chemical shift region *δ* = 7.0–6.5 ppm, preventing us from observing if an isocyanate-derived fragment is present in 6. To this end, we resorted to DOSY-NMR, using the differences in hydrodynamic radii (see Fig. S66†). Gratifyingly, the DOSY spectrum revealed phenyl isocyanate-derived resonances at a similar diffusion coefficient as 6. The ^31^P{^1^H} spectrum showed two equally integrating resonances at *δ* = 46.3 and 43.1 ppm, with the more downfield resonance being at a similar chemical shift as the reported phosphonium resonance of the C=O adduct of phenyl isocyanate to a geminal FLP (*t*-Bu_2_PCH_2_BPh_2_).^43^ The stability issues precluded isolation of 6, but based on our various spectroscopic observations combined with the broad literature precedence for FLP-type activation of isocyanates, we propose a structure as depicted in Fig. 5a.

### Mechanistic insights

**Fig. 6:**
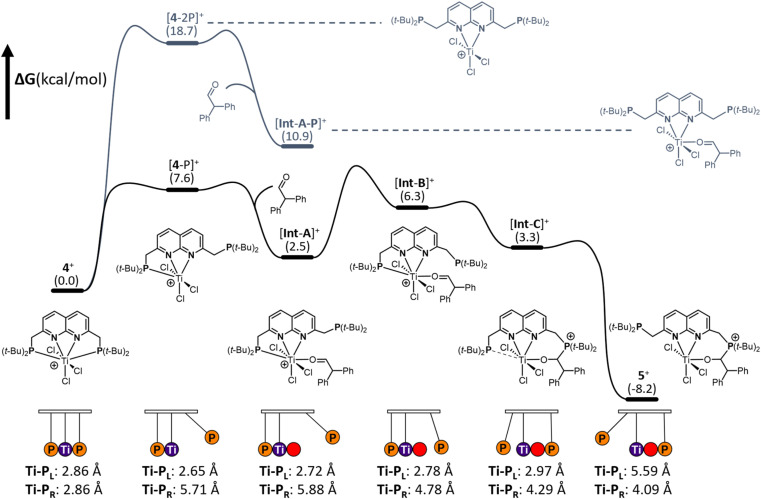
Gibbs free energy profile of the reaction of 4 with diphenylacetaldehyde yielding 5. The path in black shows the reaction pathway of aldehyde coordination upon dissociation of a single phosphine whereas the path in dark grey shows the less favoured reaction pathway of aldehyde coordination upon dissociation of both phosphines. The Newton's cradle-inspired diagram at the bottom shows the hemilabile role of the phosphine, supported by both Ti–P distances in Å throughout the reaction coordinate.

To gain insights into the mechanism of substrate activation, we resorted to Density Functional Theory (DFT) calculations (see ESI† for computational details, section S2.1). Having the solid-state structure of 5, we specifically targeted to investigate the aldehyde addition over the Ti–P bond. Based on the coordinative saturation and steric crowding around the 7-coordinate Ti centre in 4^+^, we reasoned that an associative pathway is unlikely. Dissociation of a phosphine arm is uphill by 7.6 kcal mol^−1^ (Fig. 6, [4-P]^+^) and proceeds barrierless (see ESI, section 2.20†).^44^

A structurally interesting observation is that upon phosphine dissociation the Ti–P bond length of the bound phosphine decreases from 2.86 Å to 2.65 Å. This is enabled by the chlorides bending away from the bound phosphine. This demonstrates that part of the ‘frustration’ of the system comes from both phosphines being bound. Unsurprisingly, dissociation of both arms is further energetically uphill by 18.7 kcal mol^−1^ (Fig. 6 [4-2P],^+^). From [4-P]^+^ we envisioned that two, non-concerted pathways can be followed: initial coordination of the aldehyde to the Ti followed by attack by the unbound phosphine, or attack of the dissociated phosphine on the aldehyde to form a zwitterionic phosphonium-alkoxide species followed by attack of the alkoxide on the Ti centre. Despite many attempts, the intermediates from direct attack of the phosphine failed to converge, either due to extrusion of the aldehyde from the zwitterionic species or due to preferential binding of the aldehyde to the Ti centre. We hypothesise that this is likely due to these structures being energetically inaccessible and as such we focussed our efforts on the route involving the coordination of the aldehyde to the Ti centre. Coordination of the aldehyde to the Ti centre in [4-P]^+^ is exergonic by 5.1 kcal mol^−1^ ([Int-A]^+^) and barrierless. [Int-A]^+^ is best characterised as the aldehyde adduct of [4-P]^+^, with an significant contribution of the alkoxide/carbocation resonance form of the aldehyde fragment based on increased C–O and Ti–P bond lengths (see ESI, section 2.13† for more detail). Dissociation of the phosphine donor from [Int-A]^+^ ([Int-A-P]^+^) was found to be energetically less favoured by 8.4 kcal mol^−1^. From [Int-A]^+^, the unbound phosphine rotates around the CH_2_–P bond to point the lone pair towards the aldehyde in [Int-B]^+^ (Fig. 6). A relaxed potential energy scan shows that this uphill process has a considerable barrier of 15 kcal mol^−1^ but no transition states could be found (see ESI, section S2.22†). From [Int-B]^+^ the reaction proceeds virtually barrierless^45^ towards [Int-C]^+^ (Fig. 6), which is structurally similar to 5^+^ before the hemilabile phosphine twists away. The O–C–H angle has decreased from 118° in [Int-B]^+^ to 111°, consistent with the rehybridisation of the carbon atom from sp^2^ to sp^3^. In agreement with formal addition of one of the C–O bonds over the Ti–P bond, the C–O bond distance increased from 1.25 Å in [Int-B]^+^ to 1.36 Å in [Int-C]^+^. Moreover, the Ti–O bond has decreased from 2.06 Å to 1.87 Å, in line with the more anionic nature of the O-donor in [Int-C]^+^. The trend of elongation of the Ti–P bond is continued, with the bond length increasing from 2.78 Å in [Int-B]^+^ to 2.92 Å in [Int-C]^+^. From [Int-C]^+^, dissociation of the phosphine donor is energetically downhill by 11.5 kcal mol^−1^, resulting in the formation of 5^+^.

From these calculations it is evident that both phosphines play a crucial role in the stabilisation of the [TiCl_3_]^+^ core. Initially, the Ti–P bond contracts upon dissociation of the other phosphine. This bond then progressively gets longer upon aldehyde binding and subsequent nucleophilic attack by the other phosphine, tailoring the bond length as a function of the change in electron density on Ti per intermediate. In this pendulum-like behaviour, the phosphines have a dual role: one phosphine acts as a nucleophile, while the other serves as a hemilabile donor that stabilises intermediates during the heterolytic bond activation process.

## Conclusion

We have demonstrated that the *^t^*^-Bu^PNNP ligand can stabilise an electrophilic [TiCl_3_]^+^ cation in a unique way that results in two equally long Ti–P bonds. This system presents the first experimental example of a theoretically predicted Frustrated Lewis Trio (FLT). Computational studies show that the Lewis acidic [TiCl_3_]^+^ in this system is stabilised by two Lewis basic phosphines, resulting in two equally frustrated Lewis acid/base pairs. In contrast to reported Ti/P based FLPs, wherein Ti is stabilised by electron donating ligands, this motif stabilises a highly Lewis acidic cation featuring electron-withdrawing chlorides. Similar to FLPs, the FLT can engage in the heterolytic activation of chemical bonds, but with a unique dual function for the Lewis basic phosphines. One of the phosphines acts as the nucleophile in the heterolytic activation of the substrate together with the Lewis acidic Ti. Notably, the other phosphine acts as a hemilabile ligand, tuning the Ti–P bond distance as a function of the electron density needed at the Ti centre during the bond activation process. The pendulum-like hemilability in this FLT provides a blueprint for the design of new ‘frustrated’ systems that mediate the stoichiometric and catalytic activation of chemical bonds.

## Data availability

The spectroscopic and computational data files that support the findings of this study are openly available in the Yoda data repository at DOI: https://doi.org/10.24416/UU01-Q4AU5Q.

## Author contributions

Synthesis and characterisation were done by E. K with support from D. v. T. and S. M. All crystallographic measurements and the corresponding data refinement was performed by M. L. Computations were done by E. K. and D. L. J. B. Project design, funding acquisition, administration, supervision and oversight were done by D. L. J. B. The original draft was written by E. K. and reviewing and editing was done by D. L. J. B. with contributions from all authors.

## Conflicts of interest

There are no conflicts to declare.

## Supplementary Material

SC-015-D3SC06789K-s001

SC-015-D3SC06789K-s002
