# Supplementary material for: Pendulum-like hemilability in a Ti-based frustrated Lewis Trio
Source: Chem Sci. 2024 Mar 8;15(15):5555–63. doi: 10.1039/d3sc06789k (PMC11023062; doi:10.1039/d3sc06789k)
Supplement: SC-015-D3SC06789K-s001 [file SC-015-D3SC06789K-s001.pdf]

Electronic Supplementary Material (ESI) for Chemical Science.
This journal is © The Royal Society of Chemistry 2024

SUPPORTING INFORMATION FOR

Pendulum-like Hemilability in a Ti-based Frustrated Lewis Trio

Errikos Kounalis,[a] Dylan van Tongeren, [a] Stanislav Melnikov,[a] Martin Lutz,[b], Daniël L. J. Broere*[a]

[a] Organic Chemistry & Catalysis, Institute for Sustainable and Circular Chemistry, Utrecht University, Universiteitsweg 99, 3584 CG, Utrecht, The Netherlands

[b] Structural Biochemistry, Bijvoet Center for Biomolecular Research, Faculty of Science, Utrecht University, Universiteitsweg 99, 3584 CG, Utrecht, The Netherlands

***Corresponding Author**
d.l.j.broere@uu.nl

Contents

1. Experimental methods: S4
   - 1.1 General considerations: S4
   - 1.2 Synthesis of [$^{t\text{-Bu}}$PNNPTiCl$_3$][TiCl$_5$·THF] (1): S5
   - 1.3 Synthesis of $^{t\text{-Bu}}$PNNPTiCl$_4$ (2): S8
   - 1.4 Synthesis of $^{t\text{-Bu}}$PNNPTiAu$_2$Cl$_6$ (3): S13
   - 1.5 Synthesis of [$^{t\text{-Bu}}$PNNPTiCl$_3$][BArF$_{24}$] (4): S19
   - 1.6 Van der Waals-Corrected Bond Lengths of 4 .... S27
   - 1.7 Synthesis of $^{t\text{-Bu}}$PNN^{Me}: S28
   - 1.8 Synthesis of $^{t\text{-Bu}}$PNN^{Me}TiCl$_4$: S33
   - 1.9 Halide abstraction from $^{t\text{-Bu}}$PNN^{Me}TiCl$_4$: S38
   - 1.10 Analysis of the halide abstraction from $^{t\text{-Bu}}$PNN^{Me}TiCl$_4$: S40
   - 1.11 Reaction of 4 with trans-stilbene oxide (5): S41
   - 1.12 Reaction of 4 with diphenylacetaldehyde: S46
   - 1.13 Reaction of 4 with phenyl isocyanate (6): S48
2. Computational Methods S52
   - 2.1 General remarks: S52
   - 2.2 Example input file for geometry optimisation: S52
   - 2.3 Example input file for SP calculations: S53
   - 2.4 Example input file for NBO calculations: S53
   - 2.5 XYZ Coordinates and energies of 4$^+$: S55
   - 2.6 XYZ Coordinates of the titanocene phosphinoaryloxide: S57
   - 2.7 NBO Donor-Acceptor interactions of the titanocene phosphinoaryloxide S58
   - 2.8 Computational Study Of [$^{t\text{-Bu}}$PNNP^{Me}TiCl$_3$]$^+$ and [$^{t\text{-Bu}}$PNN^{Me}TiCl$_3$]$^+$: S59
   - 2.9 XYZ coordinates of [$^{t\text{-Bu}}$PNNP^{Me}TiCl$_3$]$^+$: S61
   - 2.10 XYZ coordinates of [$^{t\text{-Bu}}$PNN^{Me}TiCl$_3$]$^+$: S62
   - 2.11 XYZ coordinates and energies of [4-P]$^+$: S63
   - 2.12 XYZ coordinates and energies of [4-2P]$^+$: S65
   - 2.13 Structural description of [Int-A]$^+$: S66
   - 2.14 XYZ coordinates and energies of [Int-A]$^+$: S67
   - 2.15 XYZ coordinates and energies of [Int-A-P]$^+$: S69
   - 2.16 XYZ coordinates and energies of [Int-B]$^+$: S71
   - 2.17 XYZ coordinates and energies of [Int-C]$^+$: S74
   - 2.18 XYZ coordinates and energies of 5$^+$: S76
   - 2.19 XYZ coordinates and energies of diphenylacetaldehyde: S78
   - 2.20 Potential Energy Scan of Phosphine Dissociation from 4$^+$: S79
   - 2.21 Potential Energy Scan of Aldehyde Association to [4-P]$^+$: S80

2.22 Potential Energy Scan of the Phosphine Arm Twisting in $[Int\text{-}A]^+$: S80
2.23 Potential Energy Scan of the P-C bond from $[Int\text{-}B]^+$ to $[Int\text{-}C]^+$: S81
2.24 Potential Energy Scan of the Phosphine Dissociation from $[Int\text{-}C]^+$: S82
3. Crystallographic Information S83
3.1 X-ray crystal structure determination of 1: S83
3.2 X-ray crystal structure determination of 2: S83
3.3 X-ray crystal structure determination of 3: S84
3.4 X-ray crystal structure determination of 4: S84
3.5 X-ray crystal structure determination of 5: S85
4. References S86

1. Experimental methods:

1.1 General considerations:

All manipulations were performed under inert atmosphere using standard Schlenk techniques or inside of a N_2-filled MBraun MB200B glovebox using anhydrous solvents and reagents, unless noted otherwise. Glassware was dried at 130 ˚C prior to use. Solvents were collected from an Mbraun MB-SPS-800 solvent purification system and stored over 4 Å molecular sieves, except for CH_2Cl_2, which was stored over 3 Å molecular sieves; chlorobenzene was heated at reflux over CaH_2, degassed by subjection to three freeze-pump-thaw cycles followed by backfilling with an N_2 atmosphere, canula filtration and passing over a pad of activated alumina before storing over 4 Å molecular sieves. Deuterated solvents were obtained from Cambridge Isotope Laboratories, degassed, and stored over 4 Å molecular sieves, except for CD_2Cl_2, which was stored over 3 Å molecular sieves. All commercial reagents were used as received and were obtained from Sigma Aldrich, Fluka or Acros.

$^{t\text{-Bu}}$**PNNP** was prepared according to literature procedure.[1] NMR data was recorded on an Agilent MRF 400 equipped with a OneNMR probe and Optima Tune system, 400 MHz Jeol EZCL G system with an HFX probe or a Varian VNMR-S-400 equipped with an AutoX probe. All resonances in ^{1}H-NMR were referenced to residual protio solvent peaks (7.16 for C_6D_6, 5.32 for CD_2Cl_2 and 2.09 the methyl proton resonance of toluene-d_8). All resonances in ^{13}C-NMR were referenced to the solvent and the respective ^{11}B-, ^{19}F- and ^{31}P-NMR spectra were referenced employing absolute referencing using the ^{1}H-NMR spectrum of the same sample. IR-data was recorded on a PerkinElmer SpectrumTwo Infrared Spectrophotometer equipped with an ATR-probe. ESI-MS was measured on an Advion Expression LCMS equipped with an automated Plate express TLC plate reader. Elemental analysis was performed by MEDAC ltd. In the United Kingdom.

1.2 Synthesis of [$^{t\text{-Bu}}$PNNPTiCl$_3$][TiCl$_5$·THF] (1):

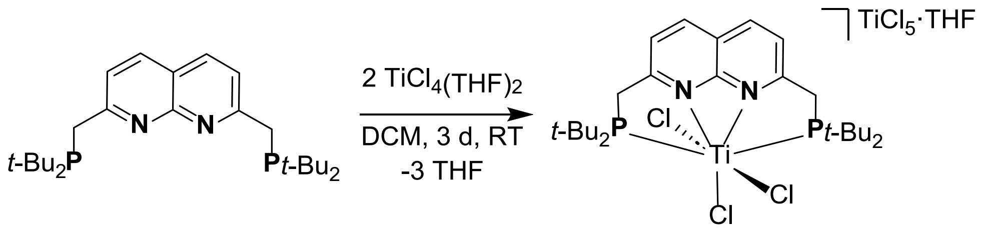

A solution of $^{t\text{-Bu}}$**PNNP** (100.0 mg,224 µmol) in CH_2Cl_2 (5 mL) was added dropwise to a stirring solution of $TiCl_4(THF)_2$ (150.0 mg, 449 µmol) in CH_2Cl_2 (4 mL) at ambient temperature, yielding a dark green mixture. The mixture was left to stir for 3 days and was filtered over a pipette filter. The black residue was discarded and the green filtrate* was concentrated in vacuum to 107.0 mg of a green solid consisting of an inseparable mixture of the target compound and $^{t\text{-Bu}}$**PNNP**$TiCl_4$.

* Storing this filtrate at -40 °C yielded green crystals in 2h suitable for XRD analysis.

^{1}H-NMR (400 MHz, CD_2Cl_2, 298 K): δ 8.86 (d, $^3J_{H,H}$ = 8.5 Hz, 2H), 8.11 (d, $^3J_{H,H}$ = 8.5 Hz, 2H), 3.82 (dd, $^2J_{H,P}$ = 4.0 Hz, $^4J_{H,P}$ = 3.8 Hz, 4H), 1.54 (dd, $^3J_{H,P}$ = 6.6 Hz, $^5J_{H,P}$ = 6.4 Hz, 36H).

^{31}P{^{1}H}-NMR (162 MHz, C_6D_6, 298 K): δ 77.4 (s).

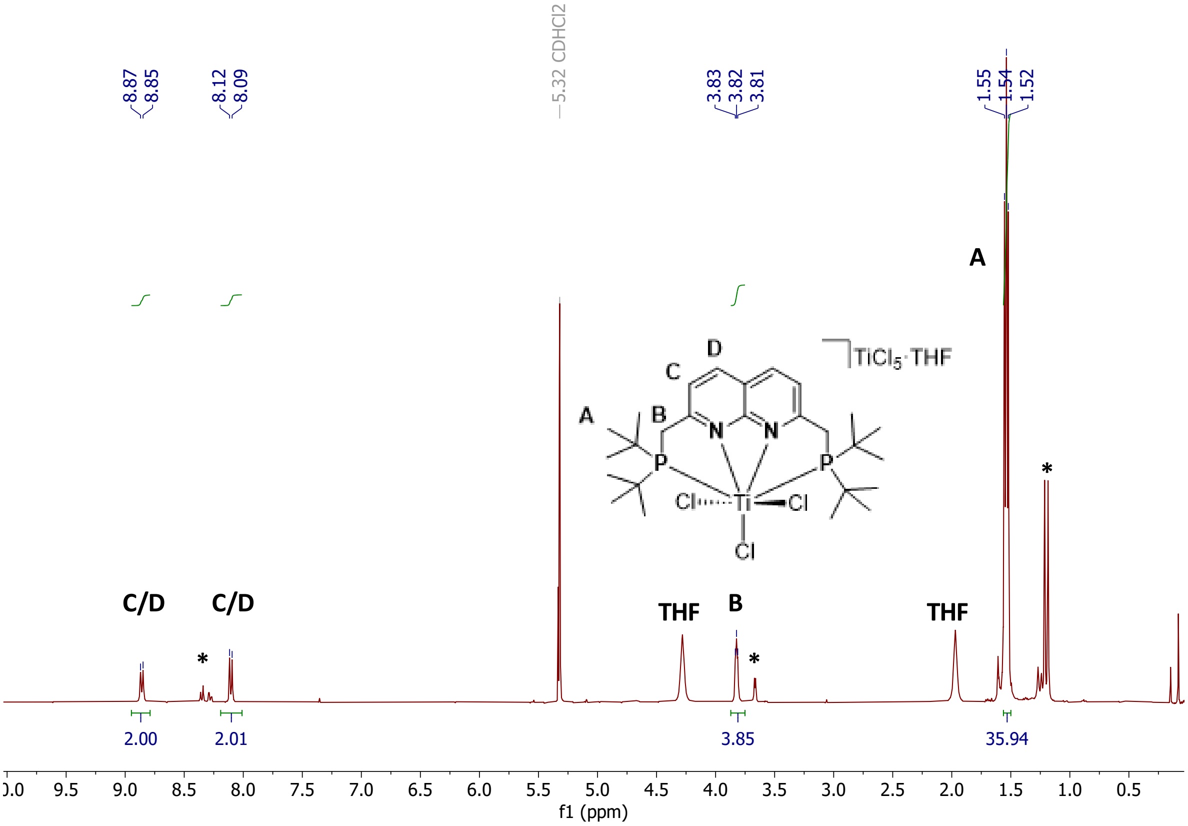

Figure S1: ^{1}H-NMR spectrum of [$^{t\text{-Bu}}$**PNNP**$TiCl_3$][$TiCl_5$·THF] in CD_2Cl_2 at 25 °C. Resonances marked with a * are attributed to $^{t\text{-Bu}}$**PNNP**$TiCl_4$.

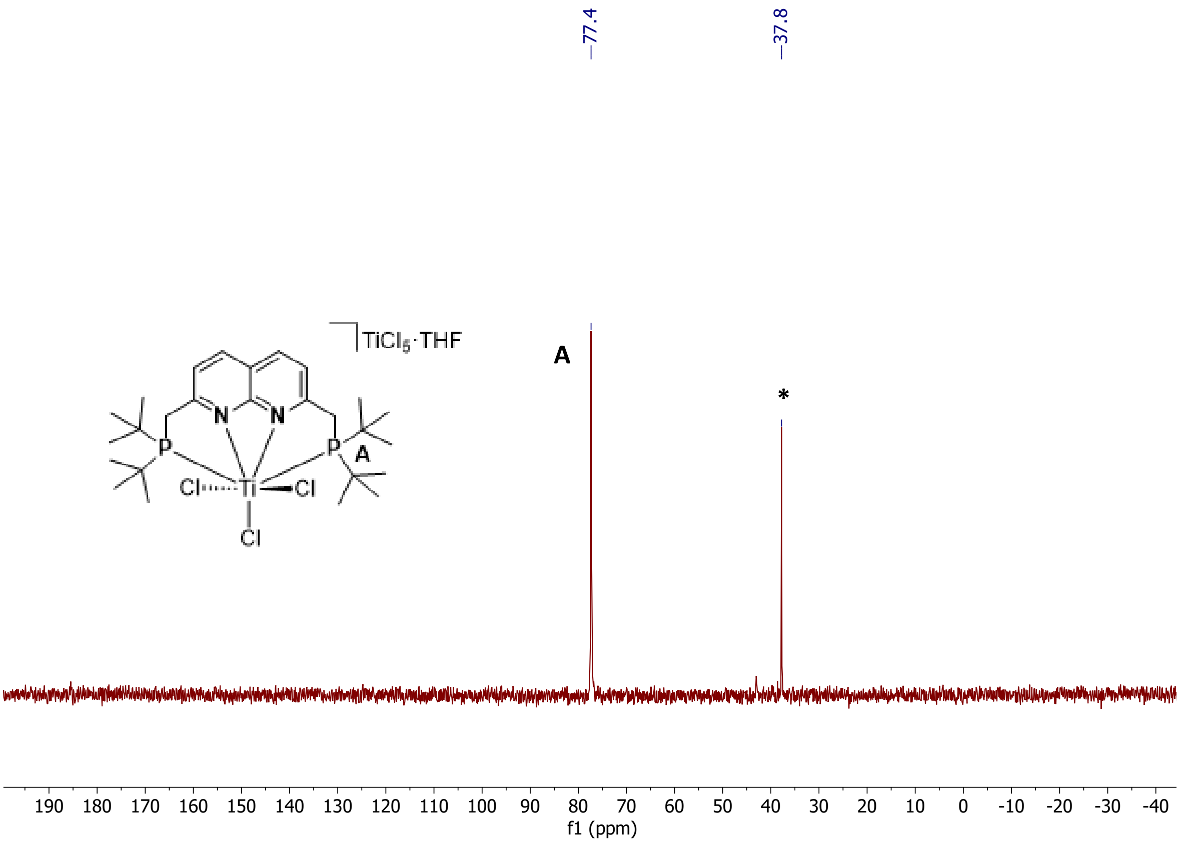

Figure S2: $^{31}P\{^{1}H\}$-NMR spectrum of [$^{t\text{-Bu}}$**PNNP**$TiCl_3$][$TiCl_5$·THF] in CD_2Cl_2 at 25 °C. Resonance marked with a * is attributed to $^{t\text{-Bu}}$**PNNP**$TiCl_4$.

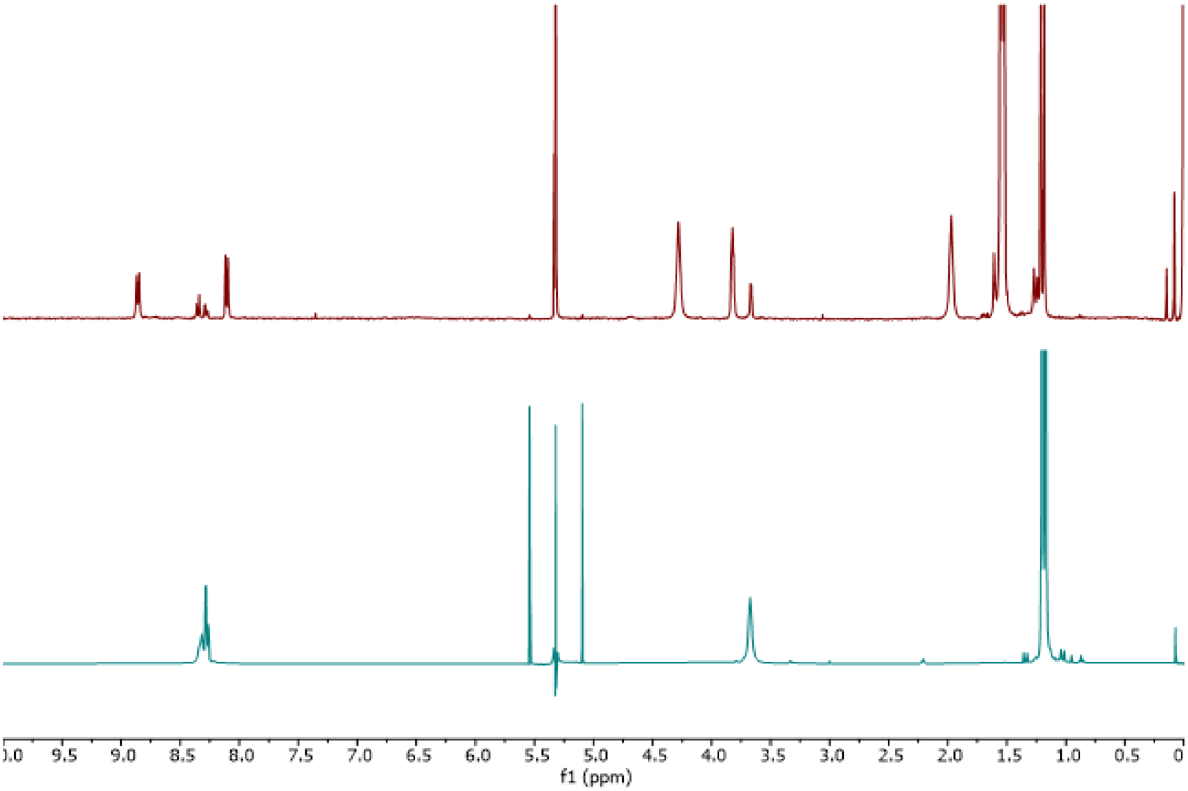

Figure S3: Stacked ^{1}H-NMR spectra of **1** (top, CD_2Cl_2) and **2** (bottom, CH_2Cl_2) at 25 °C.

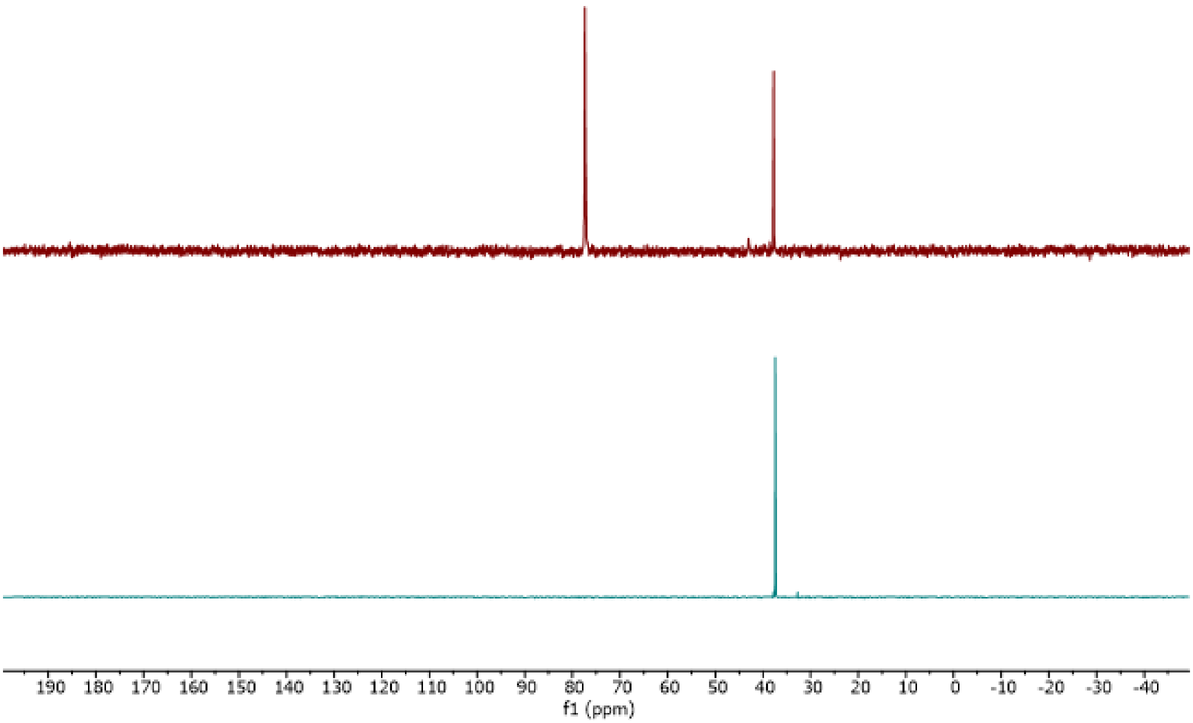

Figure S4: Stacked $^{31}P\{^{1}H\}$-NMR spectra of **1** (top, CD_2Cl_2) and **2** (bottom, CH_2Cl_2) at 25 °C.

1.3 Synthesis of $^{t\text{-}Bu}$PNNPTiCl$_4$ (2):

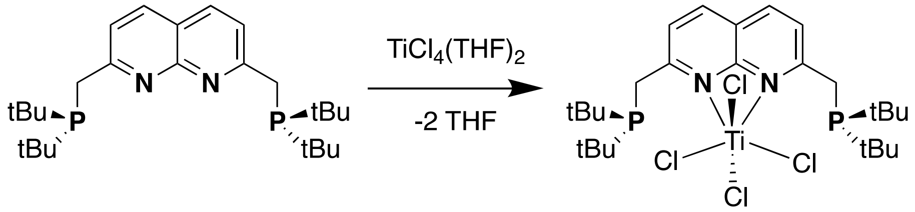

A solution of $TiCl_4(THF)_2$ (80.7 mg, 242 µmol) in CH_2Cl_2 (3 mL) was added dropwise to a stirring solution of $^{t\text{-}Bu}$**PNNP** (108.0 mg, 242 µmol) in CH_2Cl_2 (3 mL) at ambient temperature, resulting in a solution progressively turning darker orange. After 4 h the volatiles were removed under vacuum. The residue was washed with cold pentane (3 x 1 mL) and dried under vacuum to give a brown solid (125.6 mg, 82%). Single crystals suitable for XRD were grown from a 1:4 benzene/pentane mixture at –40 °C.

Note: At larger scales (>100 mg) we found that reactions using $TiCl_4$ provides cleaner product than when $TiCl_4(THF)_2$ is used.

^{1}H-NMR (400 MHz, C_6D_6, 298 K): δ 7.88 (dd, $^3J_{H,H}$ = 8.6 Hz, $^4J_{H,P}$ = 2.7 Hz, 2H), 7.19 (d, $^3J_{H,H}$ = 8.6 Hz, 2H), 3.77 (d, $^2J_{H,P}$ = 3.8 Hz, 4H), 1.12 (d, $^3J_{H,P}$ = 11.5 Hz, 36H).

^{13}C{^{1}H}-NMR (101 MHz, C_6D_6, 298 K): δ 166.3 (d, $^2J_{C,P}$ = 16.1 Hz), 154.1 (t, $^4J_{C,P}$ = 1.0 Hz), 137.3, 125.7 (d, $^3J_{C,P}$ = 18.7 Hz), 118.1, 32.5 (d, $^1J_{C,P}$ = 21.4 Hz), 31.5 (d, $^1J_{C,P}$ = 26.5 Hz), 29.8 (d, $^2J_{C,P}$ = 13.6 Hz).

^{31}P{^{1}H}-NMR (162 MHz, C_6D_6, 298 K): δ 38.0 (s).

Anal. Calcd. For $C_{26}H_{44}Cl_4N_2P_2Ti$: C, 49.08; H, 6.97; N, 4.40. **Found** C, 49.40; H, 7.29; N, 4.34.

IR-ATR (cm^{-1}): 3066 (w), 2940 (s), 2896 (m), 2863 (m), 1618 (m), 1603 (s), 1558 (w), 1505 (s), 1470 (m), 1433 (w), 1388 (m), 1367 (m), 1311 (w), 1251 (m), 1224 (w), 1175 (w), 1150 (w), 1016 (w), 934 (w), 859 (w), 814 (w), 775 (w), 736 (w), 600 (w).

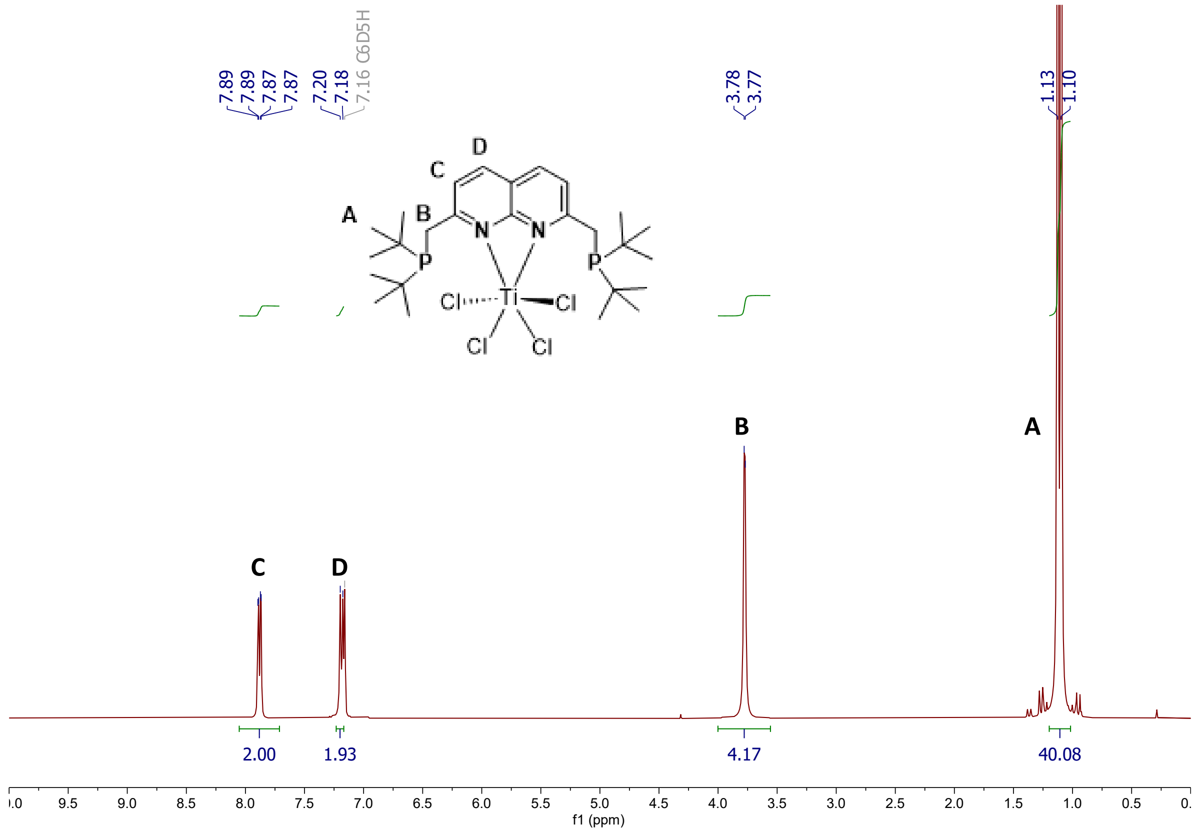

Figure S5: ^{1}H-NMR spectrum of $^{t\text{-Bu}}$**PNNP**$TiCl_4$ in C_6D_6 at 25 °C.

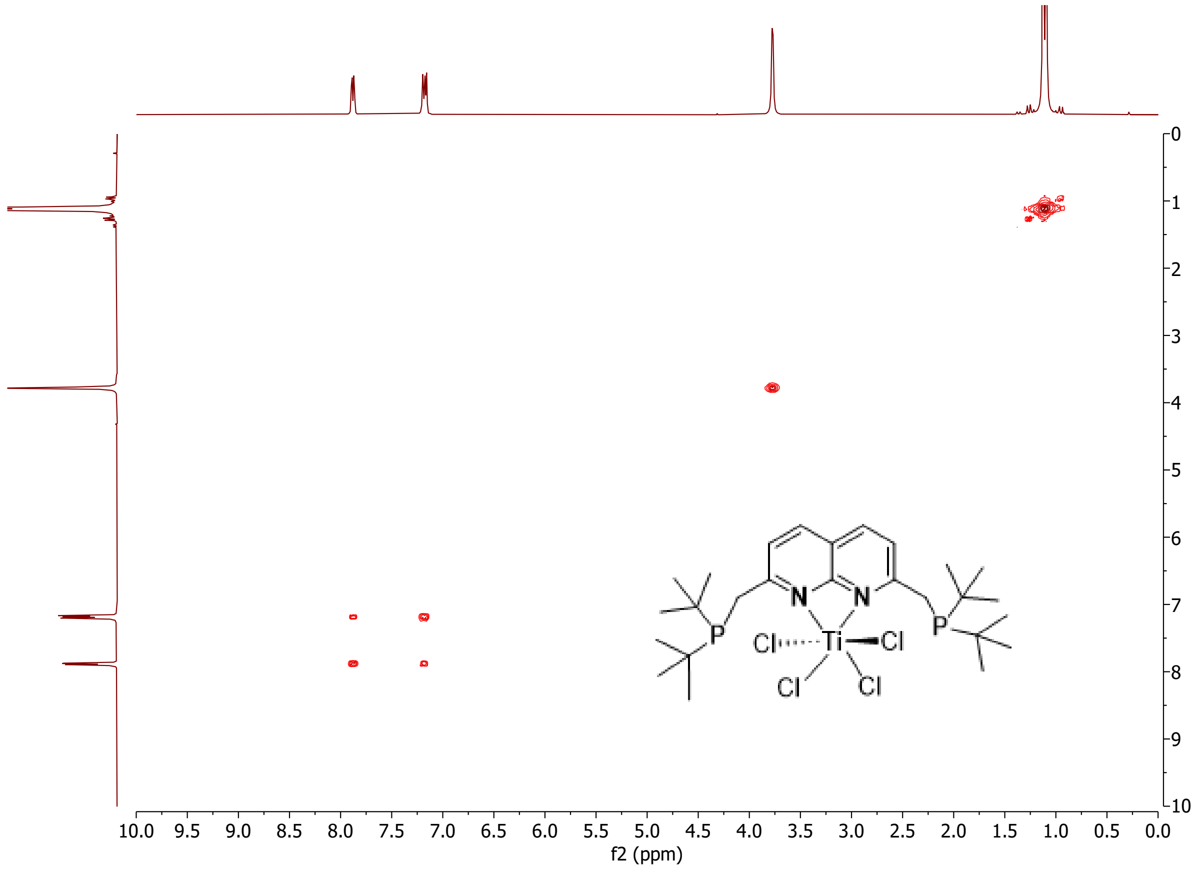

Figure S6: ^{1}H-NMR COSY spectrum of $^{t\text{-Bu}}$**PNNP**$TiCl_4$ in C_6D_6 at 25 °C.

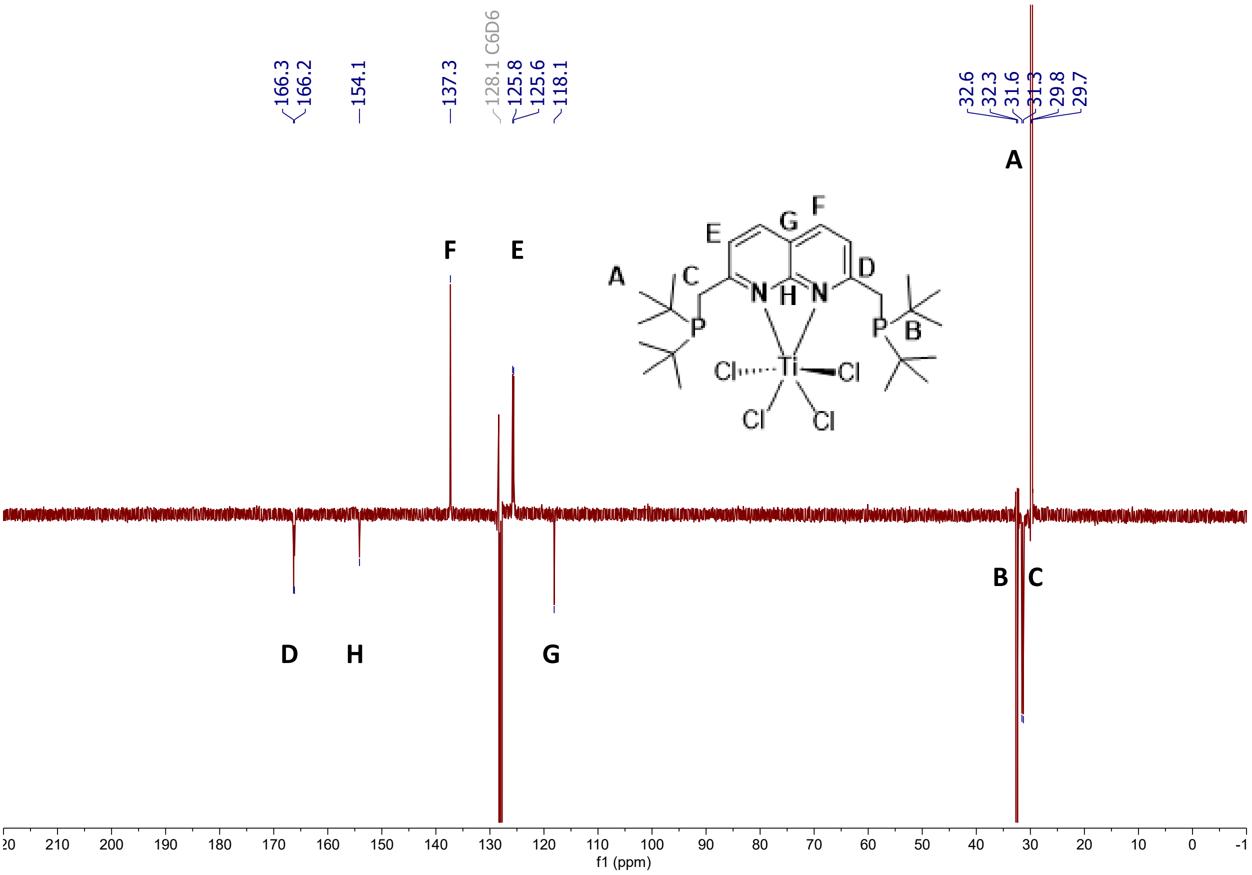

Figure S7: $^{13}C\{^{1}H\}$-NMR (APT) spectrum of $^{t\text{-Bu}}$**PNNP**$TiCl_4$ in C_6D_6 at 25 °C.

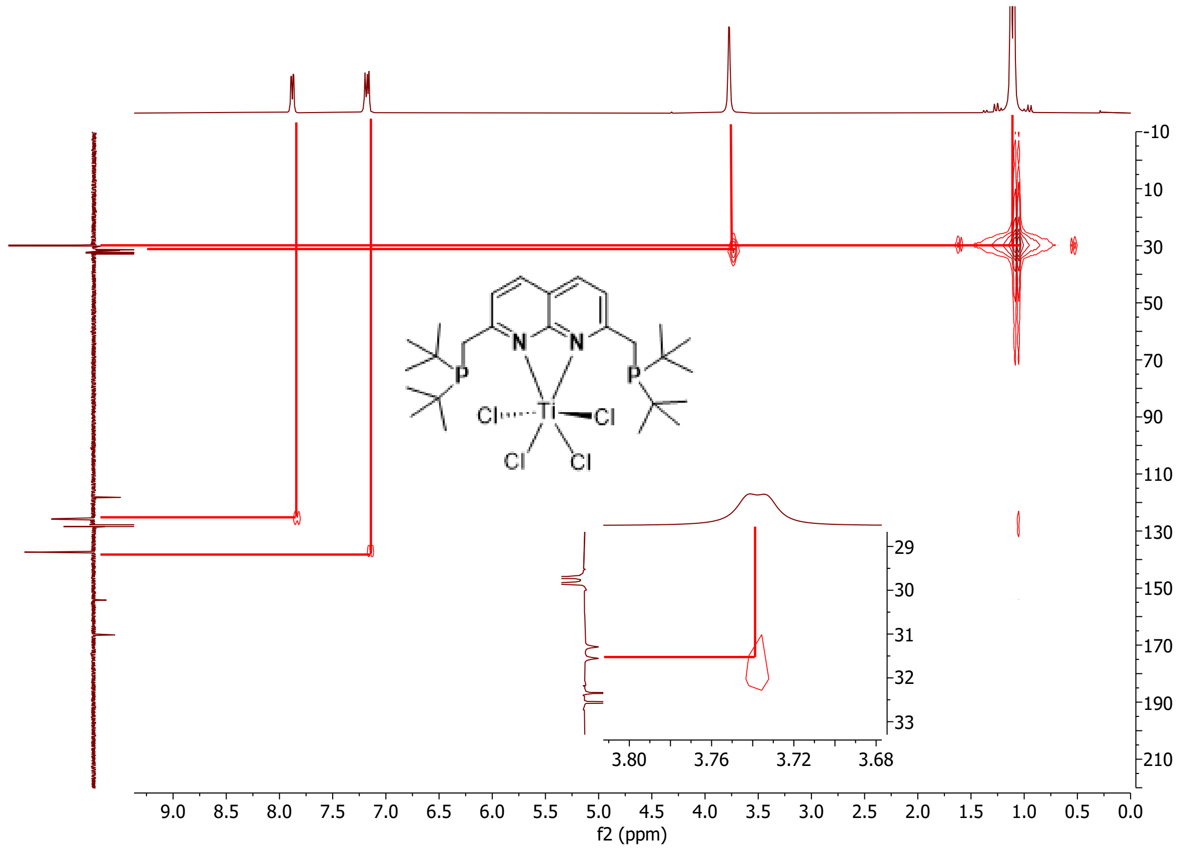

Figure S8: ^{1}H-^{13}C HMQC spectrum of $^{t\text{-Bu}}$**PNNP**$TiCl_4$ in C_6D_6 at 25 °C, inset shows a zoomed in view of the correlation of the methylene proton and carbon resonances.

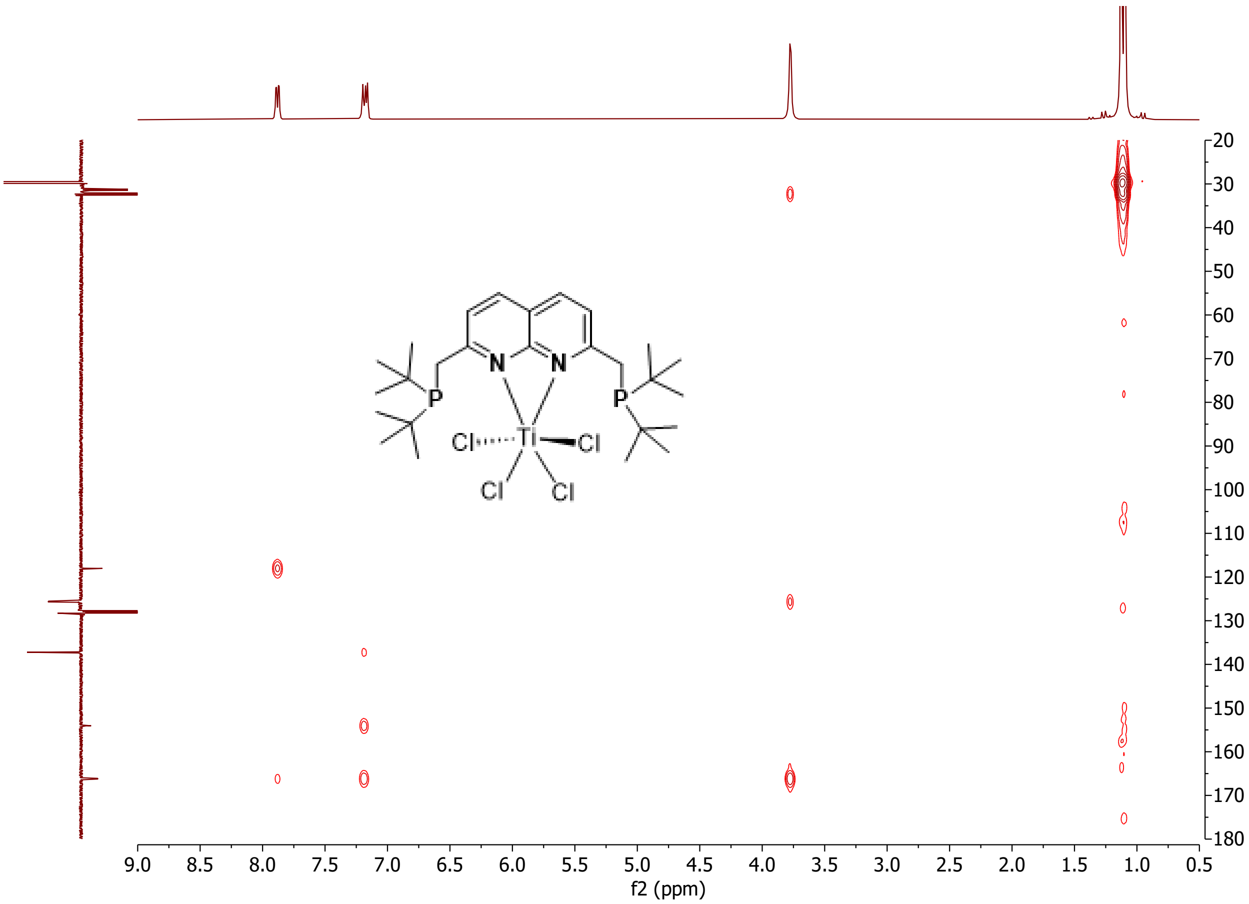

Figure S9: The ^{1}H-^{13}C HMBC spectrum of ***t*-BuPNNP**$TiCl_4$ in C_6D_6 at 25 °C.

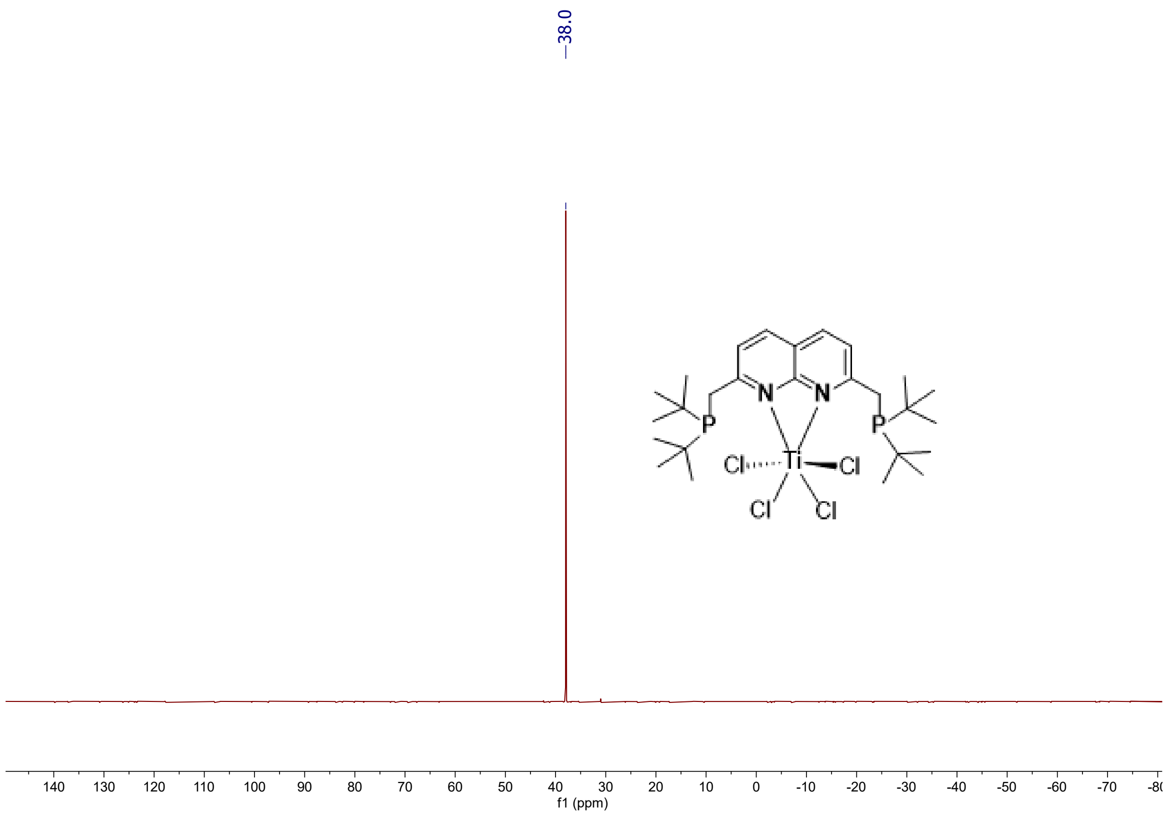

Figure S10: The $^{31}P\{^{1}H\}$-NMR spectrum of ***t*-BuPNNP**$TiCl_4$ in C_6D_6 at 25 °C.

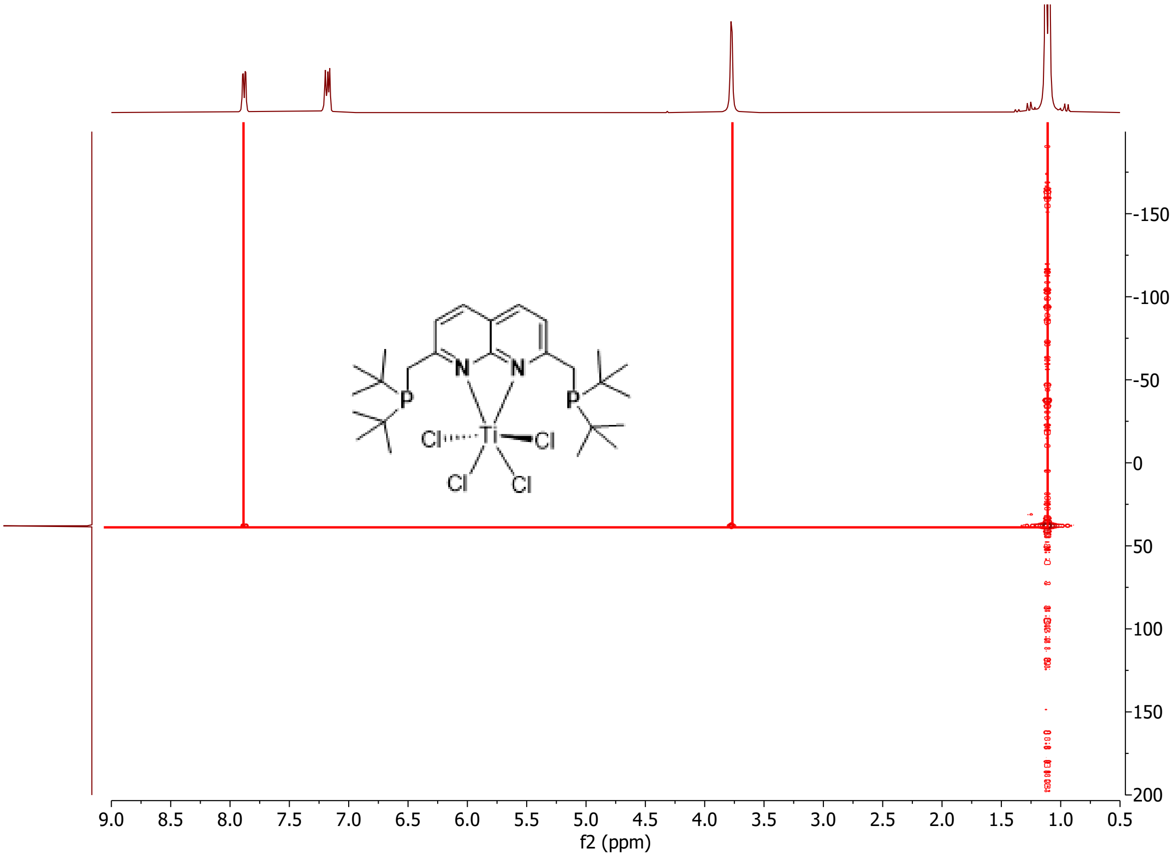

Figure S11: ^{1}H-^{31}P HMBC spectrum of $^{t\text{-Bu}}$**PNNP**$TiCl_4$ in C_6D_6 at 25 °C.

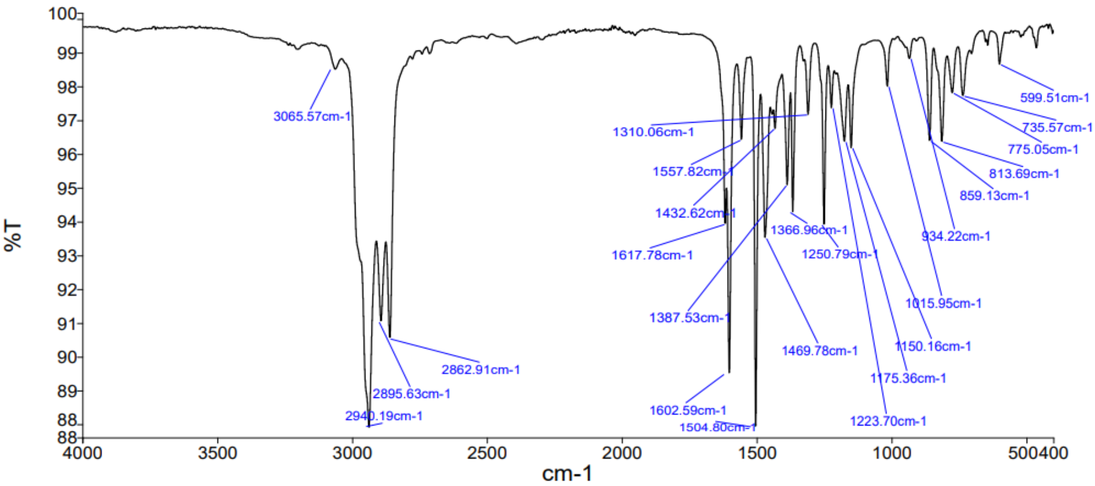

Figure S12: ATR-IR spectrum of $^{t\text{-Bu}}$**PNNP**$TiCl_4$ measured as a film under N_2 flow at 25 °C.

1.4 Synthesis of $^{t\text{-Bu}}$PNNPTiAu$_2$Cl$_6$ (3):

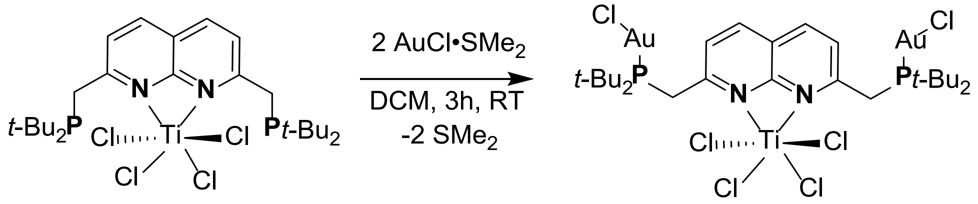

A solution of $^{t\text{-Bu}}$**PNNP**TiCl$_4$ (105.5 mg, 166 µmol) in CH_2Cl_2 (3 mL) was added dropwise to a stirring suspension of AuCl·SMe$_2$ (97.7 mg, 332 µmol) in CH_2Cl_2 (3 mL). After stirring the yellow suspension at ambient temperature for 3 h, the mixture was filtered over a pad of Celite® and concentrated under vacuum to a volume of approx. 3 mL. The solution was layered with pentane (4 mL), which led to the formation of yellow crystals over the course of a weekend (~65 h). The crystals were isolated by decanting the mother liquor off and washing the crystals with cold (–40 °C) pentane (3 x 1.5 mL) at which point the washings were colourless. The crystals were subsequently dried *in vacuo* to a yellow solid (161.3 mg, 88%).

^{1}H-NMR (400 MHz, C_6D_6, 298 K): δ 8.56 (d, $^3J_{H,H}$ = 8.6 Hz, 2H), 7.02 (d, $^3J_{H,H}$ = 8.6 Hz, 2H), 3.81 (d, $^2J_{H,P}$ = 11.8 Hz, 4H), 0.84 (d, $^3J_{H,P}$ = 15.8 Hz, 36H).

^{13}C{^{1}H}-NMR (101 MHz, C_6D_6, 298 K): δ 160.5 (d, $^2J_{C,P}$ = 16.1 Hz), 153.5 (s), 139.0 (s), 126.6 (d, $^2J_{C,P}$ = 6.2 Hz), 119.6 (s), 36.7 (d, $^1J_{C,P}$ = 25.6 Hz), 29.5 (d, $^1J_{C,P}$ = 23.1 Hz), 29.3 (d, $^2J_{C,P}$ = 5.3 Hz).

^{31}P{^{1}H}-NMR (162 MHz, C_6D_6, 298 K): δ 72.5 (s).

Anal. Calcd. For $C_{26}H_{44}Au_2Cl_6N_2P_2Ti$: C, 28.36; H, 4.03; N, 2.54. **Found** C, 29.71; H, 4.64; N, 2.19.

IR-ATR (cm^{-1}): 3058 (w), 2958 (s), 2985 (m), 1603 (s), 1557 (w), 1506 (s), 1472 (m), 1389 (m), 1372 (m), 1308 (w), 1255 (m), 1177 (m), 1021 (w), 858 (m), 816 (w), 733 (w), 613 (w).

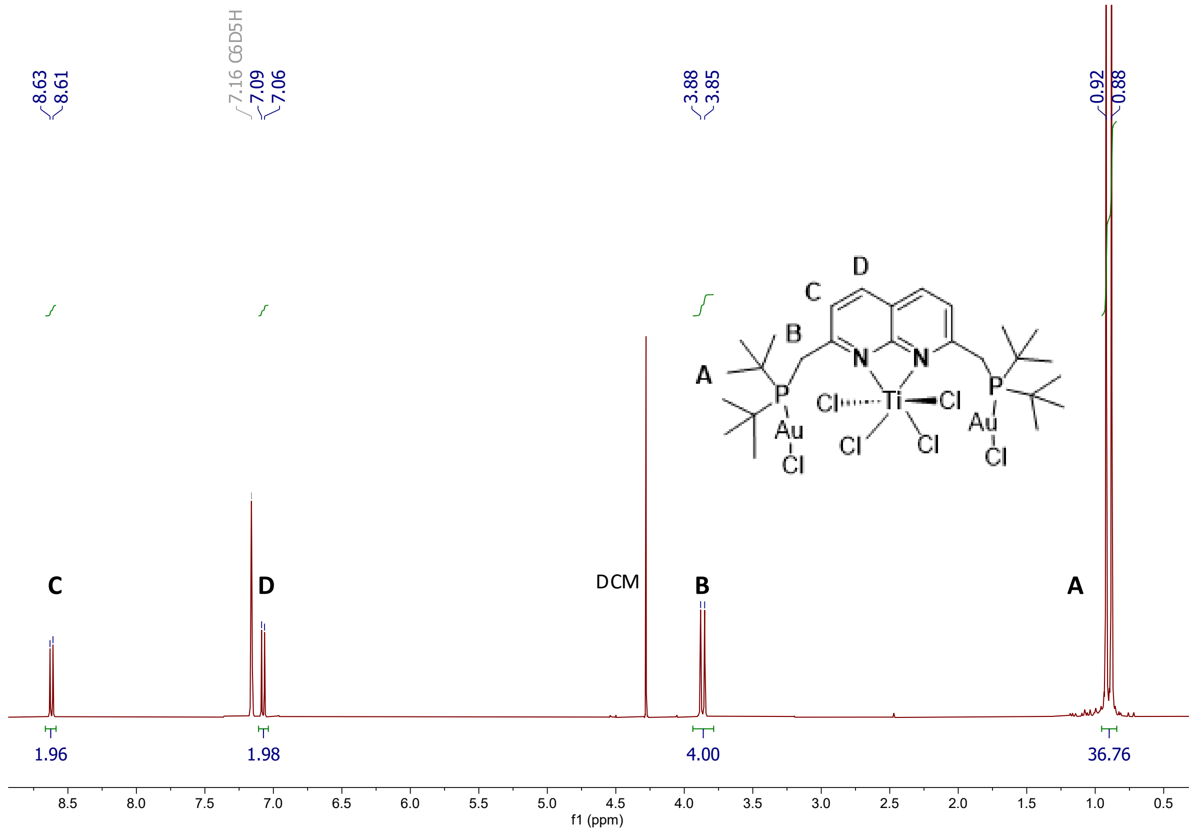

Figure S13: ^{1}H-NMR spectrum of ***t*-Bu****PNNP**TiAu$_2$Cl$_6$ in C$_6$D$_6$ at 25 °C.

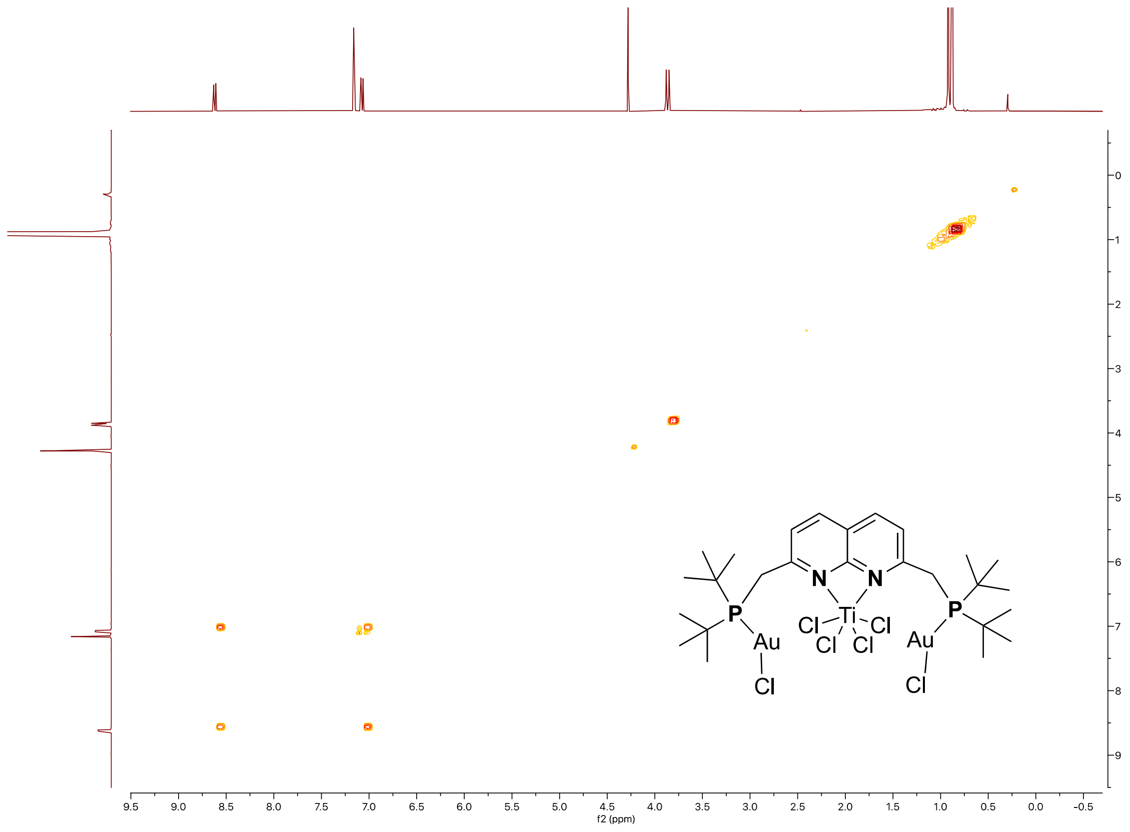

Figure S14: ^{1}H-NMR COSY spectrum of ***t*-Bu****PNNP**TiAu$_2$Cl$_6$ in C$_6$D$_6$ at 25 °C.

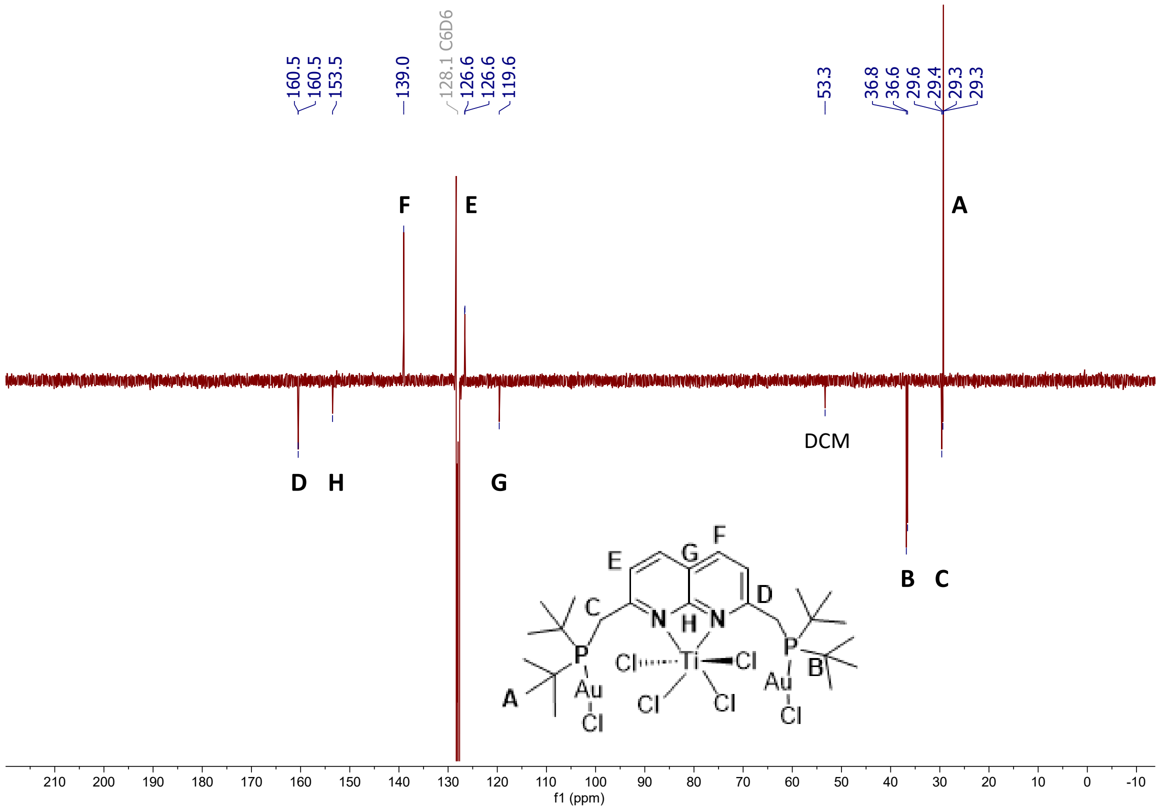

Figure S15: The $^{13}C\{^{1}H\}$-NMR (APT) spectrum of ***t*-Bu****PNNP**TiAu$_2$Cl$_6$ in C$_6$D$_6$ at 25 °C.

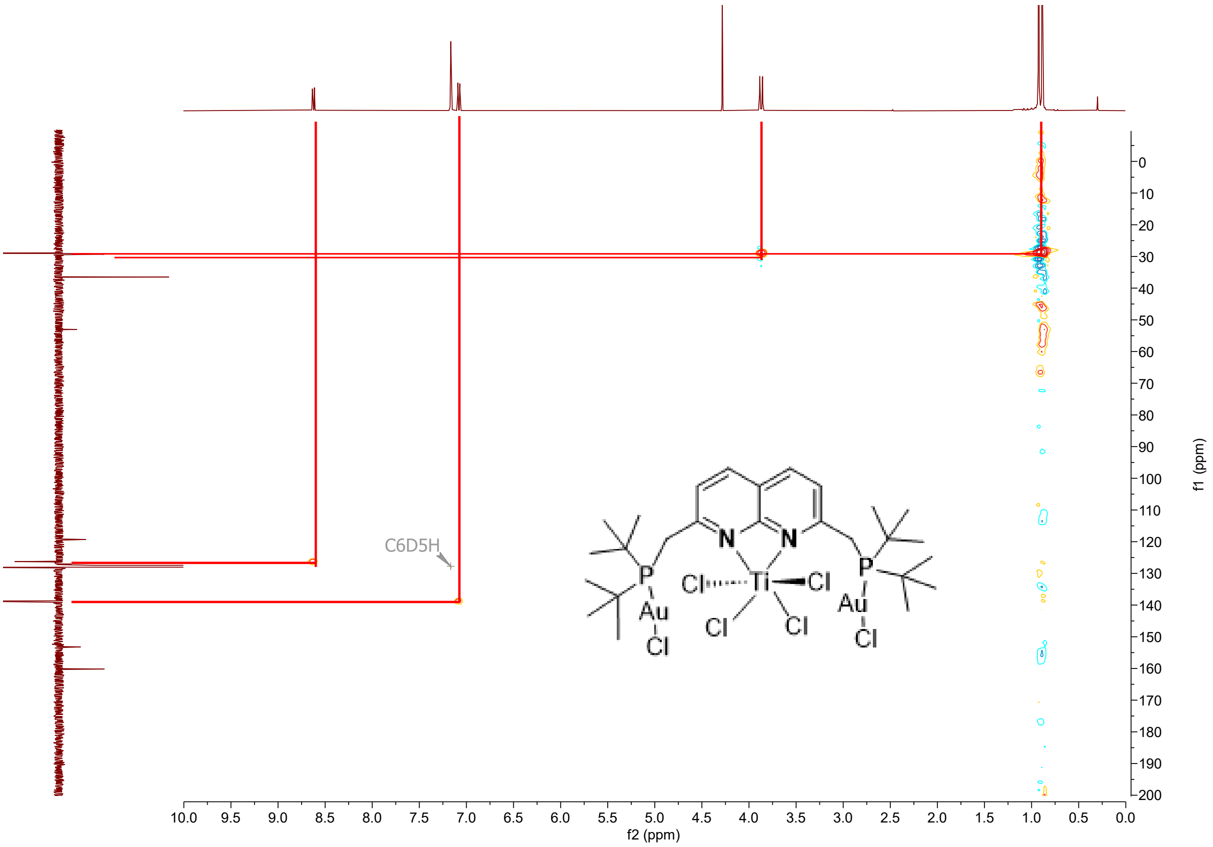

Figure S16: The ^{1}H-^{13}C HMQC spectrum of ***t*-Bu****PNNP**TiAu$_2$Cl$_6$ in C$_6$D$_6$ at 25 °C.

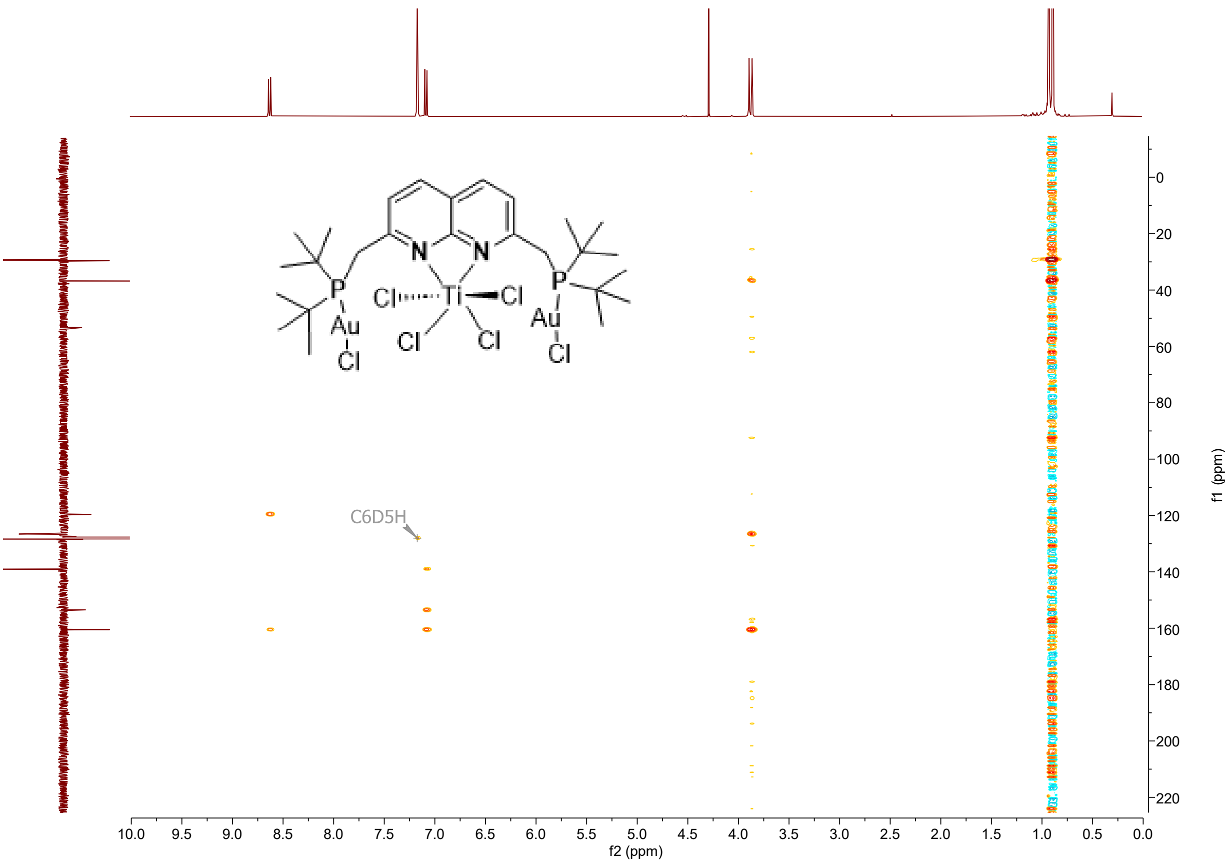

Figure S17: ^{1}H-^{13}C HMBC spectrum of ***t*-BuPNNP**$TiAu_2Cl_6$ in C_6D_6 at 25 °C.

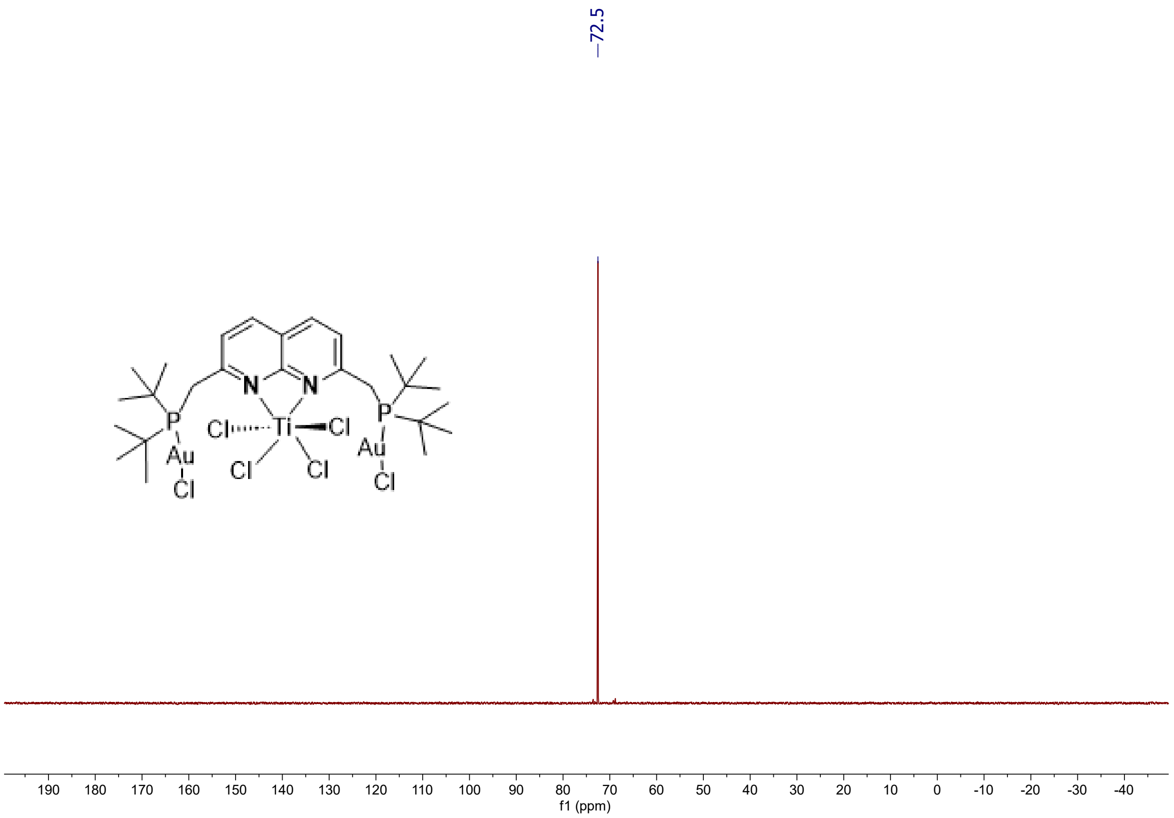

Figure S18: $^{31}P\{^{1}H\}$-NMR spectrum of ***t*-BuPNNP**$TiAu_2Cl_6$ in C_6D_6 at 25 °C.

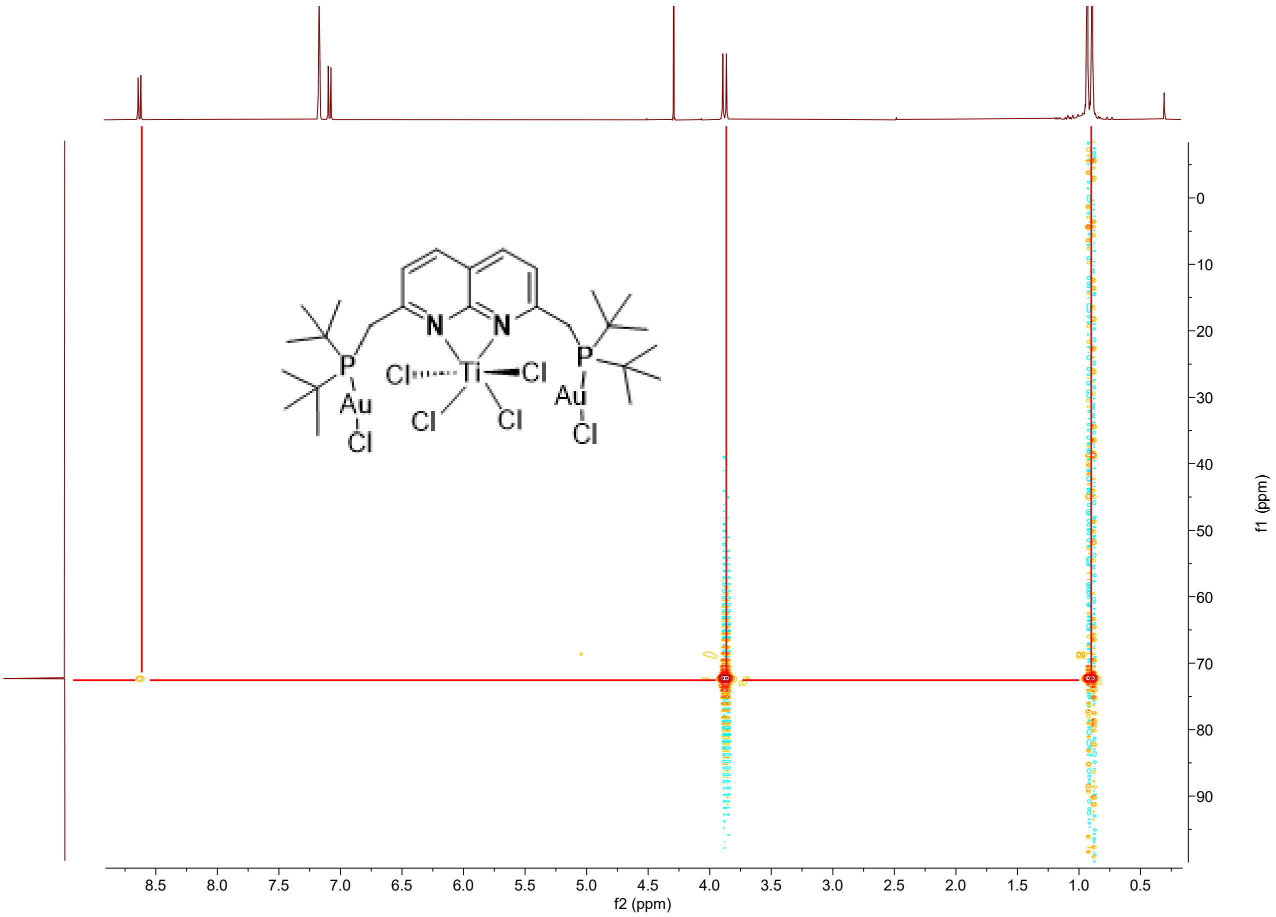

Figure S19: ^{1}H-^{31}P HMBC spectrum of ***t*-Bu****PNNP**$TiAu_2Cl_6$ in C_6D_6 at 25 °C.

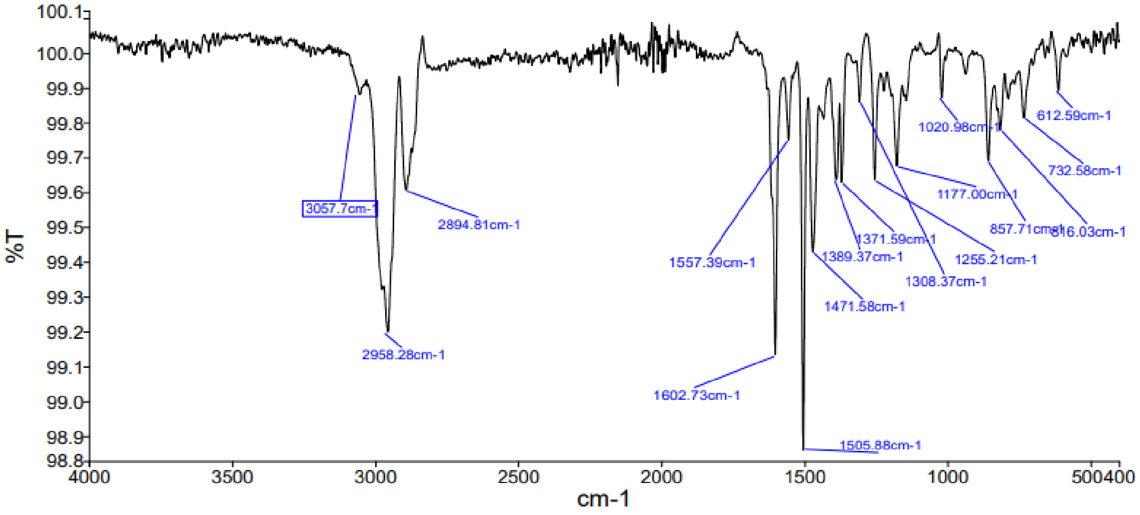

Figure S20: ATR-IR spectrum of ***t*-Bu****PNNP**$TiAu_2Cl_6$ measured as a film under N_2 flow at 25 °C.

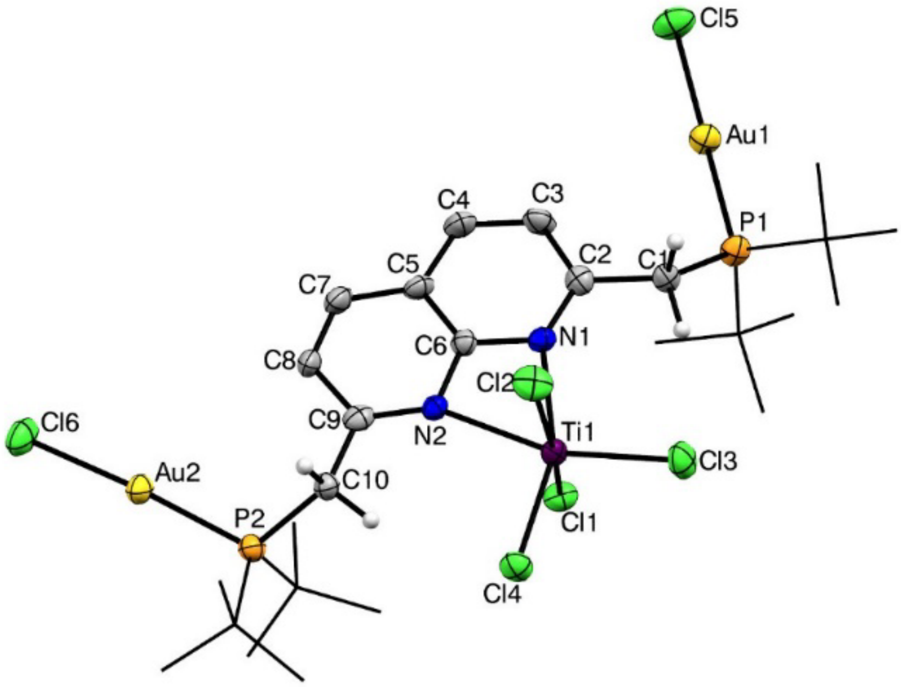

Figure S21: Displacement ellipsoid plot (50% probability) of **3** in the crystal. Most hydrogen atoms and co-crystallised pentane molecule are omitted and *t*-Bu groups on P are depicted as wireframe for clarity. Selected bond distances (Å): **Ti1-Cl1** 2.2987(17), **Ti1-Cl2** 2.2616(18), **Ti1-Cl3** 2.2376(17), **Ti1-Cl4** 2.2322(16), **Ti1-N1** 2.243(4), **Ti1-N2** 2.283(4), **Au1-Cl5** 2.2873(15), **Au2-Cl6** 2.2837(15), **Au1-P1** 2.2464(15), **Au2-P2** 2.2403(15).

1.5 Synthesis of [$^{t\text{-Bu}}$PNNPTiCl$_3$][BArF$_{24}$] (4):

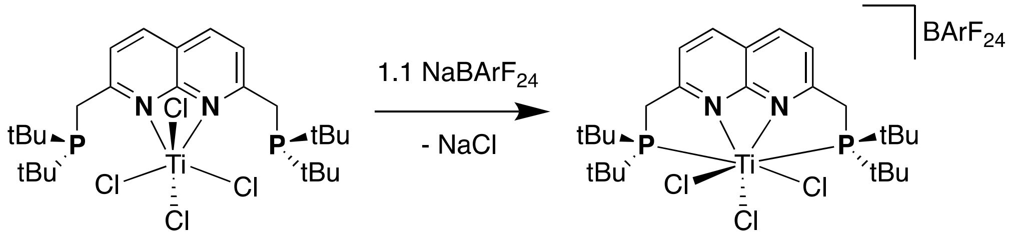

A slurry of NaBArF$_{24}$ (39.3 mg, 44 µmol) in chlorobenzene (3 mL) was added dropwise to a stirring solution of $^{t\text{-Bu}}$**PNNP**TiCl$_4$ (25.7 mg, 40 µmol) in chlorobenzene (1.5 mL) at ambient temperature, resulting in a dark green solution. The mixture was left to stir for 16 h and subsequently the mixture was filtrated over a pipette containing a filter paper plug. The filtrate was concentrated under vacuum to a volume of ~ 2 mL after which ~ 2 mL of pentane was added. The solution was stored in the freezer at –40 °C and large dark crystals grew over the course of 65 h. The crystals were isolated by decanting off the mother liquor and were subsequently washed with cold (–40 °C) hexane (3 x 1.5 mL) at which point the washings were colourless. Afterwards, the crystals were ground and dried extensively in vacuo to give the target compound as a dark solid (42.3 mg, 72 %).

^{1}H-NMR (400 MHz, C$_6$D$_6$, 298 K): δ 8.41 (s, broad, 8H), 7.62 (s, broad, 4H), 2.78 (dd, $^{2}J_{H,P}$ = 4.2 Hz, $^{4}J_{H,P}$ = 3.8 Hz, 4H), 1.12 (dd, $^{3}J_{H,P}$ = 6.7 Hz, $^{5}J_{H,P}$ = 6.3 Hz, 36H).

^{11}B{^{1}H}-NMR (128 MHz, C$_6$D$_6$, 298 K): δ -6.9 (s).

^{13}C{^{1}H}-NMR (101 MHz, C$_6$D$_6$, 298 K): δ 162.9 (1:1:1:1 q, $^{1}J_{B,C}$ = 49.7 Hz), 162.7 (dd, $^{2}J_{P,C}$ = 4.6 Hz, $^{3}J_{P,C}$ = 3.6 Hz), 153.6 (t, $^{3}J_{P,C}$ = 6.9 Hz), 139.7, 135.4, 130.1 (q, $^{2}J_{F,C}$ = 32.5 Hz), 125.2 (q, $^{1}J_{F,C}$ = 272.5 Hz), 123.6, 118.2*, 118.2*, 37.8, 31.0 (t, $^{1}J_{P,C}$ = 3.6 Hz), 29.5.

^{19}F-NMR (376 MHz, C$_6$D$_6$, 298 K): δ -62.0 (s).

^{31}P{^{1}H}-NMR (162 MHz, C$_6$D$_6$, 298 K): δ 76.7 (s).

*Two overlapping resonances, confirmed through 2D-NMR experiments

The reactive nature of the compound precluded obtaining a satisfactory result for Elemental analysis.

IR-ATR (cm^{-1}): 3068 (w), 2963 (m), 2928 (m), 2874 (m), 2551 (w), 2397 (w), 1722 (m), 1633 (w), 1608 (m), 1507 (w), 1472 (w), 1354 (m), 1276 (s), 1161 (m), 1124 (s), 1021 (w), 947 (w), 887 (w), 839 (w), 814 (w), 745 (w), 713 (w), 682 (w), 670 (w).

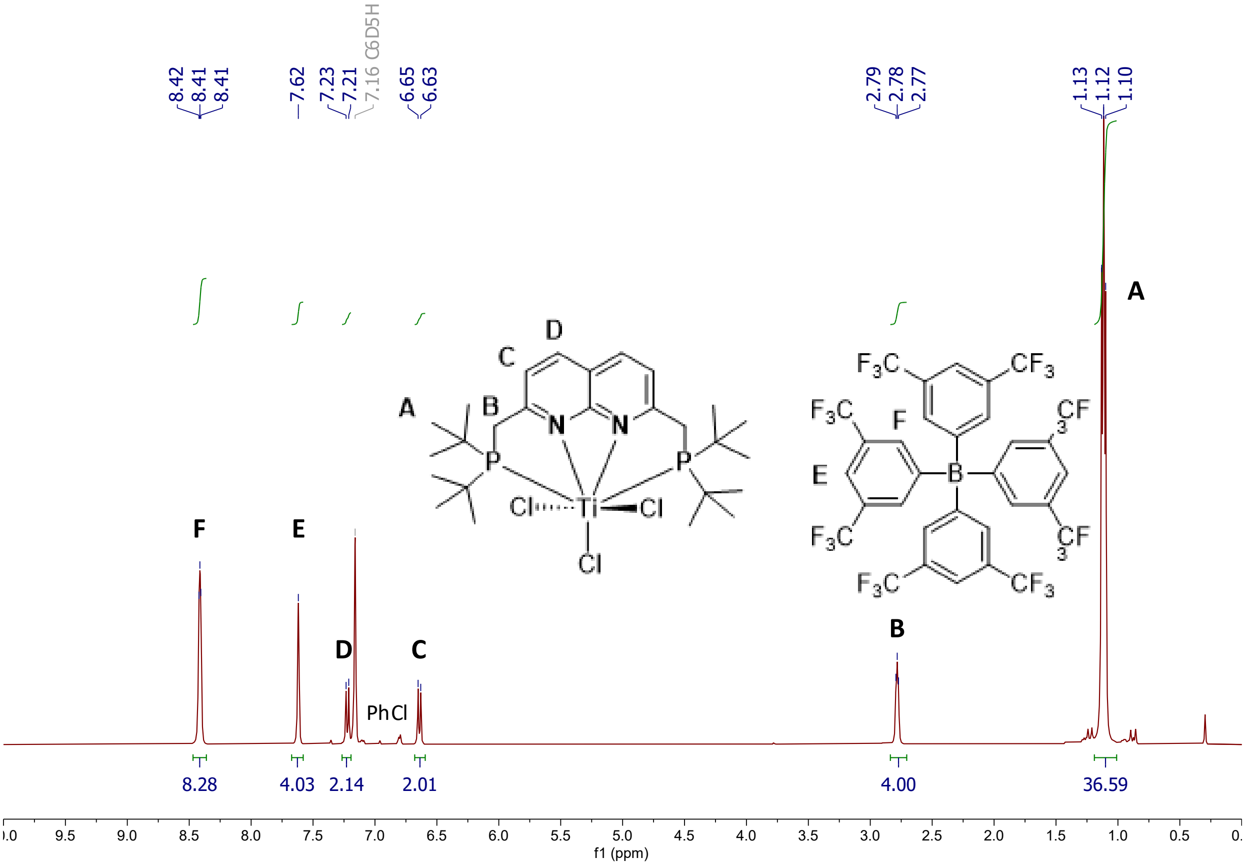

Figure S22: ^{1}H-NMR spectrum of **4** in C_6D_6 at 25 °C, measured in a quartz NMR tube.

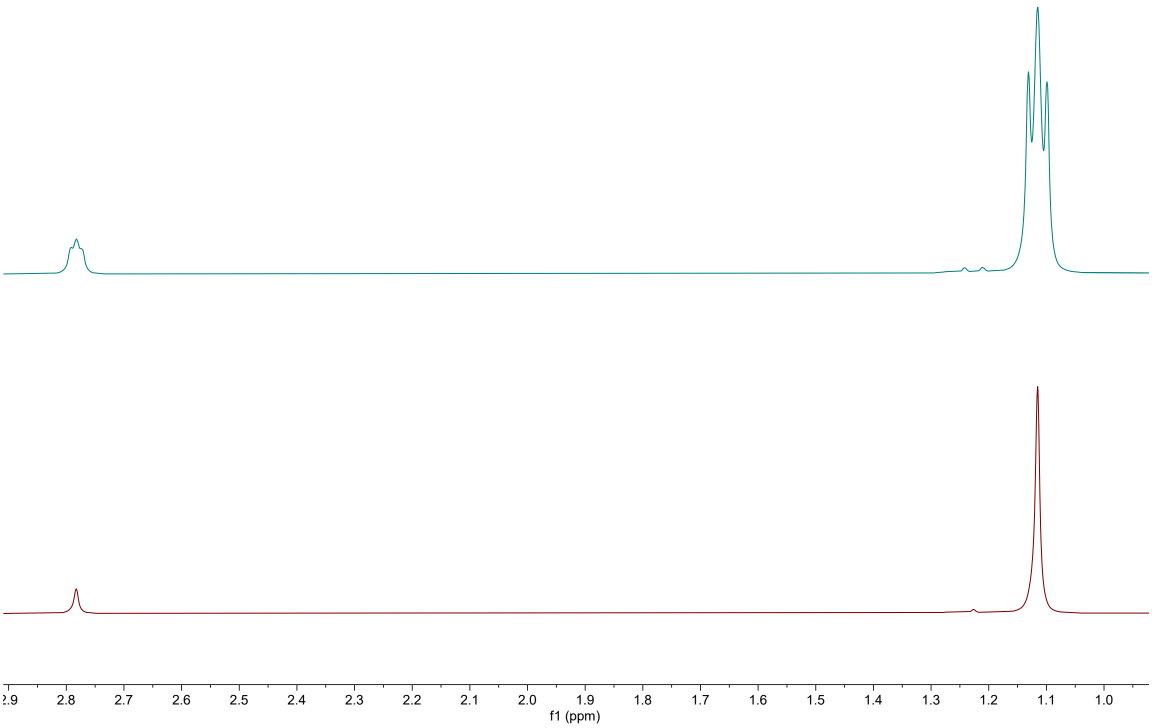

Figure S23: Section of the ^{1}H{^{31}P}-NMR spectrum of **4** in C_6D_6 at 25 °C, measured in a quartz NMR tube.

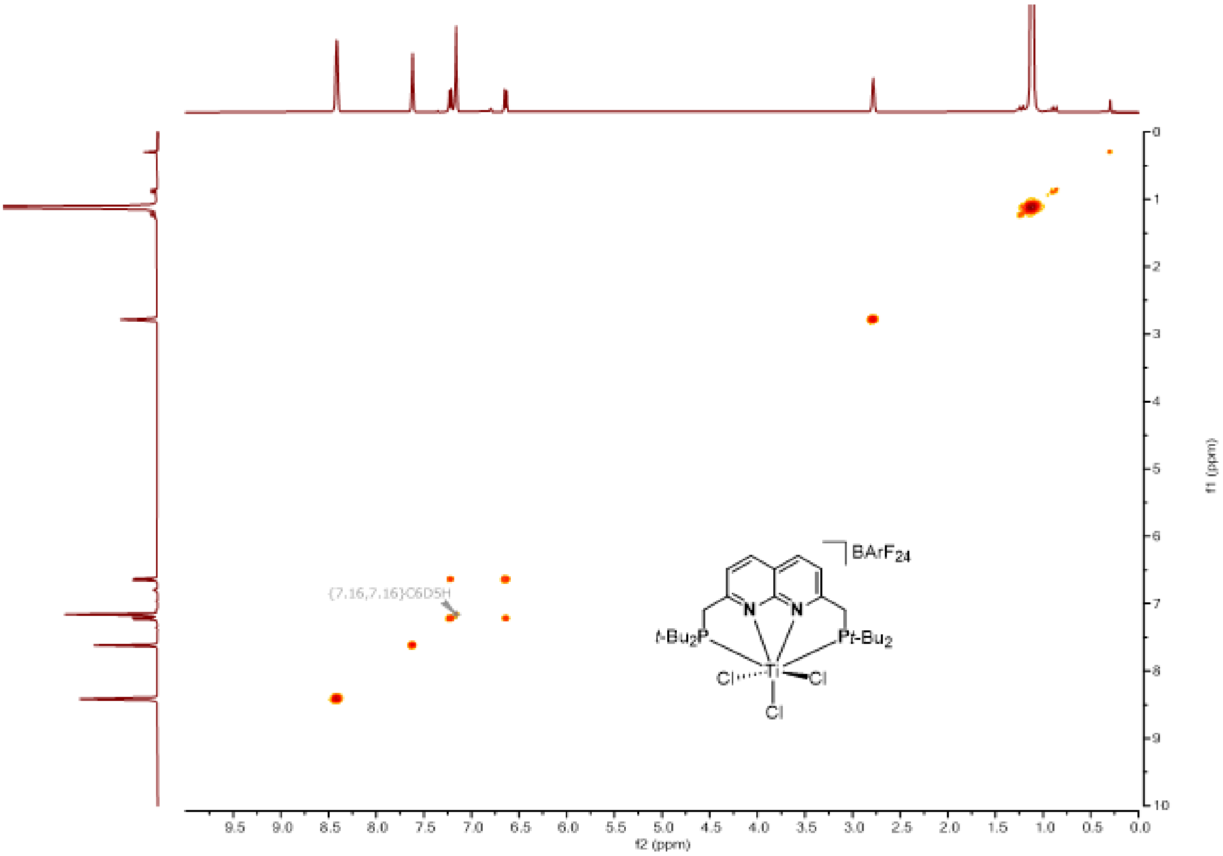

Figure S24: ^{1}H-COSY NMR spectrum of **4** in C_6D_6 at 25 °C, measured in a quartz NMR tube.

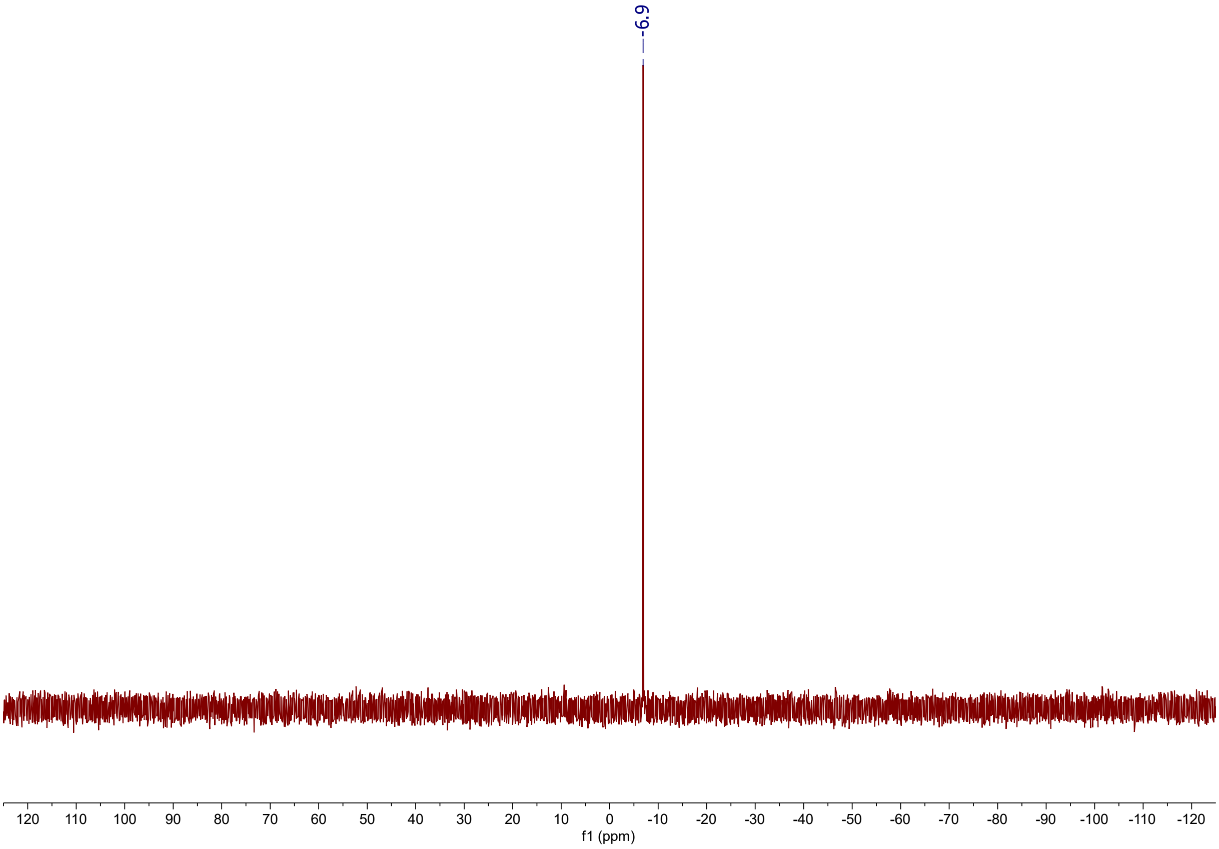

Figure S25: ^{11}B{^{1}H}-NMR spectrum of **4** in C_6D_6 at 25 °C, measured in a quartz NMR tube.

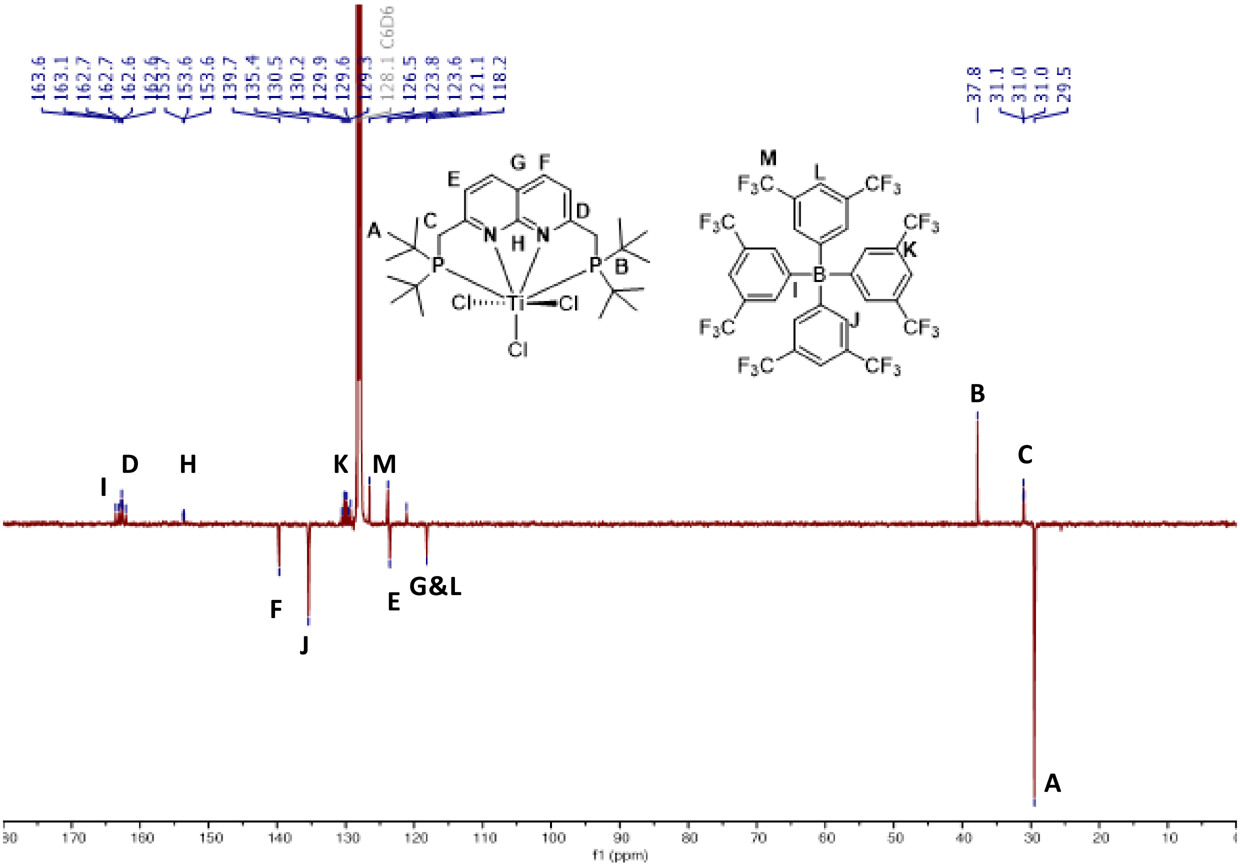

Figure S26: $^{13}C\{^{1}H\}$-APT NMR spectrum of **4** in C_6D_6 at 25 °C, measured in a quartz NMR tube.

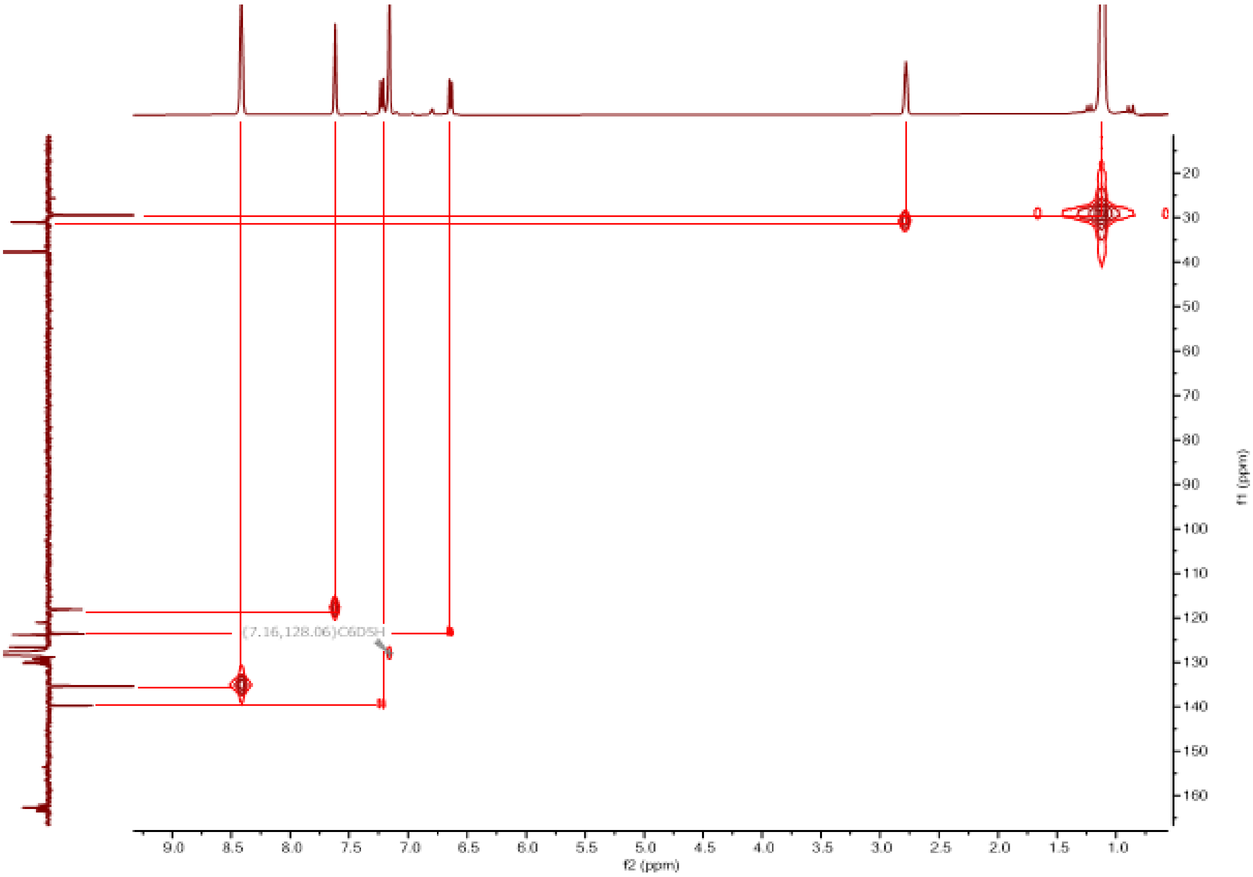

Figure S27: ^{1}H-^{13}C HMQC NMR spectrum of **4** in C_6D_6 at 25 °C, measured in a quartz NMR tube.

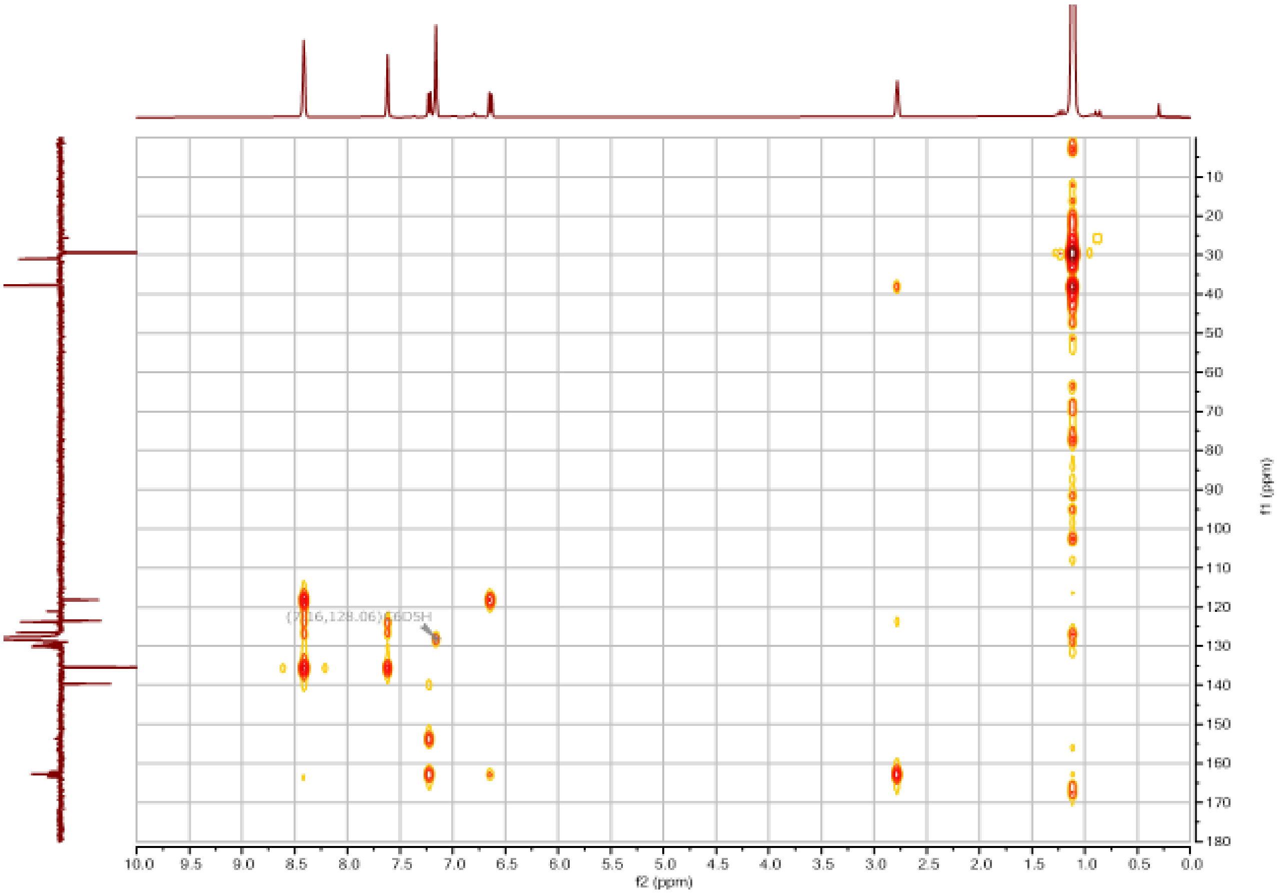

Figure S28: ^{1}H-^{13}C HMBC NMR spectrum of **4** in C_6D_6 at 25 °C, measured in a quartz NMR tube.

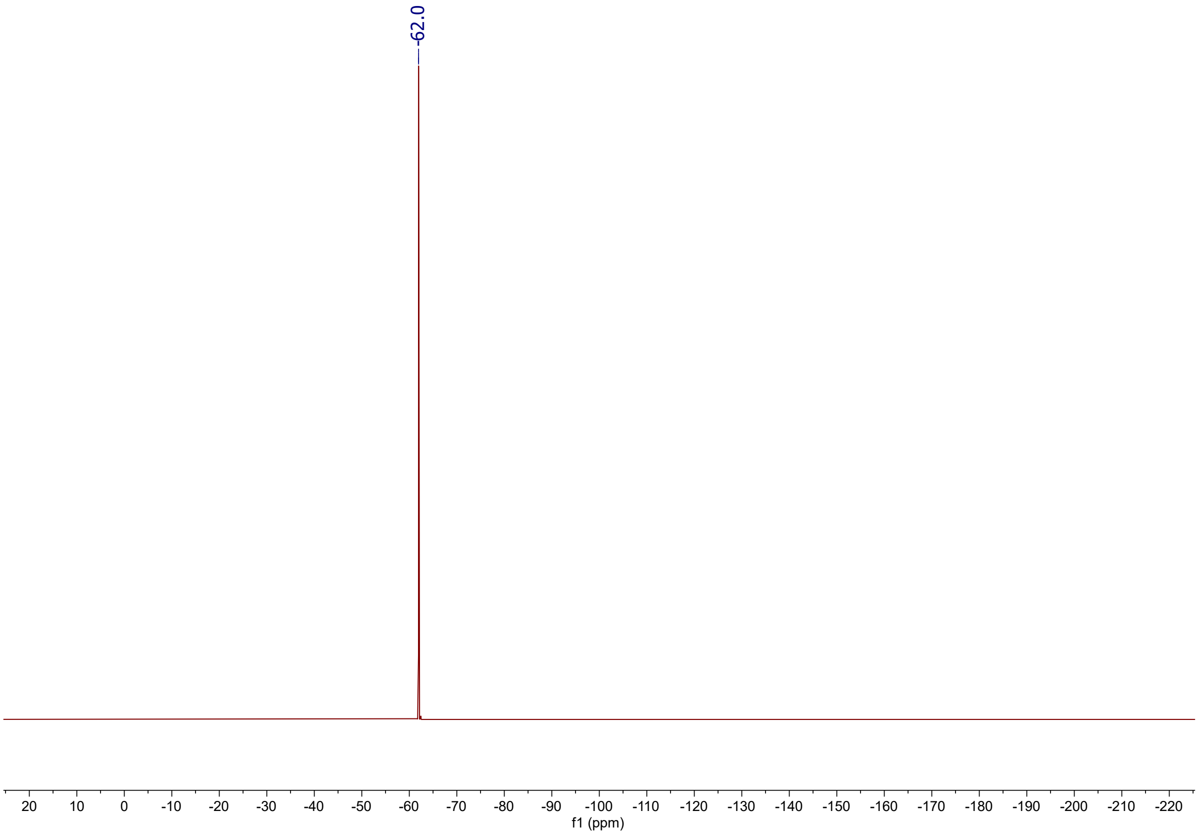

Figure S29: ^{19}F-NMR spectrum of **4** in C_6D_6 at 25 °C, measured in a quartz NMR tube.

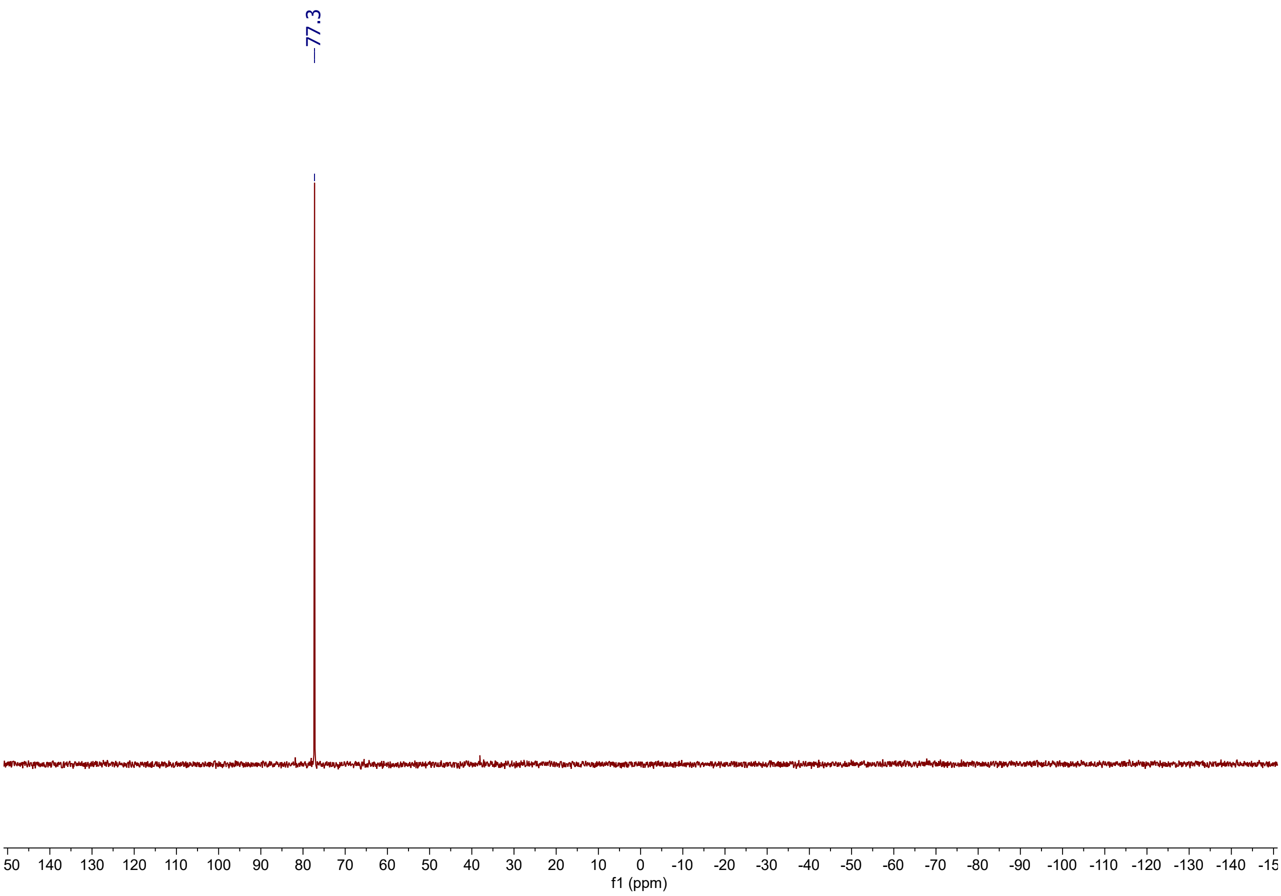

Figure S30: $^{31}P\{^{1}H\}$-NMR spectrum of **4** in C_6D_6 at 25 °C, measured in a quartz NMR tube.

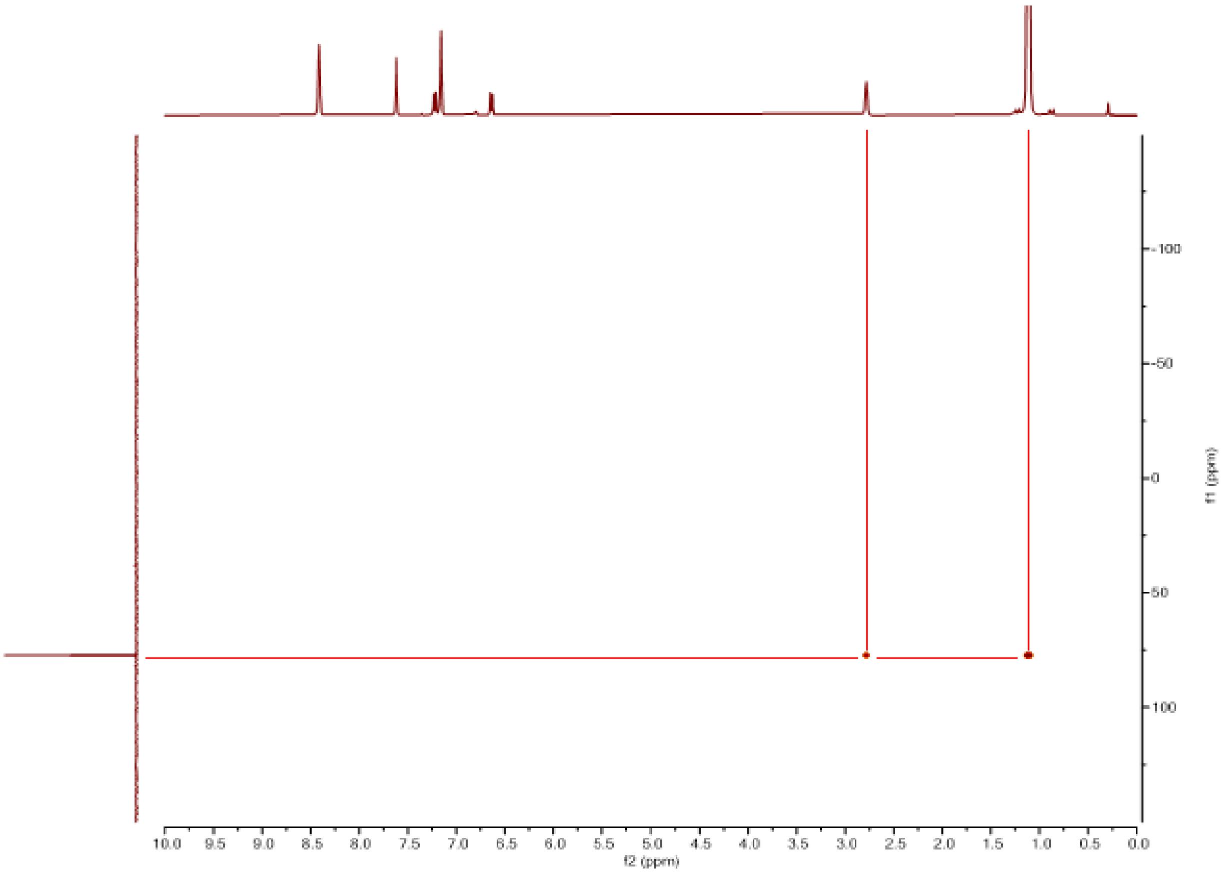

Figure S31: ^{1}H—P HMBC NMR spectrum of **4** in C_6D_6 at 25 °C, measured in a quartz NMR tube.

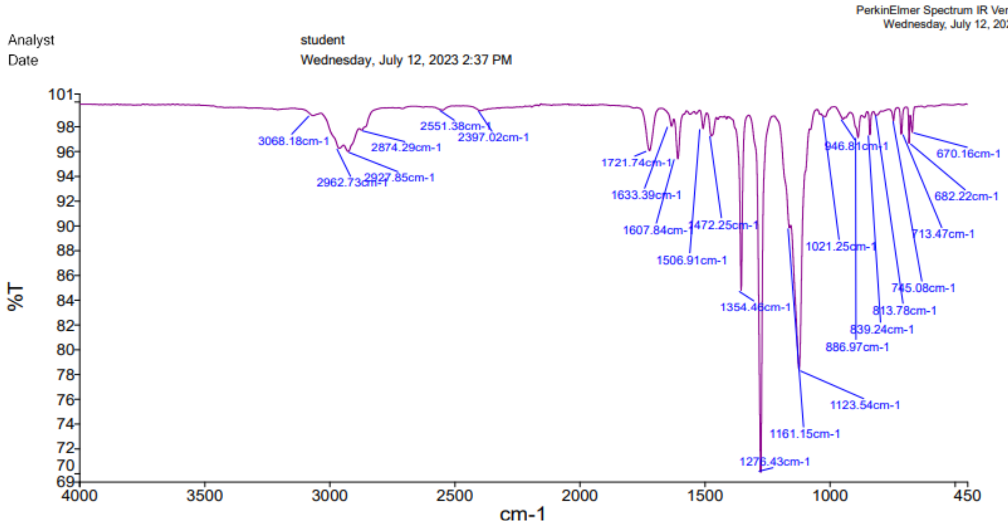

Figure S32: ATR-IR spectrum of **4** measured as a film under N_2 flow at 25 °C.

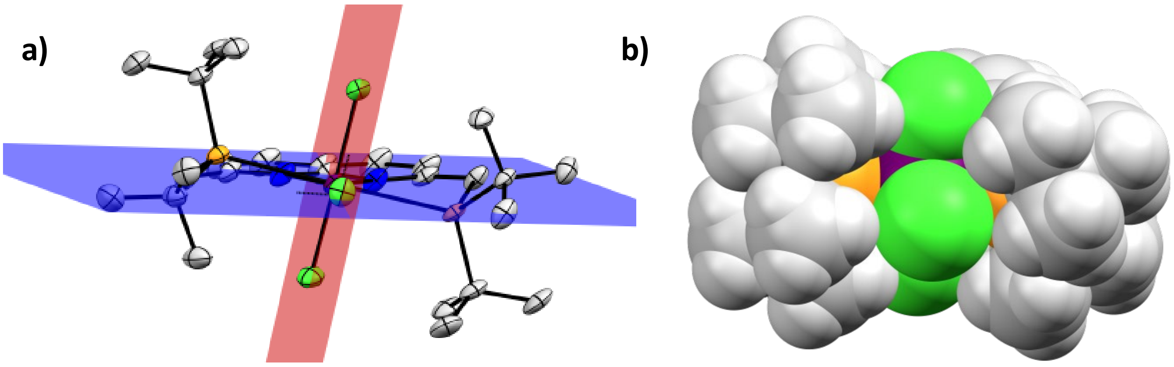

Figure S33: a) Dihedral angle between the $[TiCl_3]^+$ (red) and naphthyridine (blue) planes in the solid-state structure of **4** **b)** Space filling model of **4**.

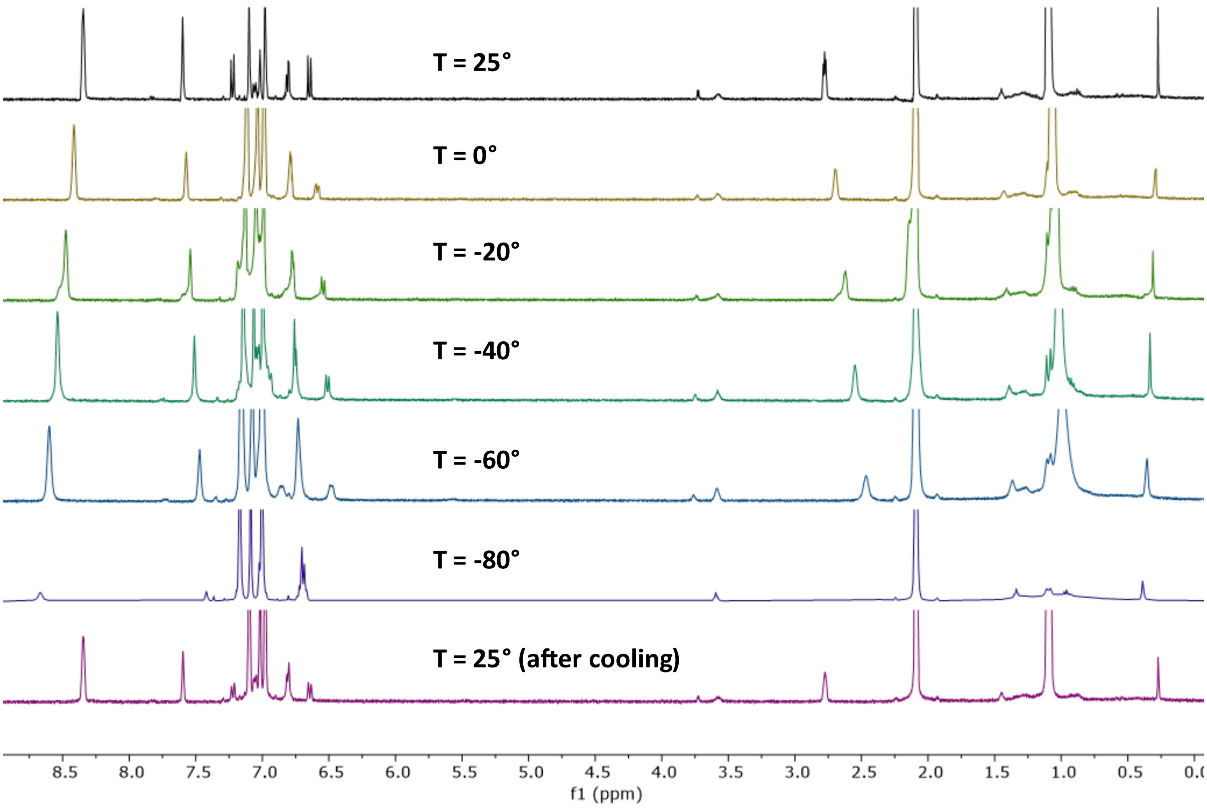

Figure S34: Variable temperature ^{1}H-NMR spectra of **4** in toluene-d_8.

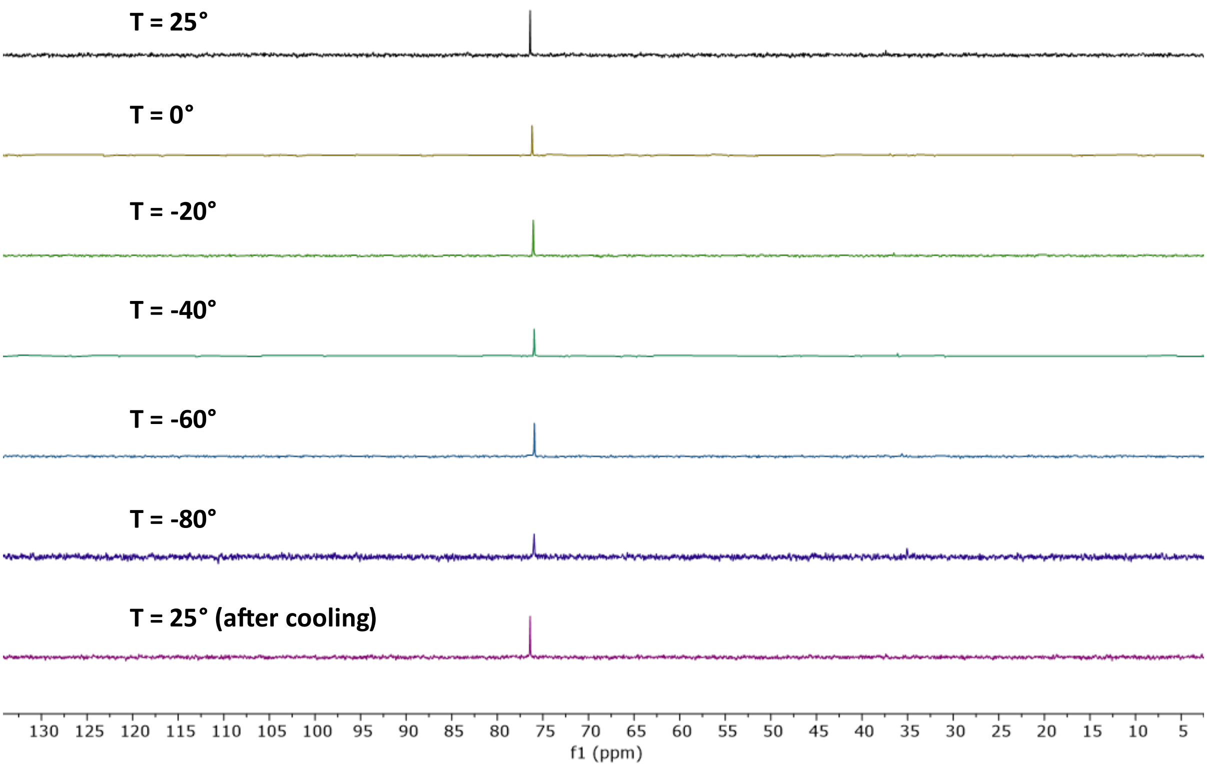

Figure S35: Variable temperature $^{31}P\{^{1}H\}$-NMR spectra of **4** in toluene-d_8.

1.6 Van der Waals-Corrected Bond Lengths of 4

Using the method described by Echeverría *et al.*[2] we obtained the penetration indices ($\boldsymbol{p}_{\mathrm{TiX}}$) for the various Ti-X bonds, using the metric data from the crystal structure of **4**. The empirical sets of covalent and Van der Waals radii used were obtained from the works of Cordero *et al.*[3] and Alvarez respectively.[4]

Table S1: Obtained penetration indices ($\mathbf{p}_{\mathrm{TiX}}$) of the various Ti-X bonds.

Bond	p_{TiX} (%)
**Ti-P1**	89
**Ti-P2**	89
**Ti-N1**	106
**Ti-N2**	106
**Ti-Cl1**	121
**Ti-Cl2**	122
**Ti-Cl3**	120

1.7 Synthesis of $^{t\text{-Bu}}$PNN^{Me}:

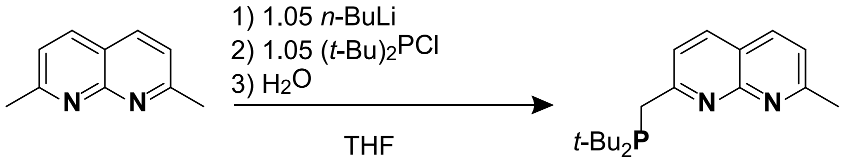

A Schlenk flask was charged with 2,7-dimethyl-1,8-naphthyridine (4.00 g, 25.3 mmol) and THF (100 mL). The mixture was then cooled to -78 °C in an acetone/dry ice bath and a solution of *n*-BuLi (1.6 M in hexanes, 17.6 mL, 26.6 mmol) was added dropwise over the course of 30 minutes. After the addition the red solution was allowed to warm to ambient temperature. Next, the solution was cannulated dropwise over the course of 20 minutes into a stirred solution of $P(^{t}Bu)_2Cl$ (5.3 mL, 27.8 mmol) in THF (30 mL) at -78 °C. After the addition the dark red mixture was allowed to warm to ambient temperature overnight and was quenched after 18 h by carefully adding degassed water (50 mL). After vigorous stirring for 1 h, the orange mixture was extracted with DCM (3x30 mL). The extracts were combined, dried over Na_2SO_4 and concentrated in vacuum to yield an orange solid, which was transferred into a N_2-filled glovebox. The solid was extracted by vigorous stirring with n-hexane (30 mL, 20 min), Et_2O (30 mL, 20 min) and THF (30 mL, 10 min). Next, the off-white solid was suspended in 15 mL of benzene and stirred overnight. Subsequent filtration and drying in vacuum gave an off-white solid (2.52 g, 33%).

^{1}H-NMR (400 MHz, CD_2Cl_2, 298 K): δ 8.03 (d, $^{3}J_{H,H}$ = 2.8 Hz, 1H), 8.01 (d, $^{3}J_{H,H}$ = 2.8 Hz, 1H), 7.59 (d, $^{3}J_{H,H}$ = 8.3 Hz, 1H), 7.30 (d, $^{3}J_{H,H}$ = 8.3 Hz, 1H), 3.26 (d, $^{2}J_{H,p}$ = 3.5 Hz, 2H), 2.74 (s, 3H), 1.17 (d, $^{3}J_{H,P}$ = 11.1 Hz, 18H).

^{13}C{^{1}H}-NMR (101 MHz, CD_2Cl_2, 298 K): δ = 166.8 (d, $^{2}J_{P,C}$ = 14.5 Hz), 162.7 (s), 155.9 (s), 136.9 (s), 136.5 (s), 123.0 (d, $^{3}J_{P,C}$ = 8.8 Hz), 122.3 (s), 119.1 (d, $^{5}J_{P,C}$ = 1.2 Hz), 33.7 (d, $^{1}J_{P,C}$ = 26.3 Hz), 32.4 (d, $^{1}J_{P,C}$ = 22.9 Hz), 29.9 (d, $^{2}J_{P,C}$ = 13.6 Hz), 25.8 (s).

^{31}P{^{1}H}-NMR (162 MHz, CD_2Cl_2, 298 K): δ = 36.0 (s).

IR-ATR (cm^{-1}): 2944 (s), 2891 (m), 2859 (s), 1604 (s), 1541 (m), 1505 (s), 1459 (m), 1442 (m), 1364 (m), 1310 (w), 1242 (m), 1147 (w), 852 (m), 830 (w), 802 (w), 781 (m), 500 (w).

In lieu of Elemental analysis, we measured the spectroscopic purity of the compound at >98%.

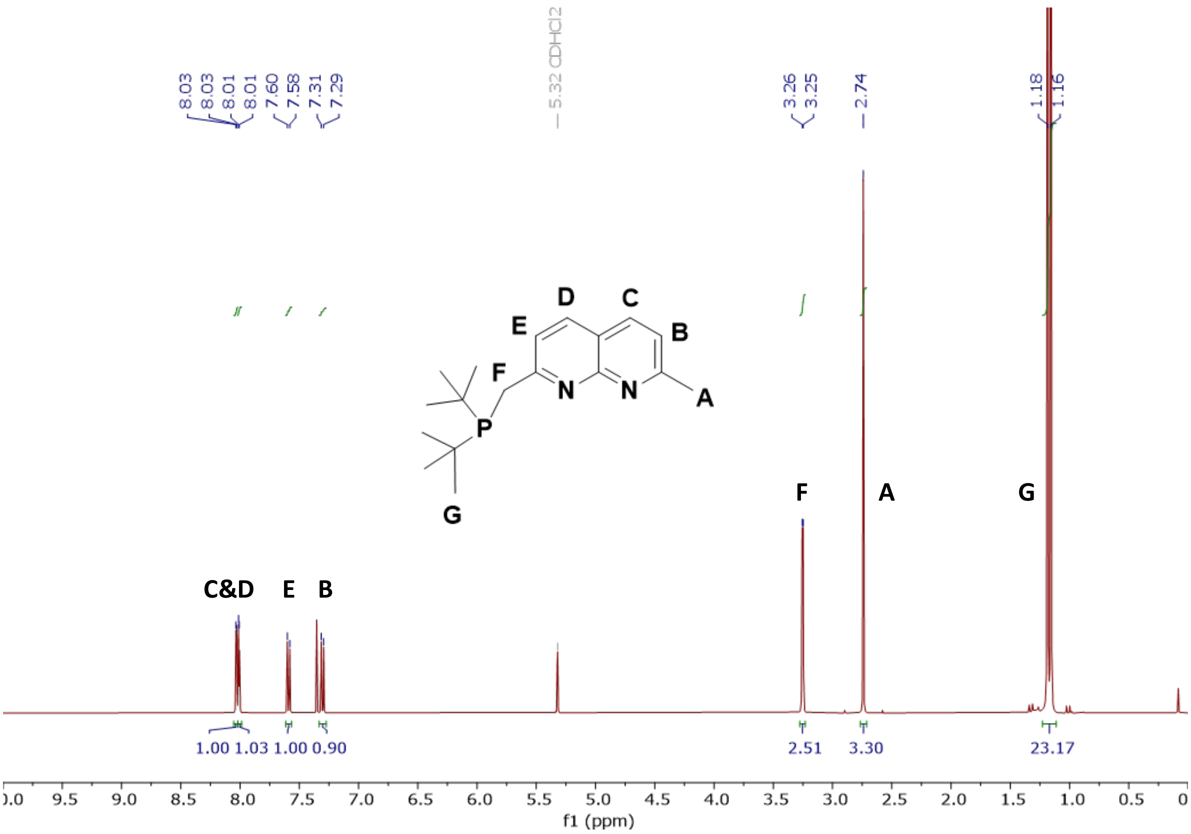

Figure S36: ^{1}H-NMR spectrum of $^{t\text{-Bu}}$**PNN**^{Me} in CD_2Cl_2 at 25 °C.

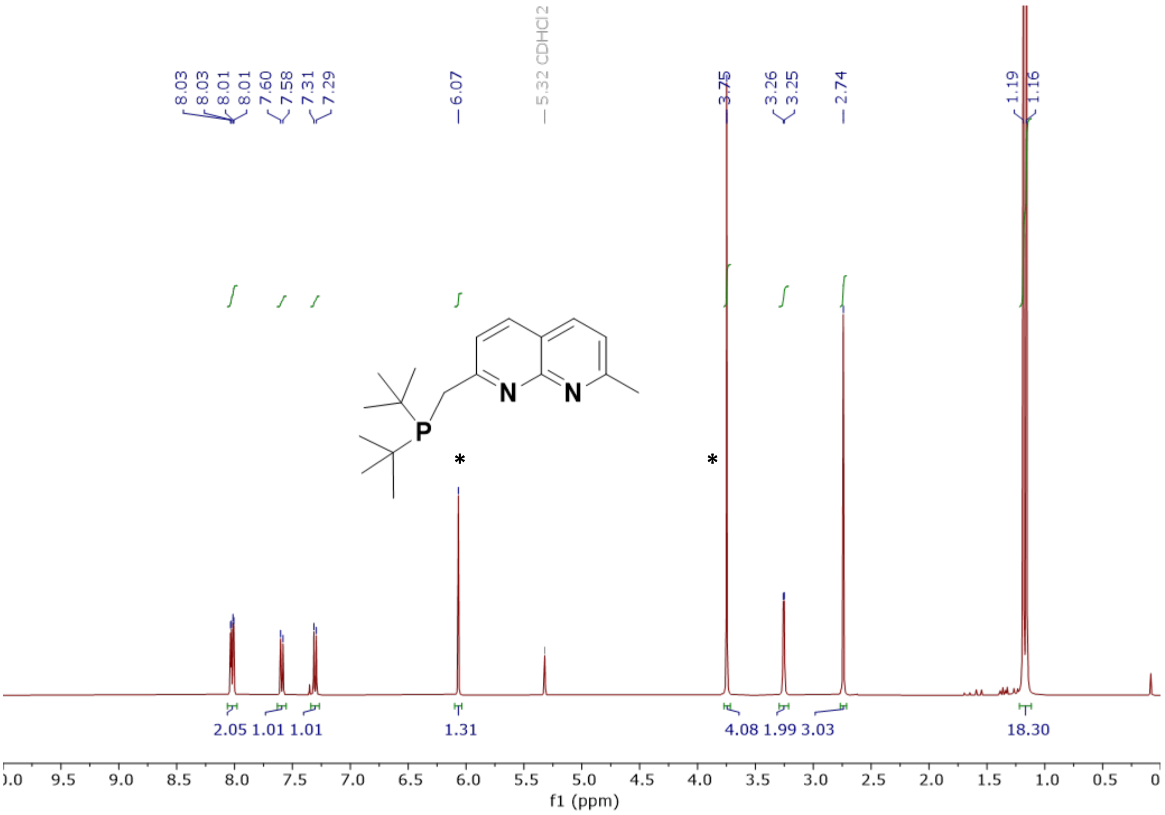

Figure S37: Quantitative (30 sec relaxation delay) ^{1}H-NMR spectrum of 10.0 mg $^{t\text{-Bu}}$**PNN**^{Me} and 2.5 mg trimethoxybenzene in CD_2Cl_2 at 25 °C. The resonances marked with a * are those of trimethoxybenzene.

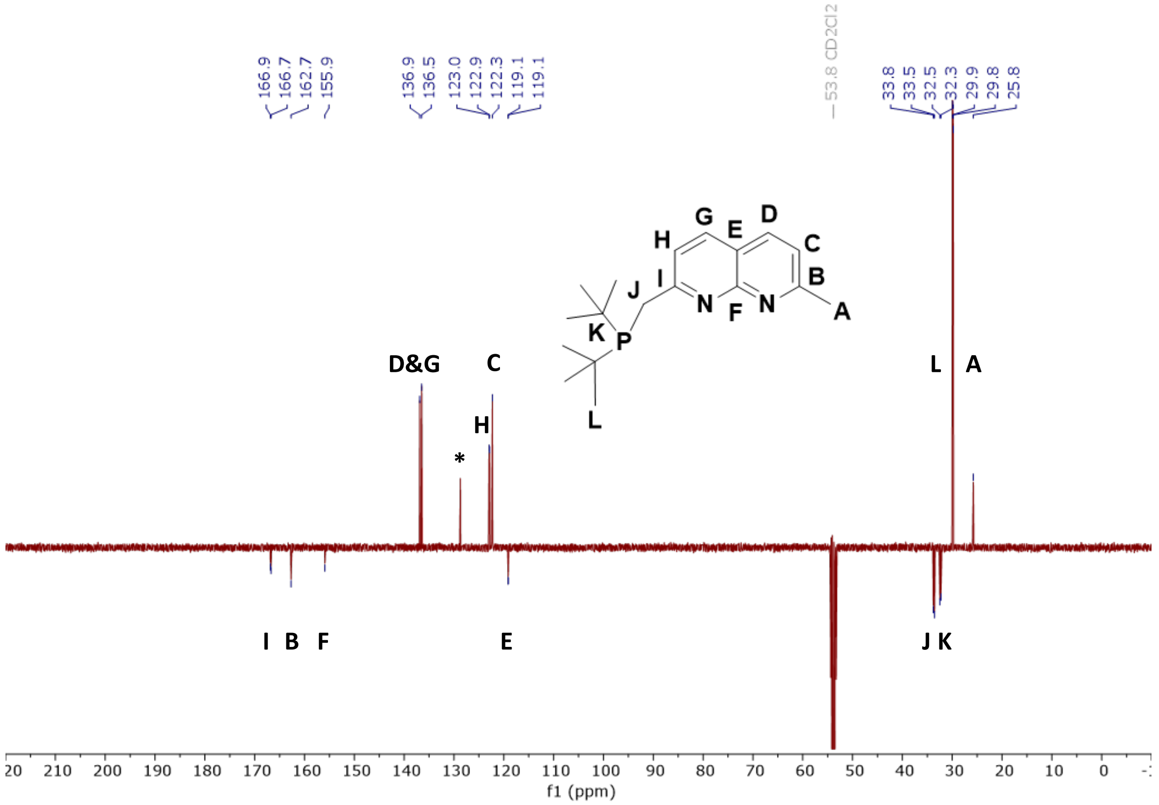

Figure S38: $^{13}C\{^{1}H\}$-APT NMR spectrum of $^{t\text{-Bu}}$**PNN**^{Me} in CD_2Cl_2 at 25 °C.

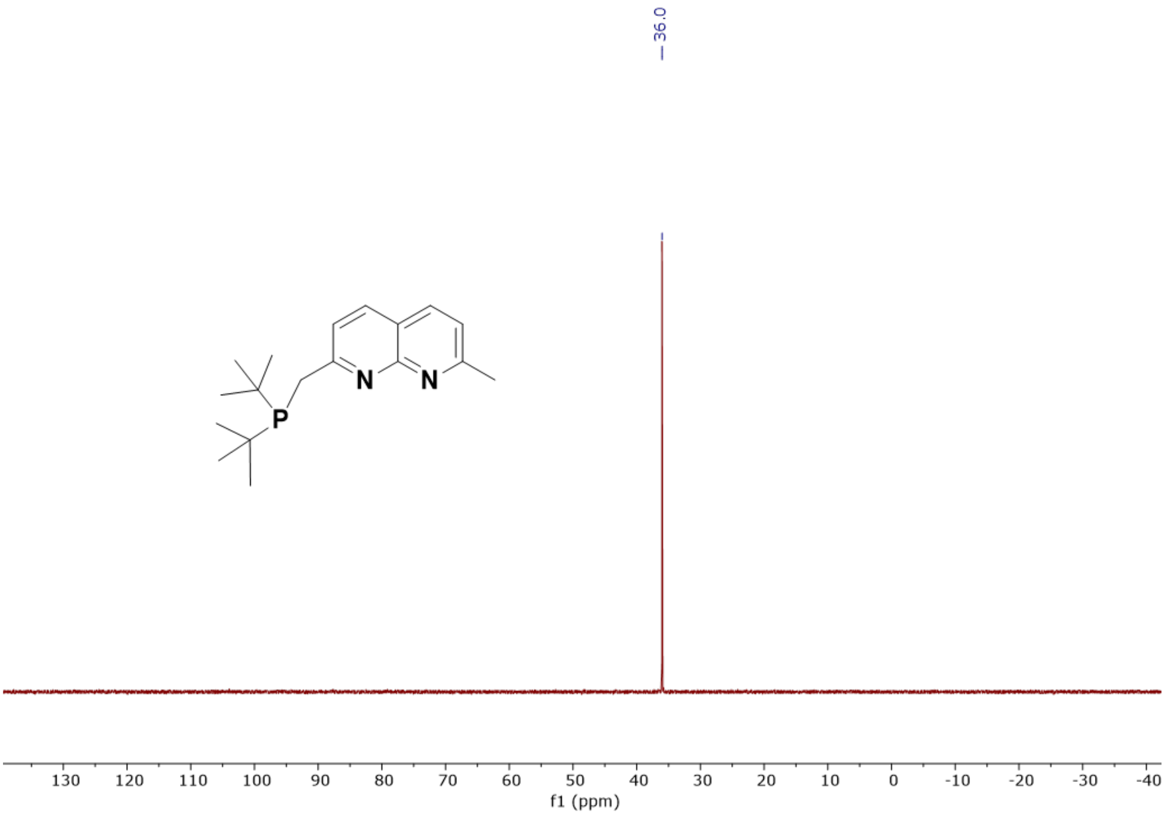

Figure S39: $^{31}P\{^{1}H\}$-NMR spectrum of $^{t\text{-Bu}}$**PNN**^{Me} in CD_2Cl_2 at 25 °C.

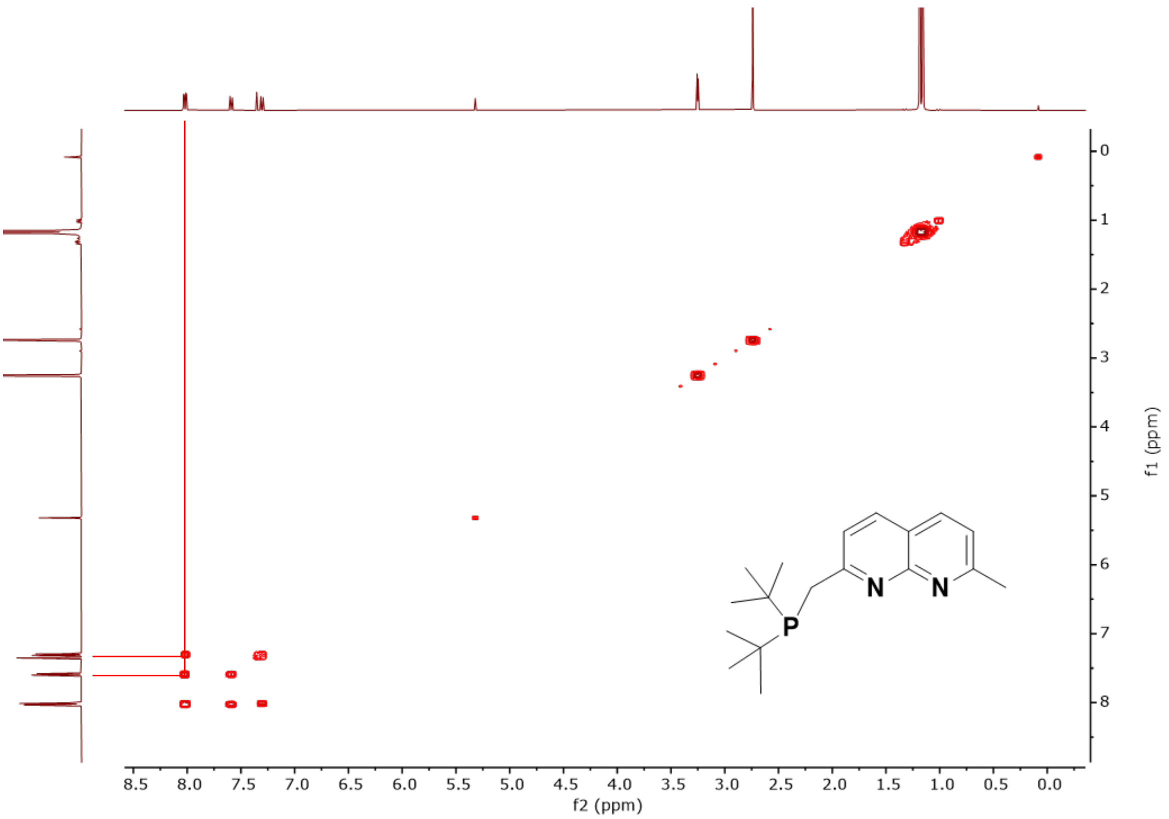

Figure S40: The ^{1}H-COSY NMR spectrum of ***t*-BuPNNMe** in CD_2Cl_2 at 25 °C.

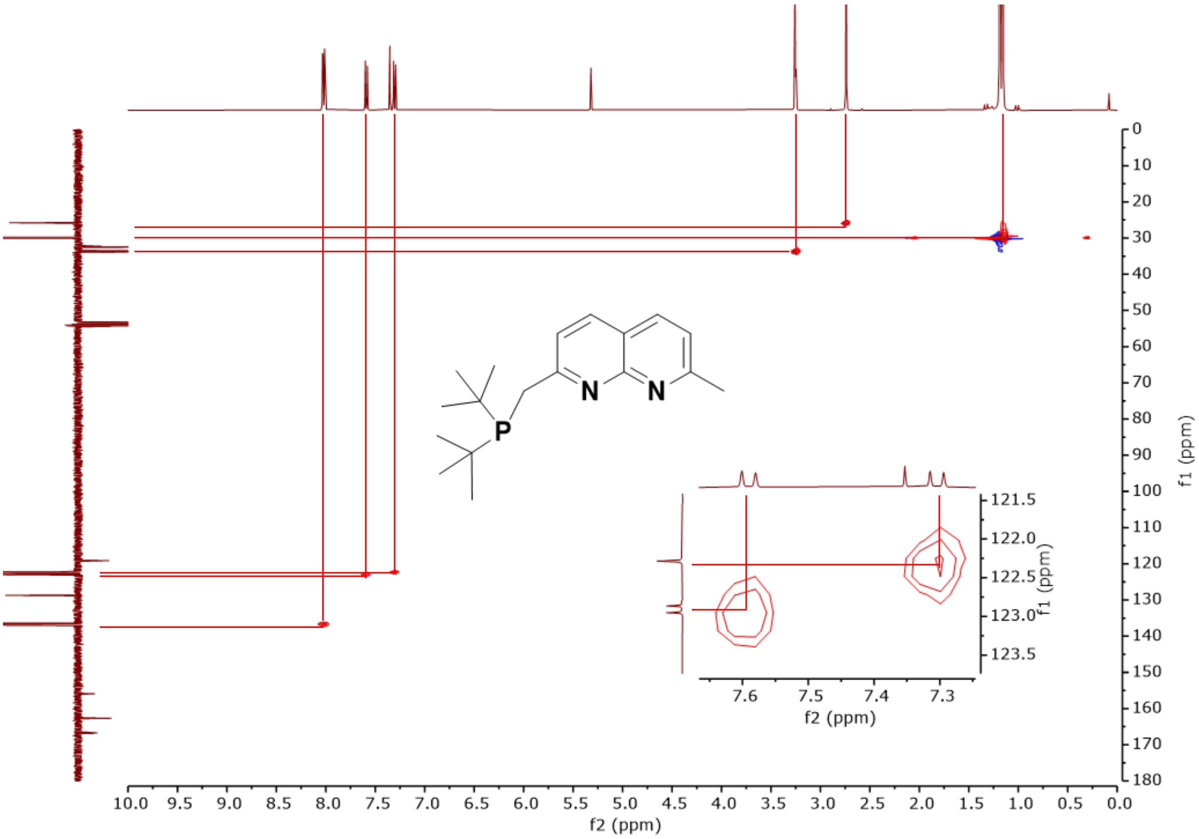

Figure S41: The ^{1}H-^{13}C HMQC NMR spectrum of ***t*-BuPNNMe** in CD_2Cl_2 at 25 °C.

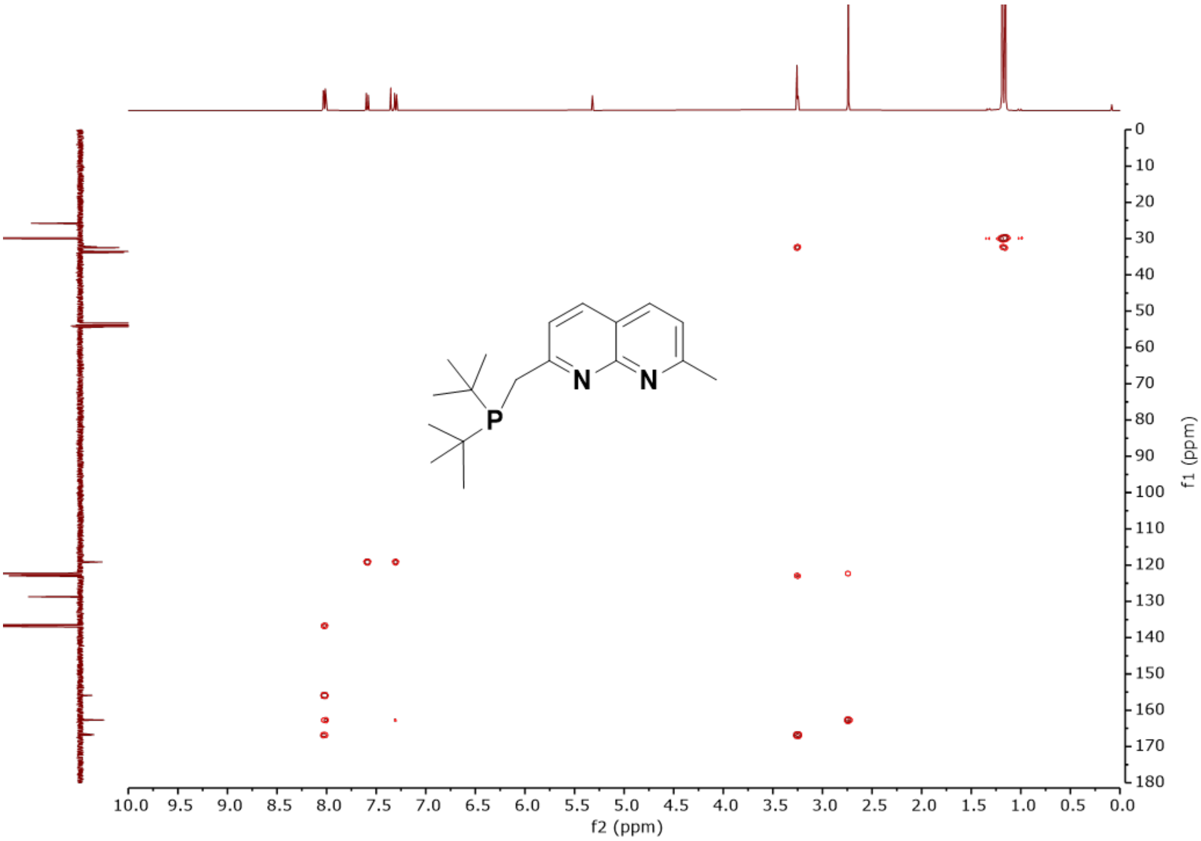

Figure S42: ^{1}H-^{13}C HMBC NMR spectrum of ***t*-BuPNNMe** in CD_2Cl_2 at 25 °C.

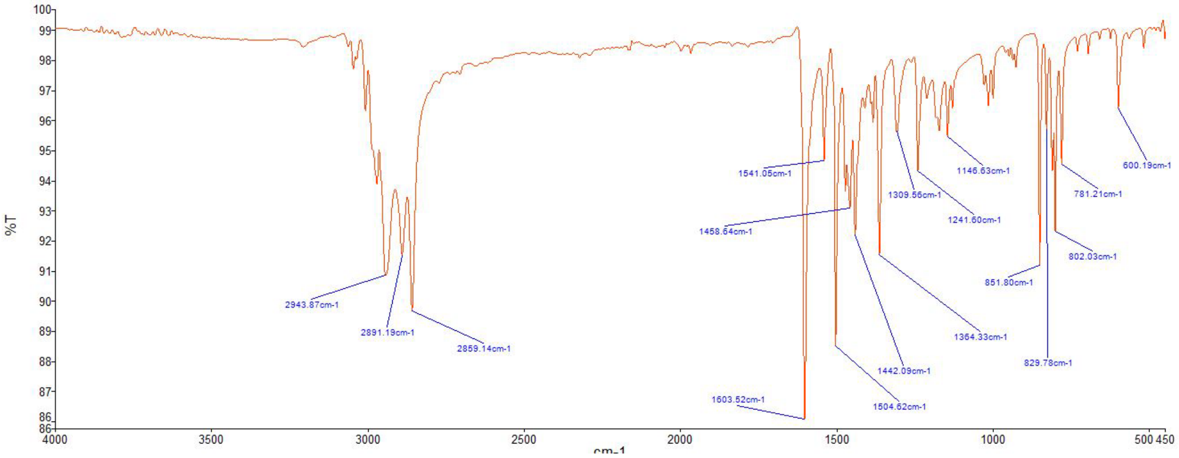

Figure S43: ATR-IR spectrum of complex ***t*-BuPNN** measured as a film under N_2 flow at 25 °C.

1.8 Synthesis of $^{t\text{-Bu}}$PNN^{Me}TiCl$_4$:

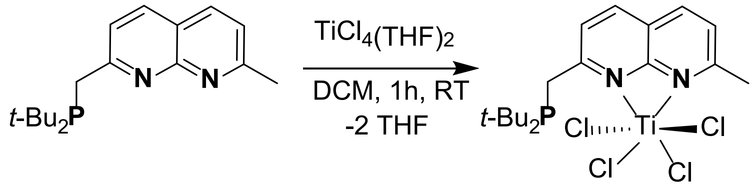

A solution of $TiCl_4(THF)_2$ (55.3 mg, 165.3 µmol) in CH_2Cl_2 (4 mL) was added dropwise to a stirring solution of $^{t\text{-Bu}}$PNN^{Me} (50.0 mg, 165.3 µmol) in CH_2Cl_2 (4 mL) at ambient temperature, resulting in a solution progressively turning darker orange. After 1 h the slightly cloudy mixture was filtered and the volatiles were removed under vacuum giving an orange powder (78.4 mg, 96%).

^{1}H-NMR (400 MHz, CD_2Cl_2, 298 K): δ 8.39* (d, $^3J_{H,H}$ = 8.4 Hz, 1H), 8.38* (d, $^3J_{H,H}$ = 8.6 Hz, 1H),), 8.28 (d, $^3J_{H,H}$ = 8.6 Hz, 1H), 7.64 (d, $^3J_{H,H}$ = 8.4 Hz, 1H), 3.67 (s, broad, 2H), 3.06 (s, broad, 3H), 1.20 (d, $^3J_{H,P}$ = 11.5 Hz, 18H).

^{13}C{^{1}H}-NMR (101 MHz, CD_2Cl_2, 298 K): δ 167.2 (d, $^2J_{C,P}$ = 15.1 Hz), 162.0, 154.2, 139.3, 138.1, 126.6 (d, $^4J_{C,P}$ = 18.0 Hz), 126.1, 118.7, 32.7 (d, $^1J_{C,P}$ = 21.3 Hz), 31.3 (d, $^1J_{C,P}$ = 26.8 Hz), 29.8 (d, $^2J_{C,P}$ = 13.6 Hz), 24.4.

^{31}P{^{1}H}-NMR (162 MHz, CD_2Cl_2, 298 K): δ 39.2 (s).

*Overlapping resonances

IR-ATR (cm^{-1}): 3066 (w), 2944 (s), 2896 (m), 2863 (m), 1605 (s), 1562 (m), 1508 (s), 1469 (m), 1435 (m), 1389 (m), 1367 (m), 1310 (m), 1252 (m), 1223 (w), 1176 (w), 1150 (m), 1017 (w),856 (m), 815 (m), 797 (m), 736 (m), 700 (w), 644 (w), 593 (w), 462 (w).

The reactive nature of the compound precluded obtaining a satisfactory result for Elemental analysis.

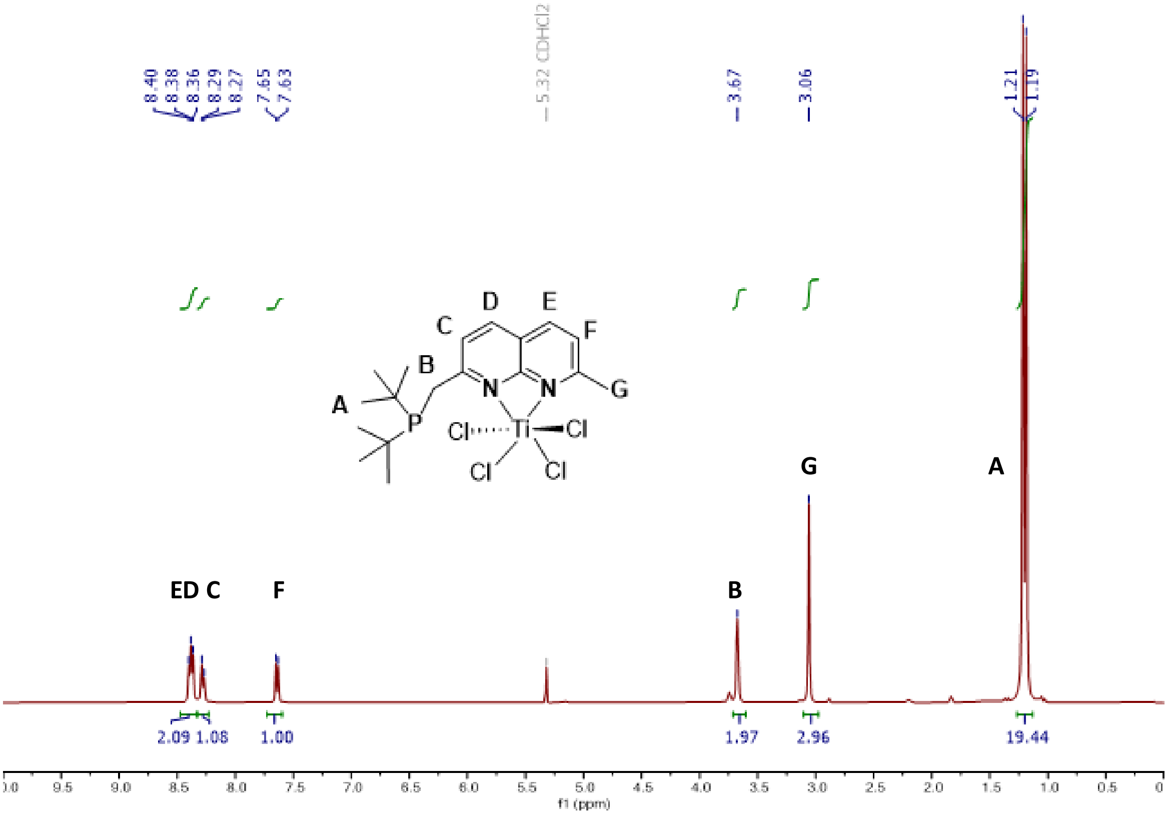

Figure S44: ^{1}H-NMR spectrum of $^{t\text{-Bu}}$**PNN^{Me}TiCl$_4$** in CD_2Cl_2 at 25 °C.

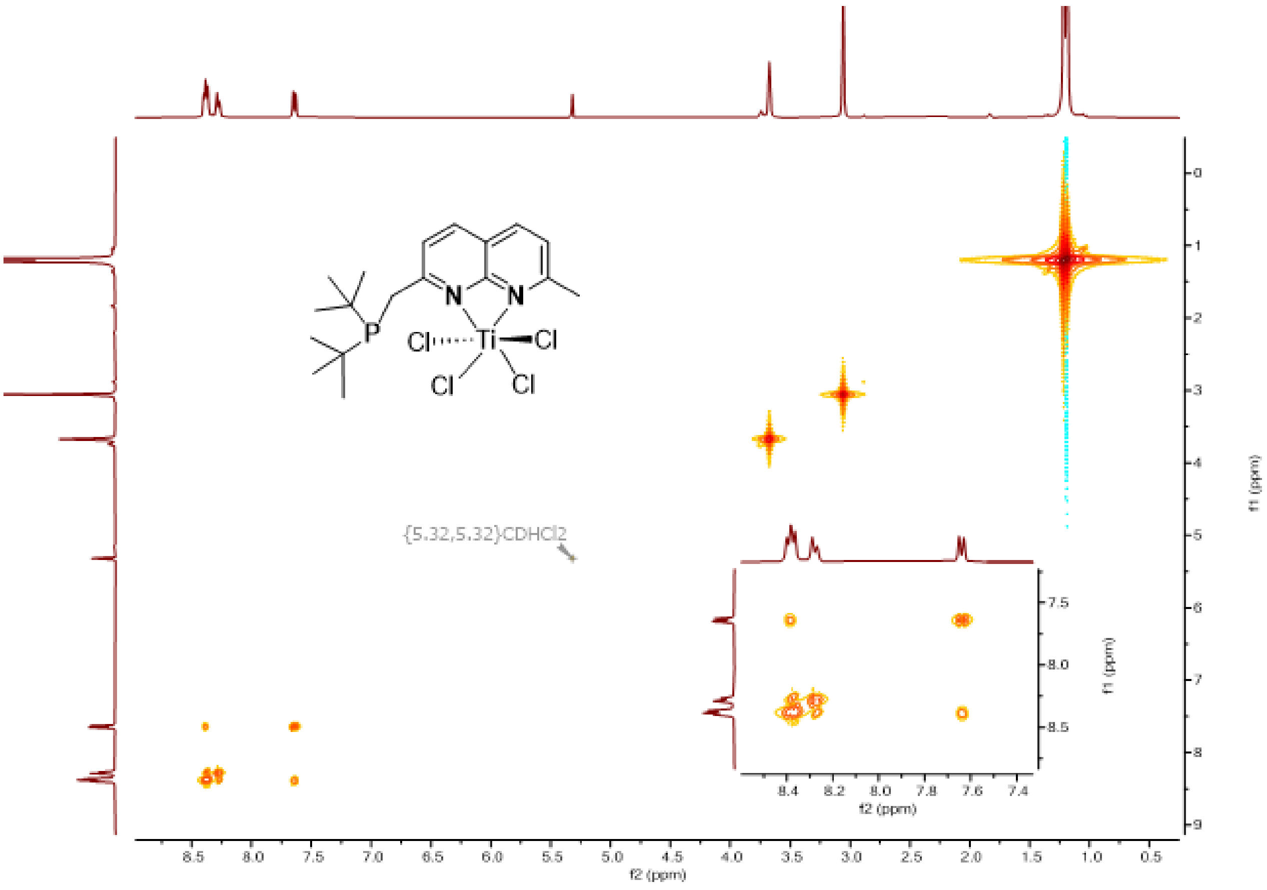

Figure S45: ^{1}H-NMR spectrum of $^{t\text{-Bu}}$**PNN^{Me}TiCl$_4$** in CD_2Cl_2 at 25 °C, inset shows a zoom-in on the aromatic resonances.

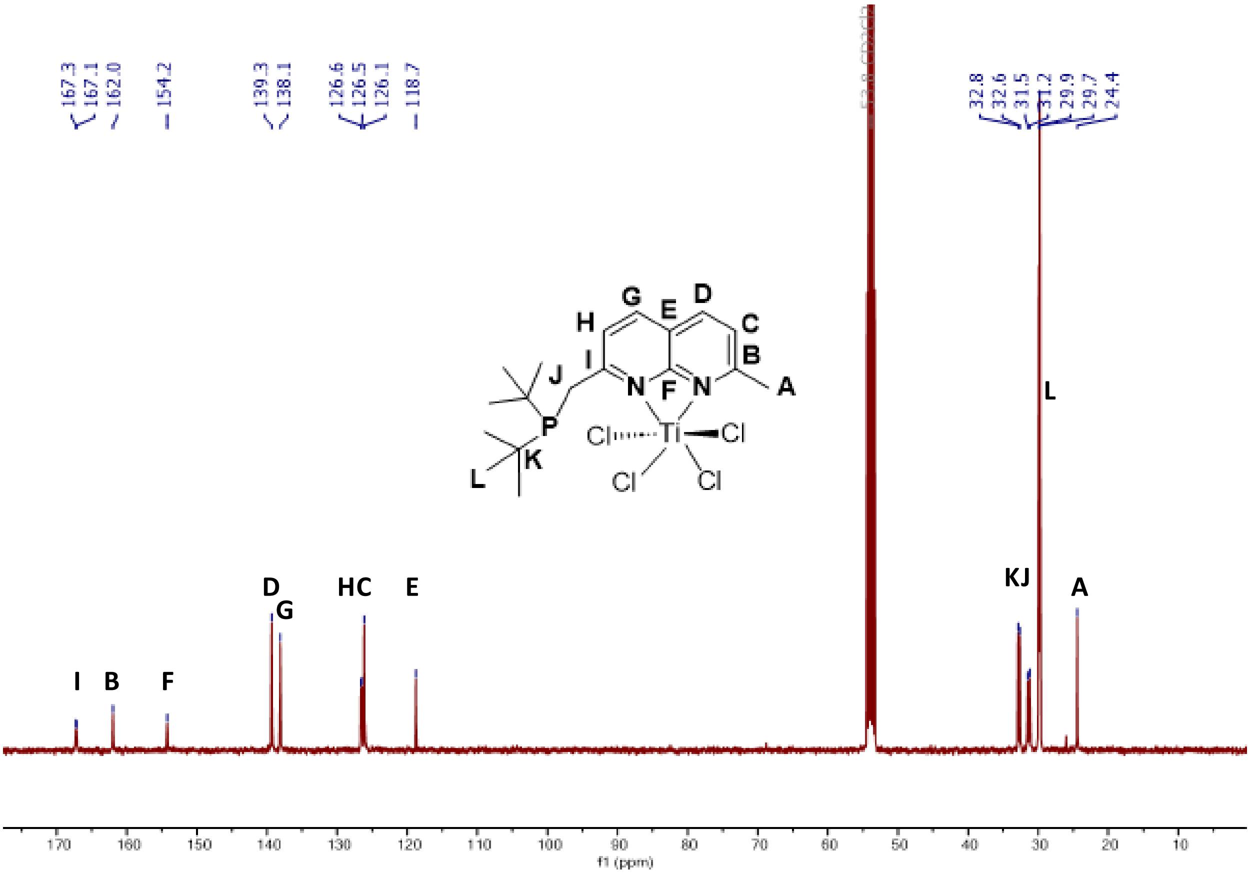

Figure S46: The $^{13}C\{^{1}H\}$-NMR spectrum of **$^{t\text{-Bu}}PNN^{Me}TiCl_4$** in CD_2Cl_2 at 25 °C.

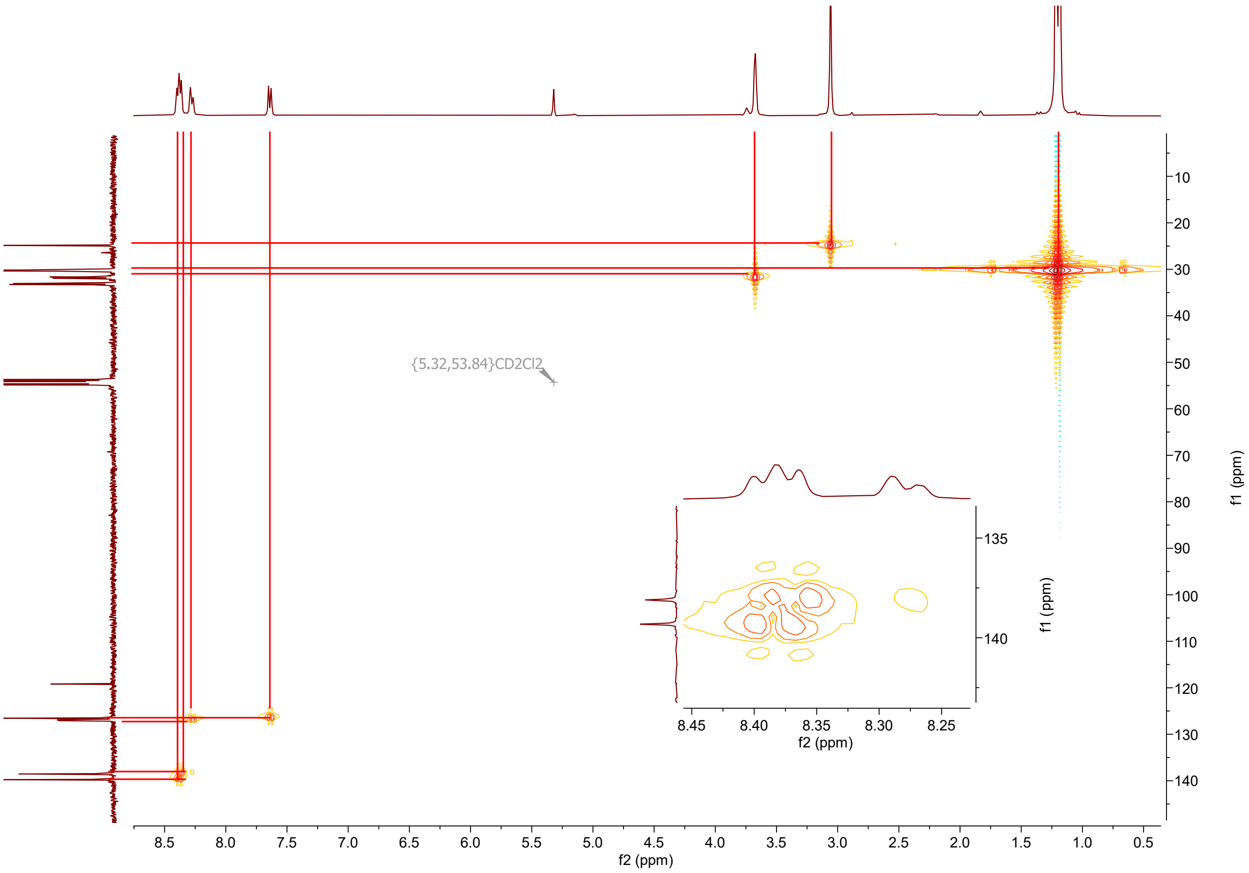

Figure S47: The ^{1}H-^{13}C HMQC NMR spectrum of **$^{t\text{-Bu}}PNN^{Me}TiCl_4$** in CD_2Cl_2 at 25 °C.

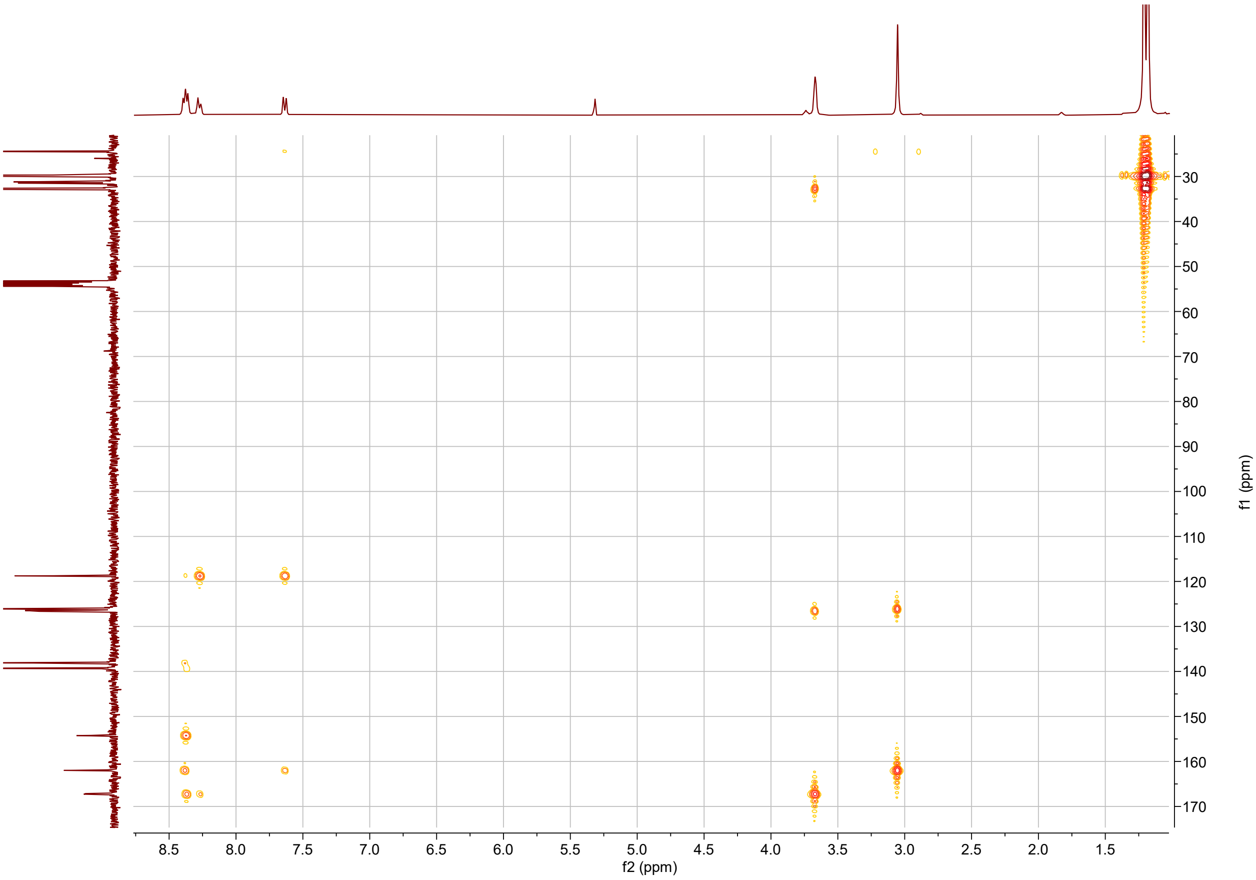

Figure S48: ^{1}H-^{13}C HMBC NMR spectrum of ***t*-BuPNNMeTiCl4** ($^{t\text{-}Bu}PNN^{Me}TiCl_4$) in CD_2Cl_2 at 25 °C.

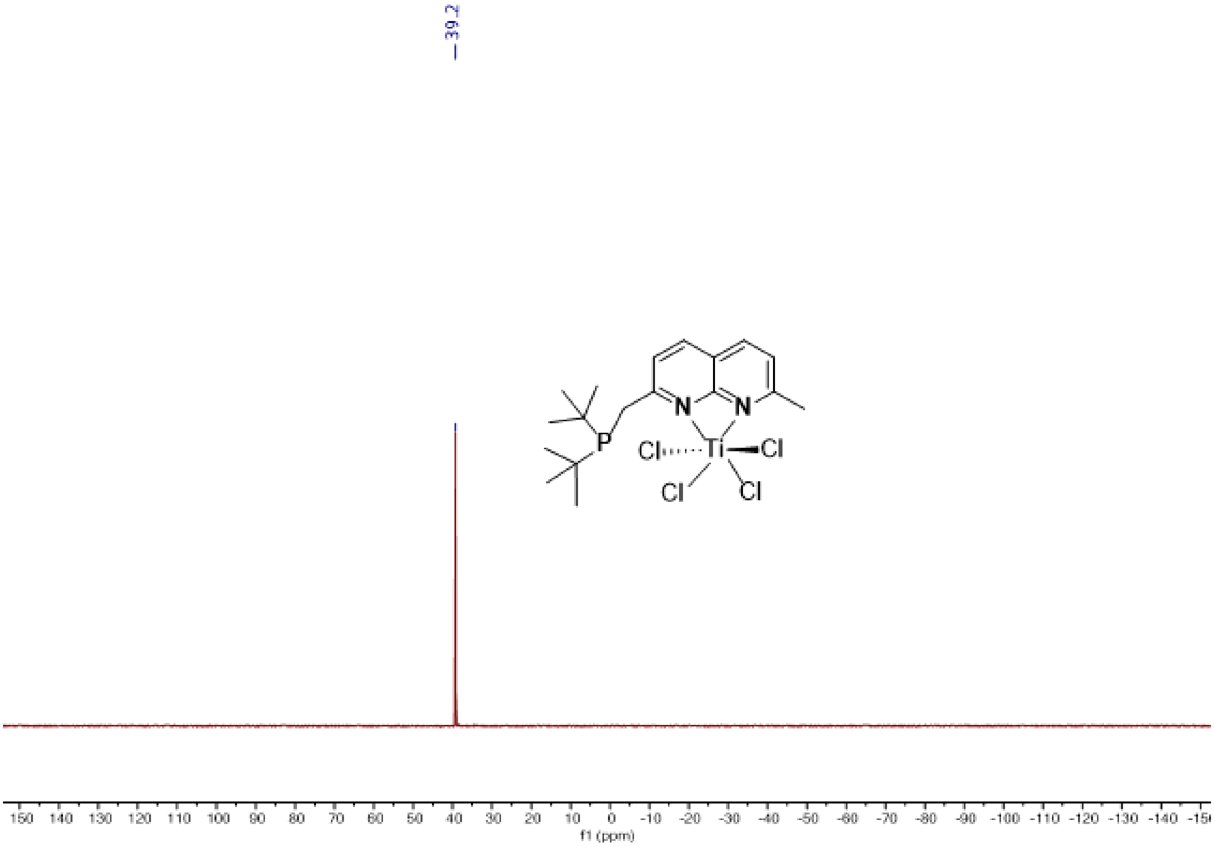

Figure S49: $^{31}P\{^{1}H\}$-NMR spectrum of ***t*-BuPNNMeTiCl4** ($^{t\text{-}Bu}PNN^{Me}TiCl_4$) in CD_2Cl_2 at 25 °C.

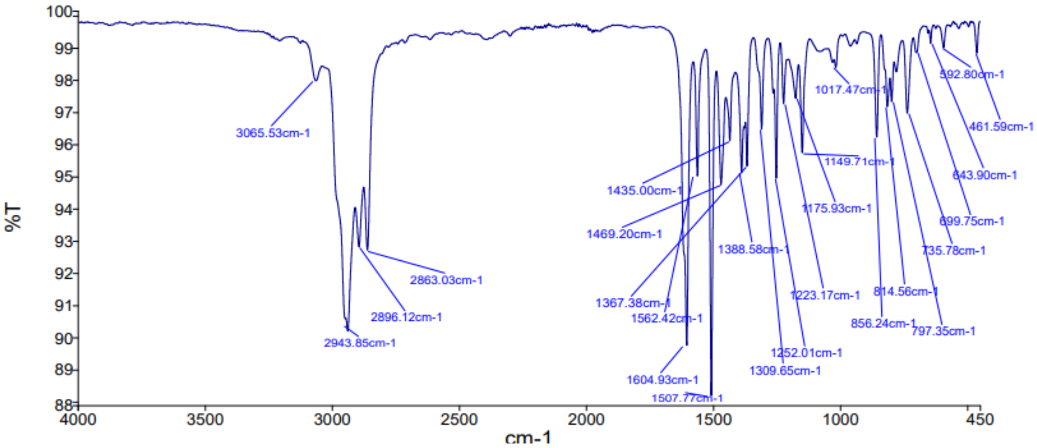

Figure S50: The ATR-IR spectrum of complex ***t*-BuPNNTiCl$_4$** measured as a film under N_2 flow at 25 °C.

1.9 Halide abstraction from $^{t\text{-Bu}}$PNN^{Me}TiCl$_4$:

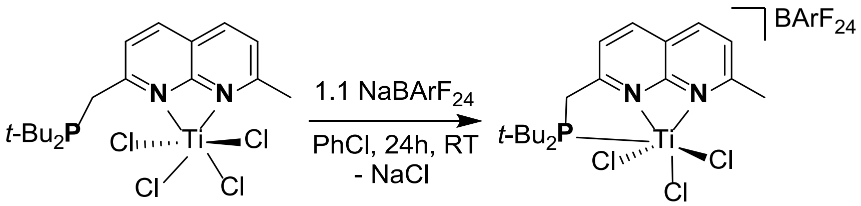

A suspension of NaBArF$_{24}$ (19.8 mg, 22.4 µmol) in PhCl (3 mL) was added dropwise to a stirring solution of $^{t\text{-Bu}}$**PNN^{Me}TiCl$_4$** (10.0 mg, 20.4 µmol) in PhCl (1 mL). The colour turned darker over the course of 24 h at ambient temperature. Afterwards, the mixture was filtered and concentrated *in vacuo* to 25.6 mg. of a dark yellow/green film.

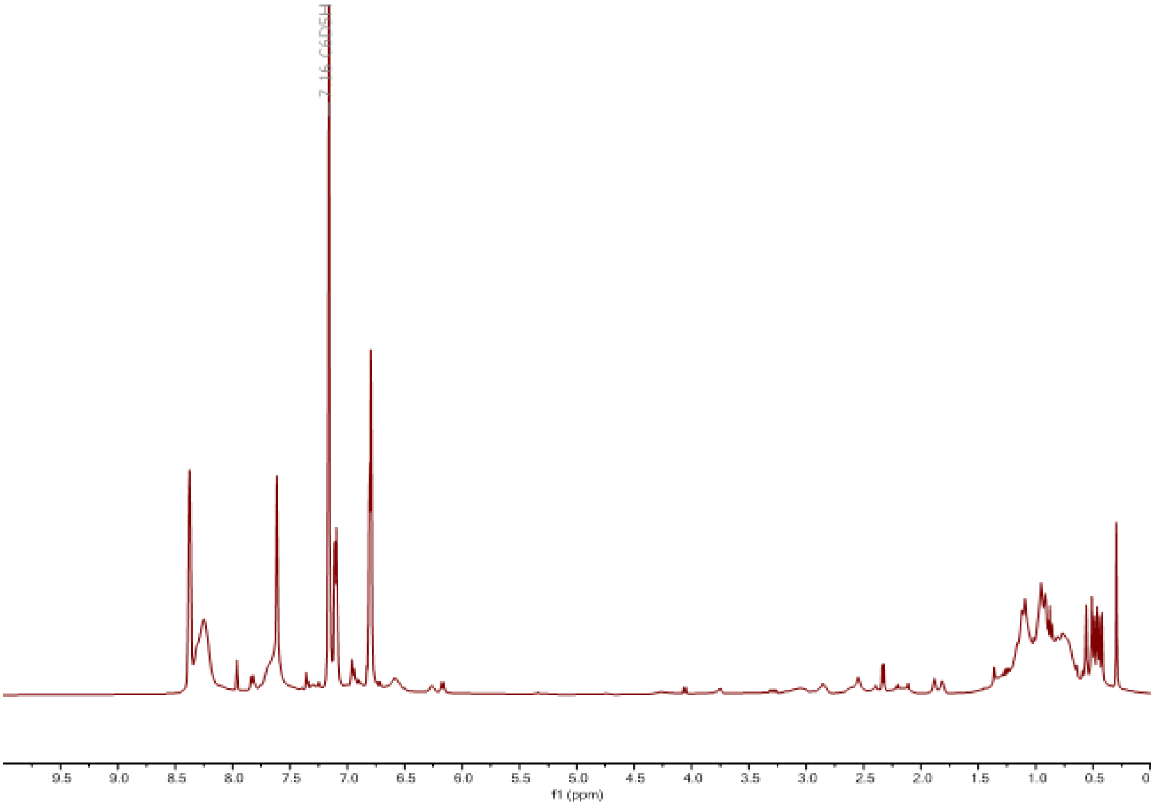

Figure S51: ^{1}H-NMR spectrum of halide abstraction reaction from $^{t\text{-Bu}}$**PNNP**TiCl$_4$ in C_6D_6 at 25 °C.

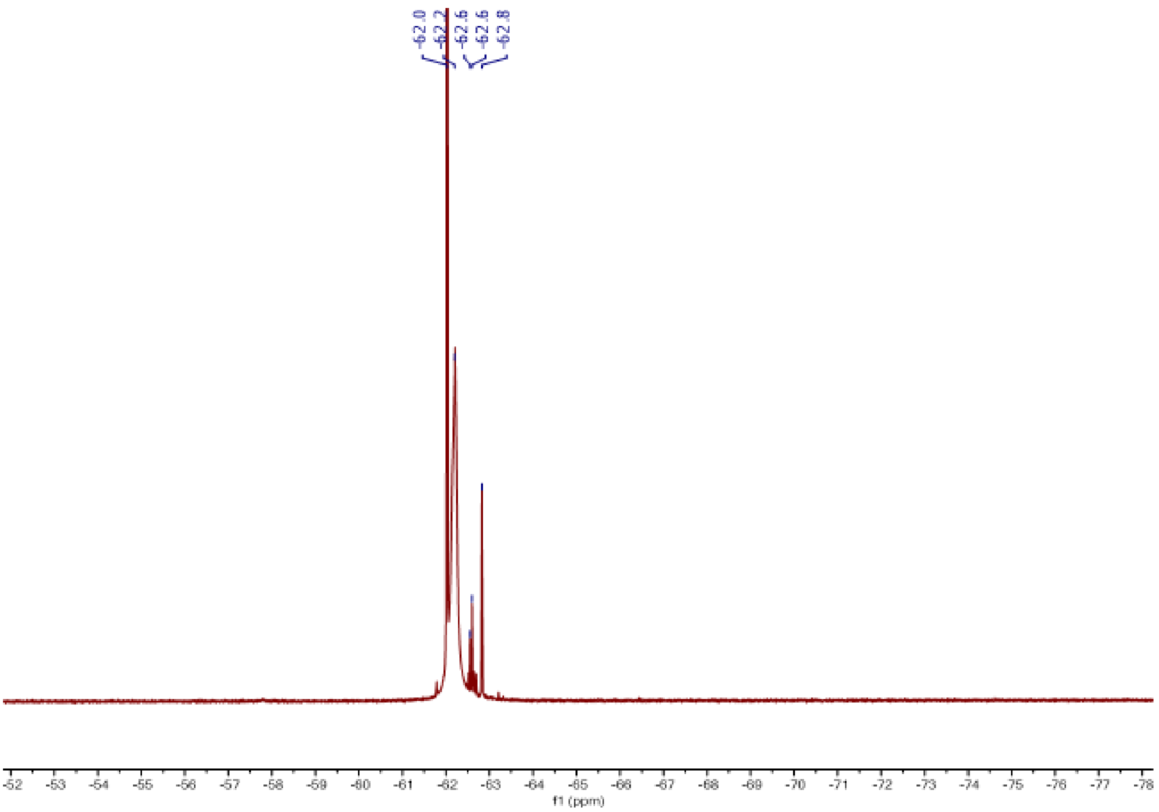

Figure S52: ^{19}F-NMR spectrum of halide abstraction reaction from ***t*-BuPNNP**$TiCl_4$ in C_6D_6 at 25 °C.

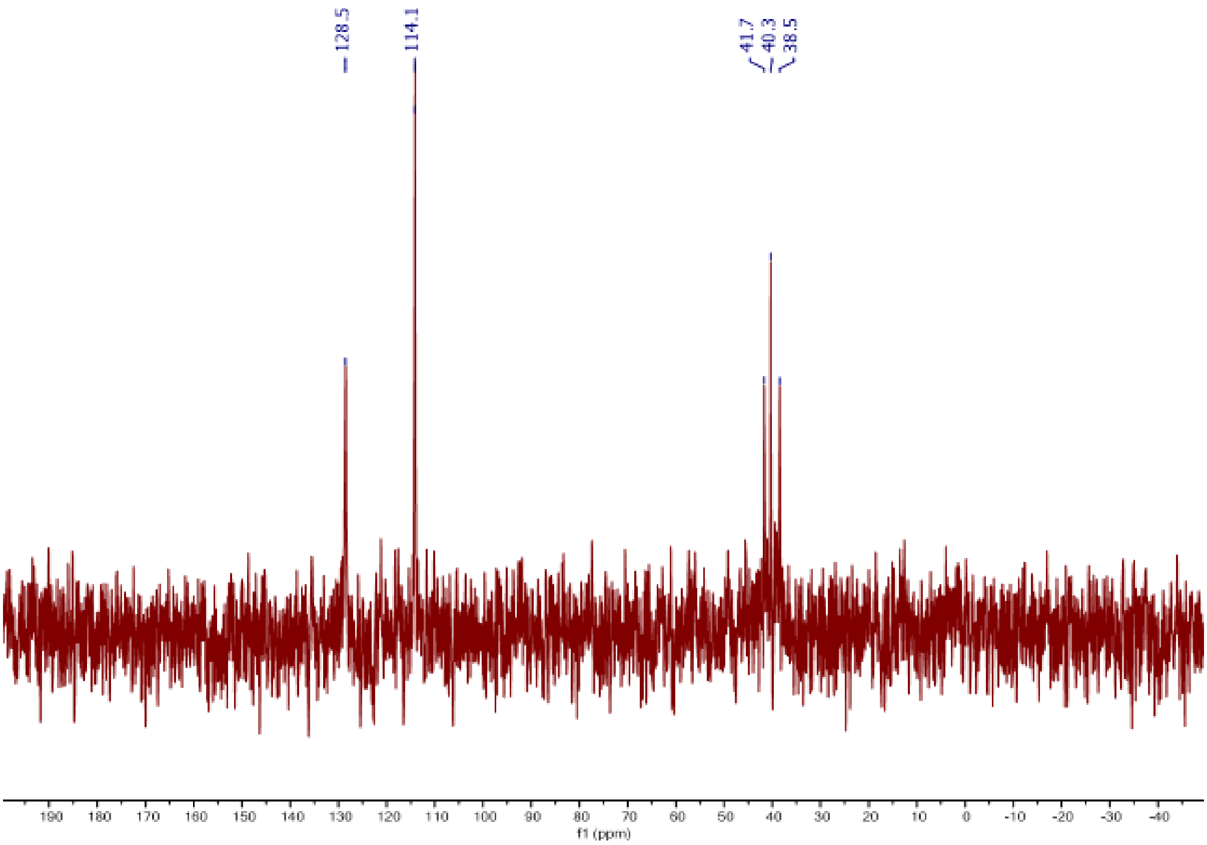

Figure S53: $^{31}P\{^1H\}$-NMR spectrum of halide abstraction reaction from ***t*-BuPNNP**$TiCl_4$ in C_6D_6 at 25 °C.

1.10 Analysis of the halide abstraction from $^{t\text{-Bu}}$**PNN**$^{Me}TiCl_4$:

The unusual way $^{t\text{-Bu}}$**PNNP** binds the $[TiCl_3]^+$ core with both phosphines led us to investigate if a similar Ti-FLP could be established with only one phosphine donor and how this would affect reactivity compared to the Ti-FLT. A solution of $^{t\text{-Bu}}$**PNN**$^{Me}TiCl_4$ in PhCl was treated with one equiv of $NaBArF_{24}$ to generate the respective Ti-FLP. In contrast to **4**, this reaction proceeded all but cleanly. The ^{1}H- and $^{31}P\{^{1}H\}$-NMR spectra show a multitude of resonances which are indicative of a variety of compounds being formed (See **Figures S51-S53**). The $^{31}P\{^{1}H\}$-NMR spectrum shows downfield resonances at 128.5 and 114.1 ppm, suggesting that [$^{t\text{-Bu}}$**PNN**$^{Me}TiCl_3$][$BArF_{24}$] might be present in the mixture. The ^{1}H-NMR spectrum contains many (broad) resonances but diagnostic information on the fate of the reaction can be obtained. Specifically, broad, $BArF_{24}$-derived resonances are present in the region of 7.5-8.5 ppm which are indicative of the anion being chemically non-innocent. This is further confirmed through inspection of the ^{19}F-NMR spectrum, which contains an increased number of (broad) resonances compared to expectations. These observations combined are indicative of decomposition reactions such as activation of the C-F or C-B bonds of the anion by a transient $[TiCl_3]^+$ species. We hypothesise that the lack of the additional phosphine donor leads to insufficient stabilisation of the highly electrophilic Ti centre which results in undesirable side-reactions.

1.11 Reaction of 4 with trans-stilbene oxide (5):

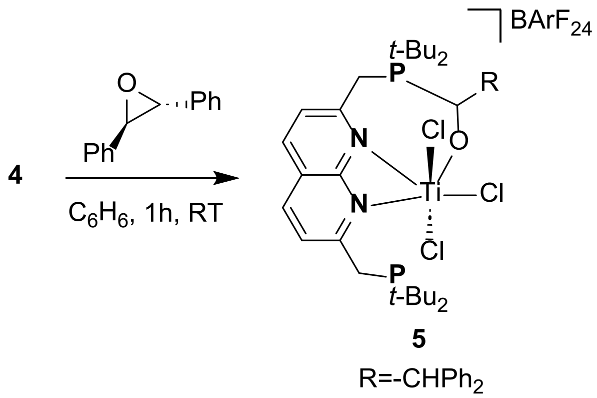

A solution of trans-stilbene oxide (1.3 mg, 6.8 µmol) in benzene (1 mL) was added dropwise to a stirring solution of **4** (10.0 mg, 6.8 µmol) in benzene (1 mL). The mixture was left to stir for 1h at ambient temperature during which the mixture turned orange/red. The benzene was removed *in vacuo* and the residue was washed with pentane (3 x 1 mL). The residue was then dried under reduced pressure resulting in 10.4 mg. reddish solids. Single crystals suitable for XRD were grown by running the same reaction for 1 h in toluene, concentrating the sample until just soluble under reduced pressure and storing it at -40 °C.

Through a combination of integrals, chemical shift and 2D-NMR some of the resonances could be assigned.

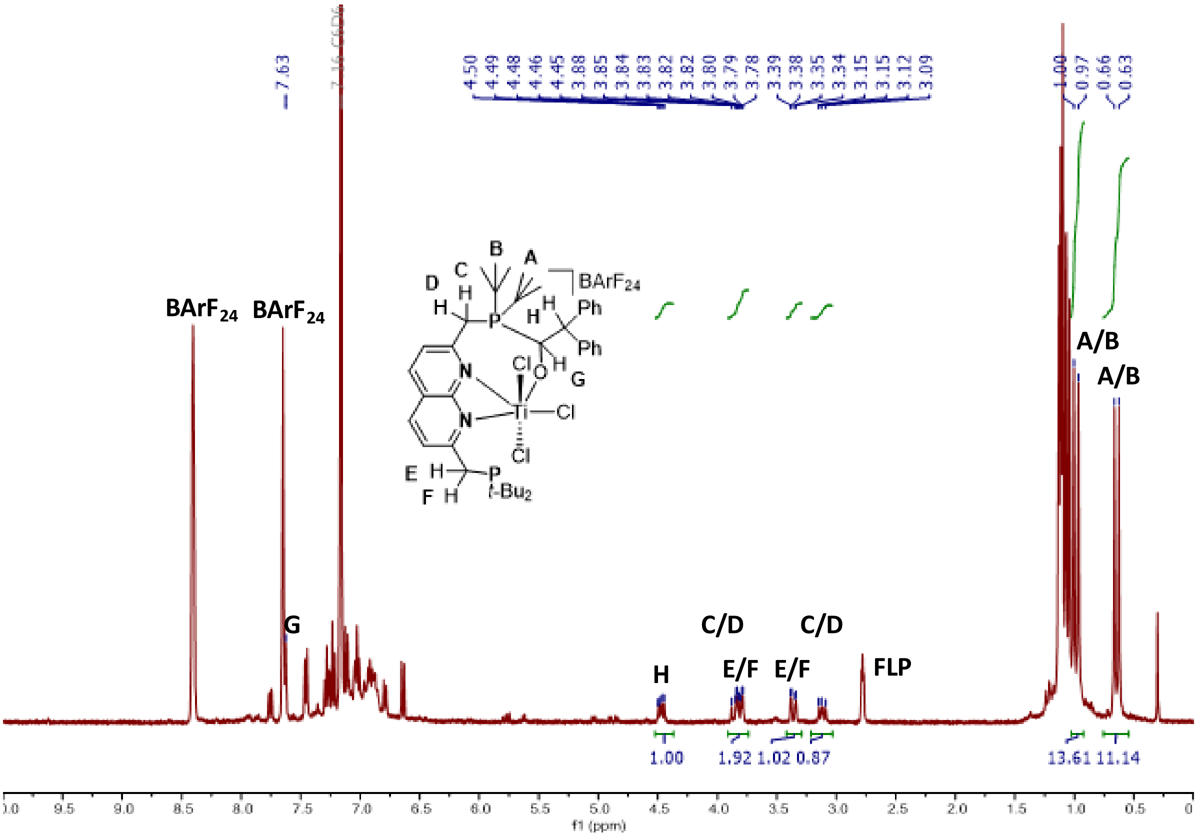

Figure S54: ^{1}H-NMR spectrum of the reaction mixture containing **5** in C_6D_6 at 25 °C.

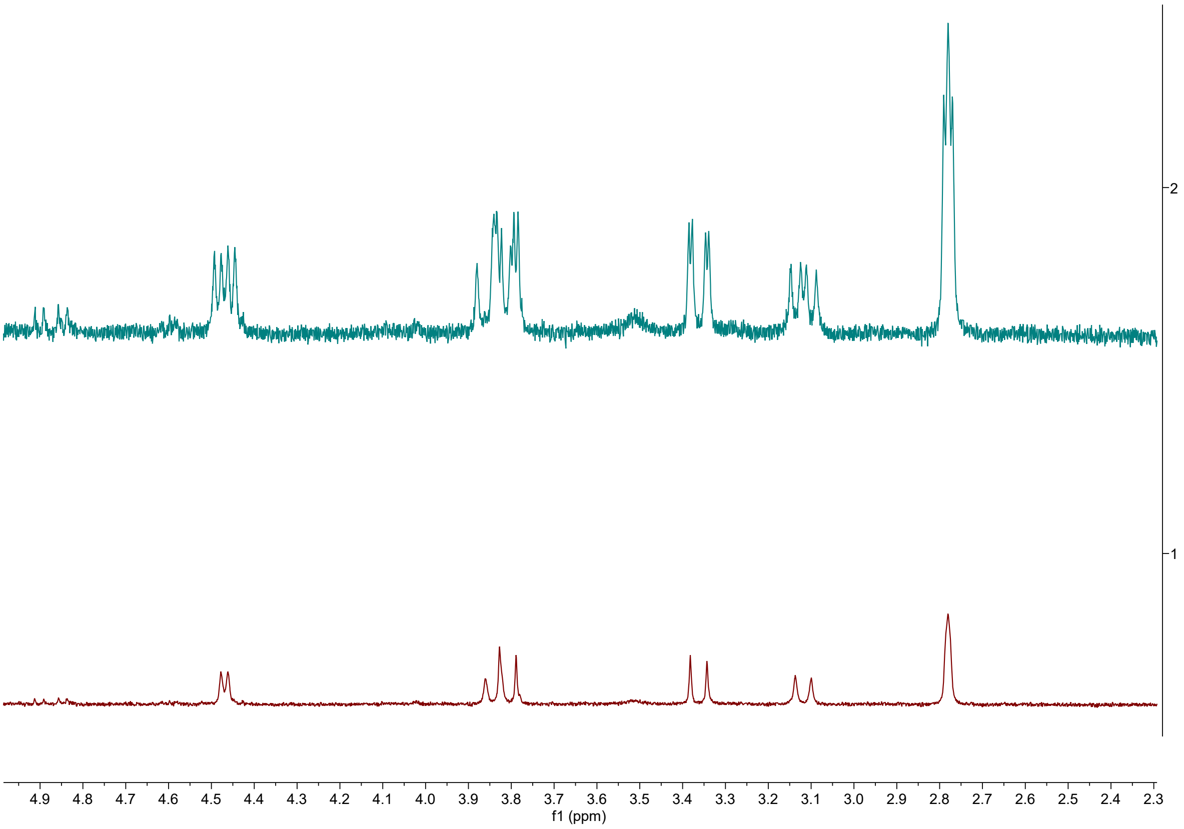

Figure S55: Zoom in of the stack of the ^{1}H-NMR (top) and ^{1}H{^{31}P}-NMR (bottom) spectra of the reaction mixture containing **5** in C_6D_6 at 25 °C.

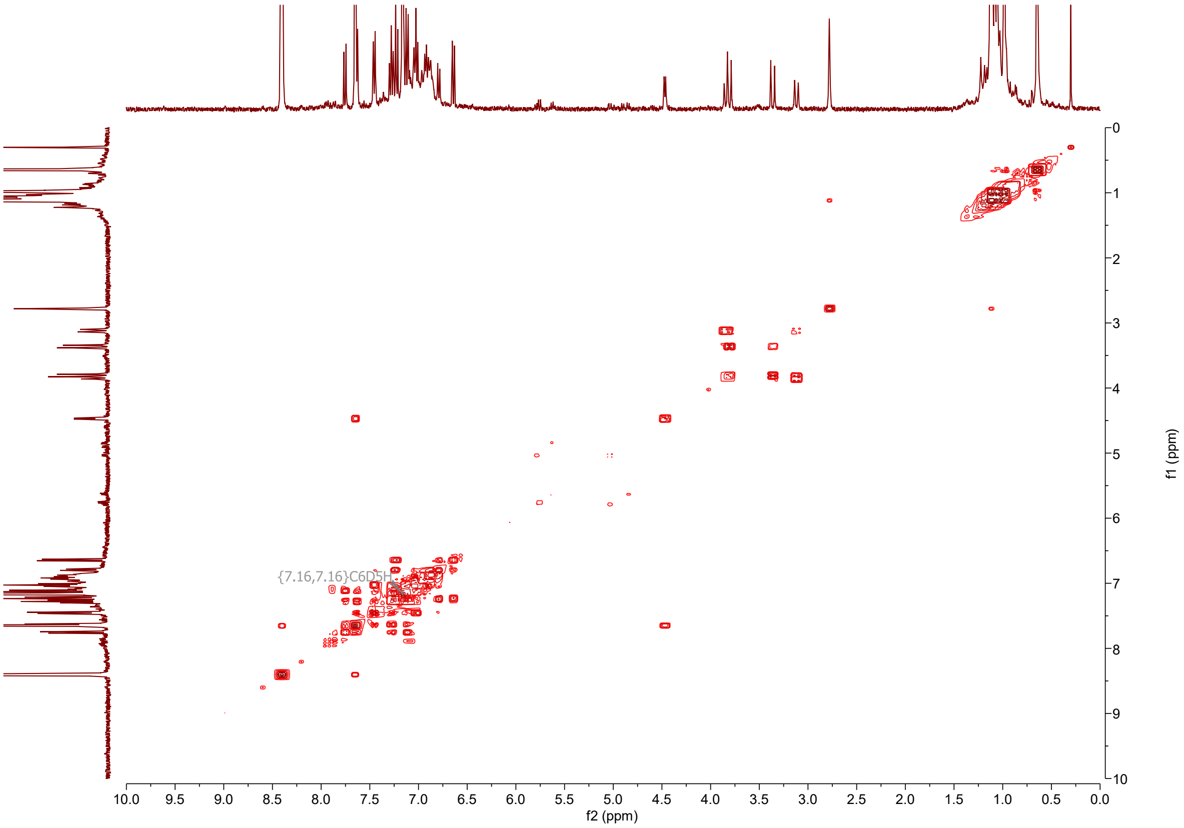

Figure S56: ^{1}H COSY-NMR spectrum of the reaction mixture containing **5** in C_6D_6 at 25 °C.

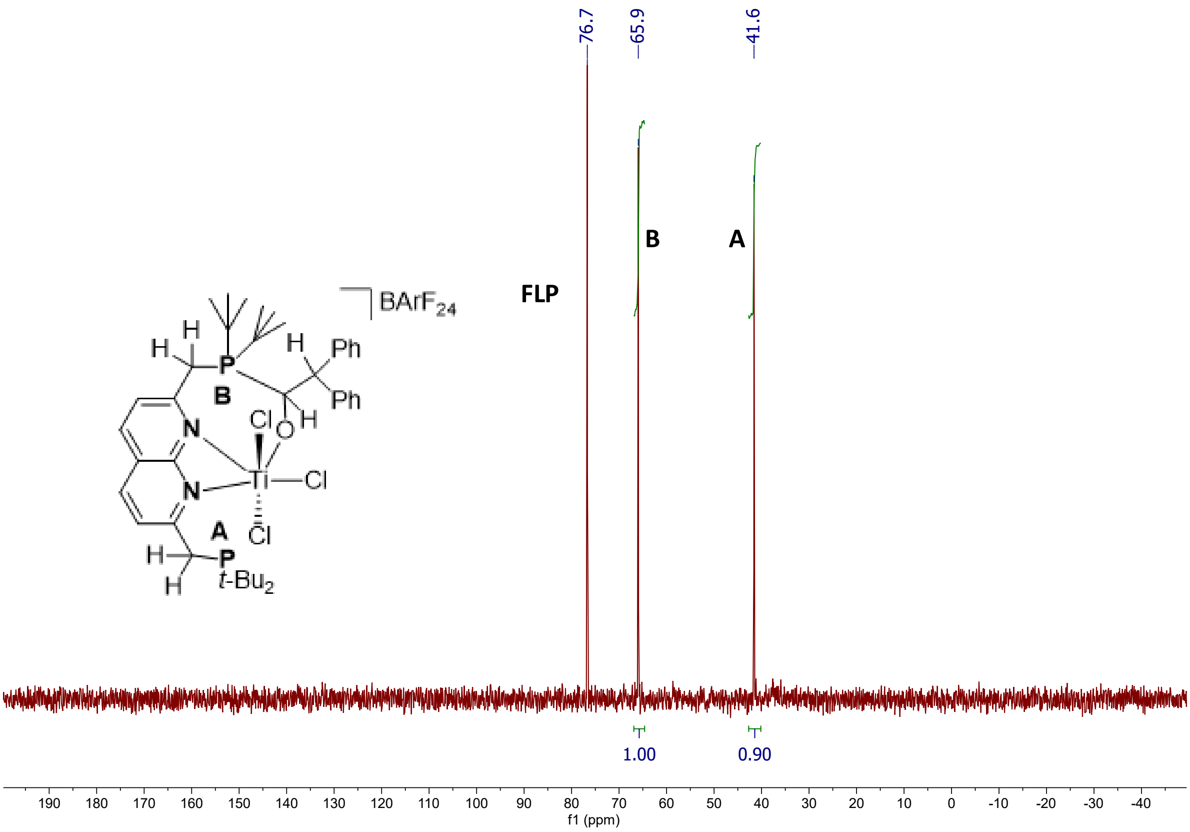

Figure S57: $^{31}P\{^{1}H\}$-NMR spectrum of the reaction mixture containing **5** in C_6D_6 at 25 °C.

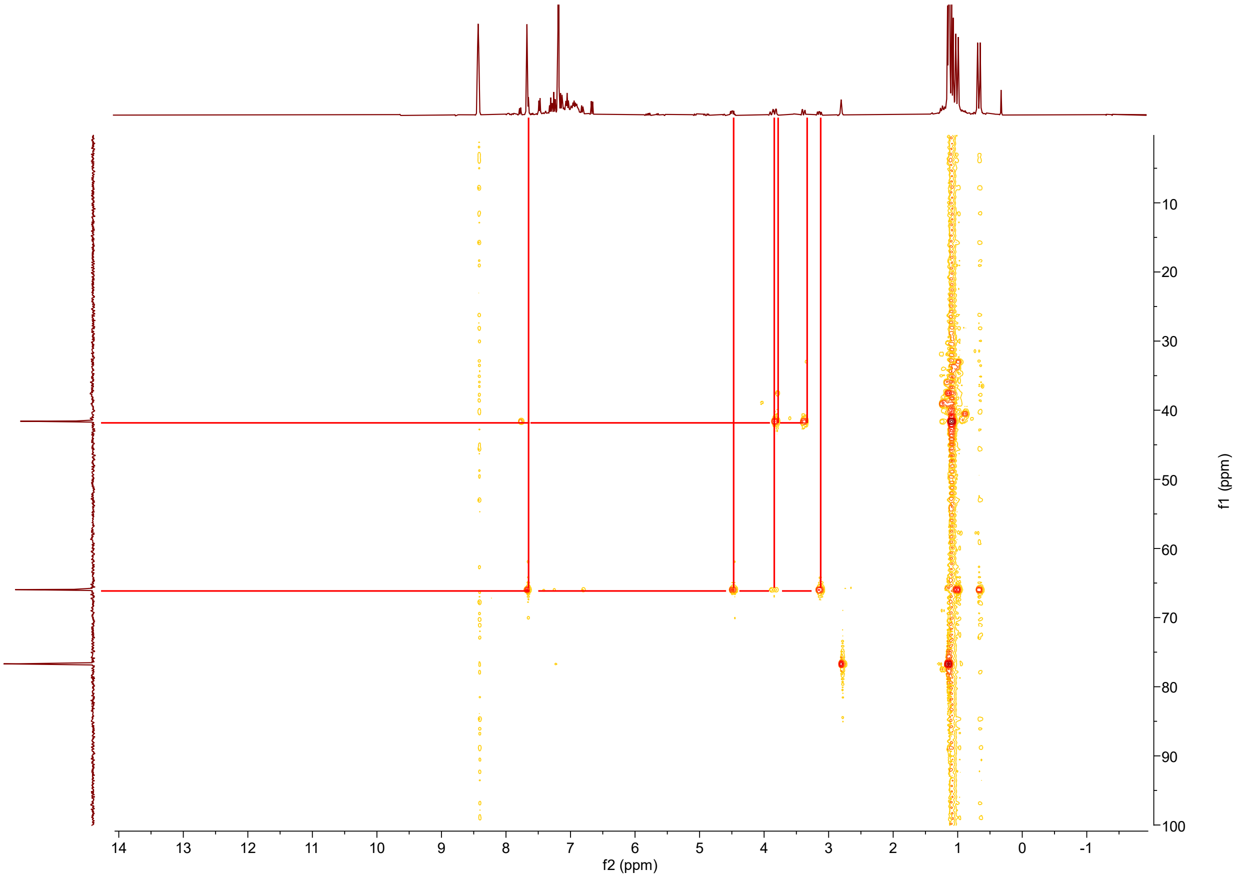

Figure S58: ^{1}H-^{31}P HMBC-NMR spectrum of the reaction mixture containing **5** in C_6D_6 at 25 °C.

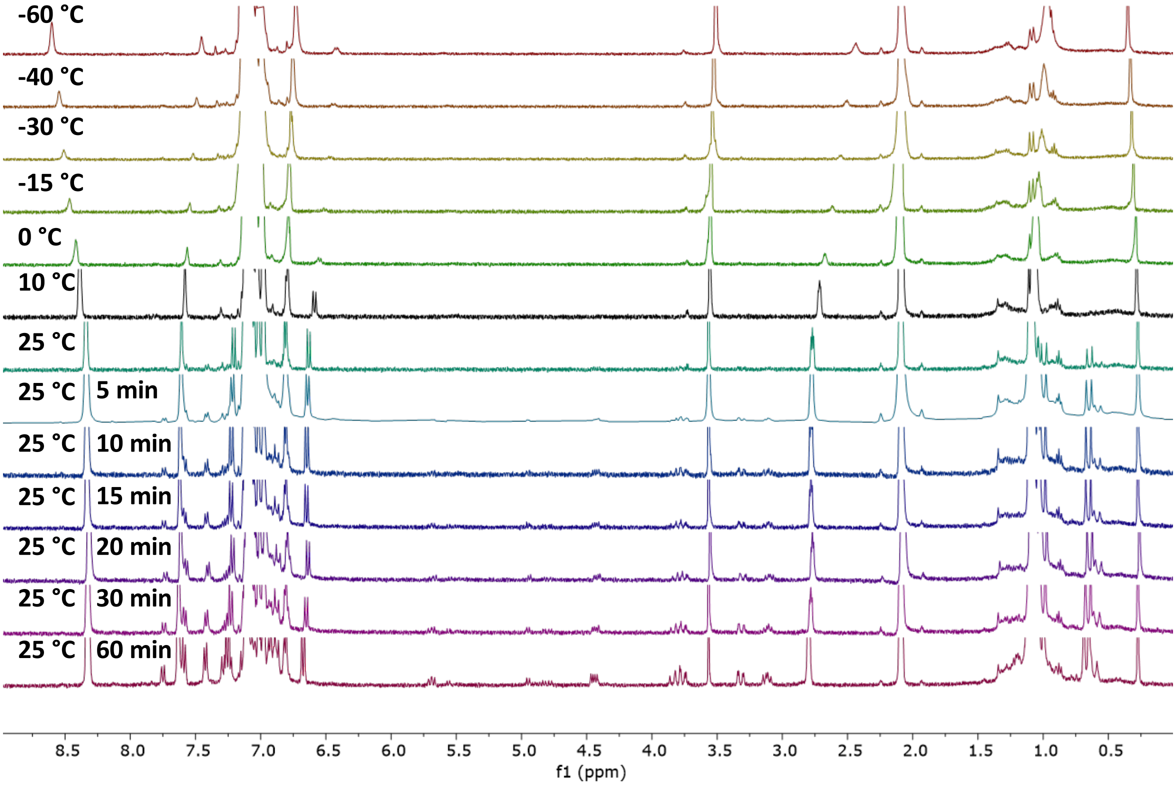

Figure S59: Stacked ^{1}H-NMR spectra at different temperatures of a solution of **4** (5.0 mg) in toluene-d_8 (0.6 mL) that was frozen, layered with a solution of trans-stilbene oxide (0.7 mg) in toluene-d_8 (0.1 mL), thawed and mixed just before entering the spectrometer.

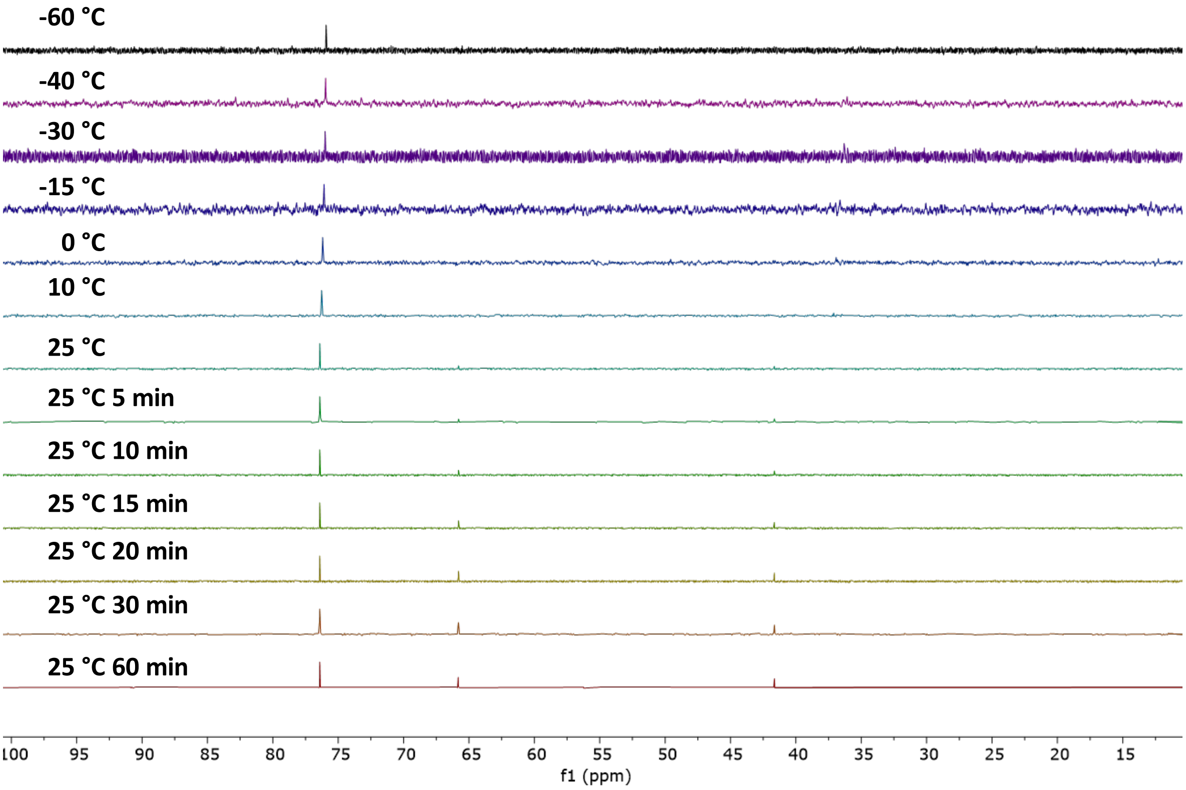

Figure S60: Stacked ^{31}P{^{1}H}-NMR spectra at different temperatures of a solution of **4** (5.0 mg) in toluene-d_8 (0.6 mL) that was frozen, layered with a solution of trans-stilbene oxide (0.7 mg) in toluene-d_8 (0.1 mL), thawed and mixed just before entering the spectrometer.

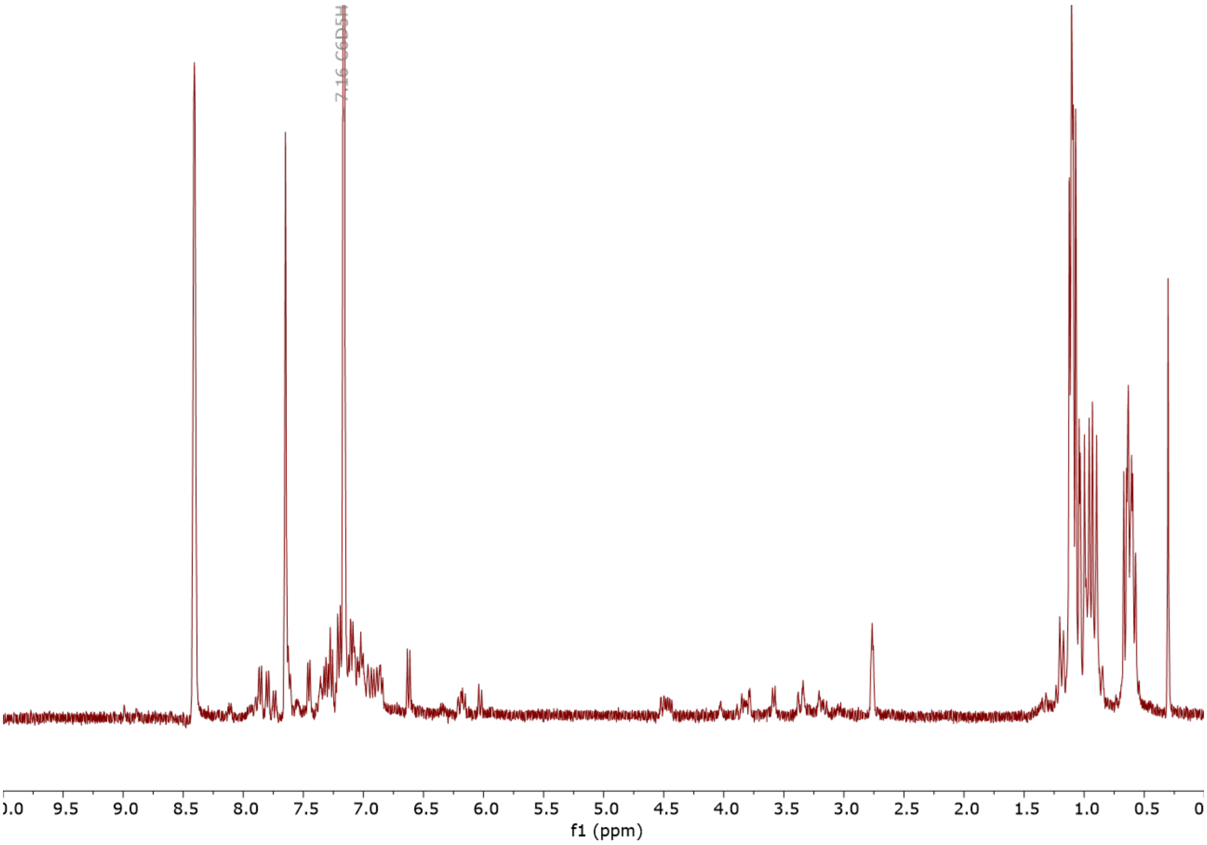

Figure S61: ^{1}H-NMR spectrum of the reaction mixture containing **5** out of the reaction of **4**+epoxide after longer reaction times recorded in C_6D_6 at 25 °C. The resonances between 6.0-6.4 ppm also appear in the reaction of **4**+aldehyde.

1.12 Reaction of 4 with diphenylacetaldehyde:

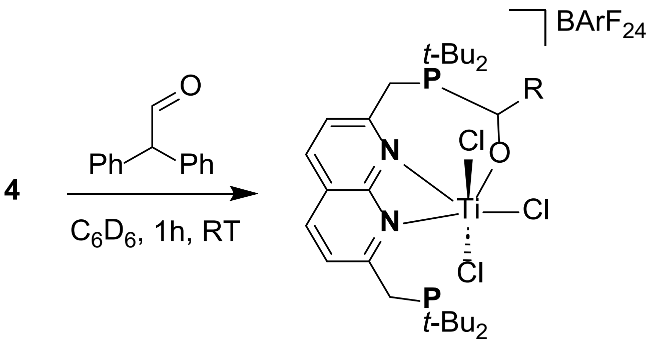

5

R=-CHPh$_2$

Diphenylacetaldehyde (1 µL, 5.7 µmol) was added at once to a stirring solution of **4** (5.0 mg, 3.4 µmol) in C_6D_6 (0.6 mL). After stirring at ambient temperature for 1 h, the mixture was transferred to a J-Young's tube and immediately measured.

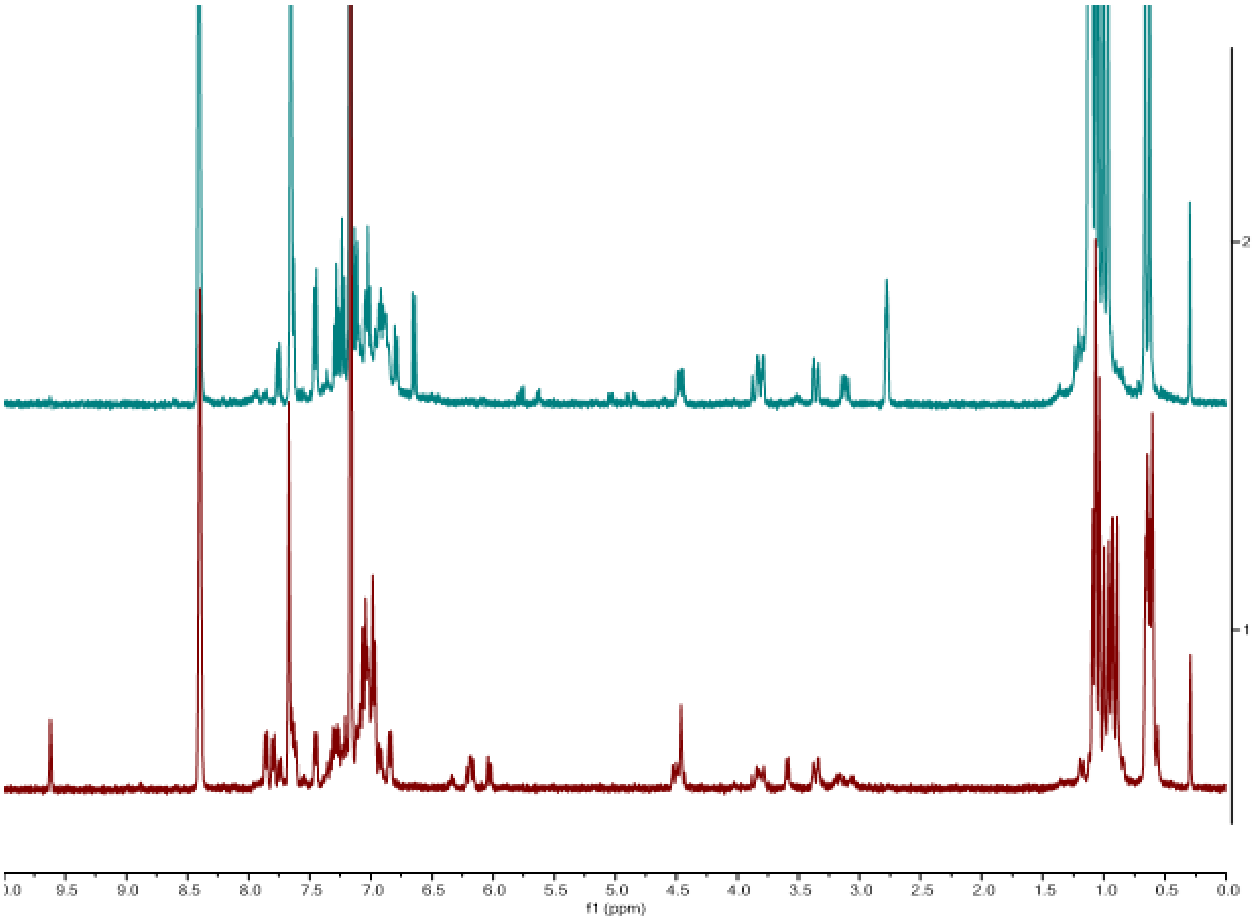

Figure S62: Stacked ^{1}H-NMR spectra of the reaction mixtures containing **5** out of the reactions of **4**+epoxide (top) and **4**+aldehyde (bottom) in C_6D_6 at 25 °C.

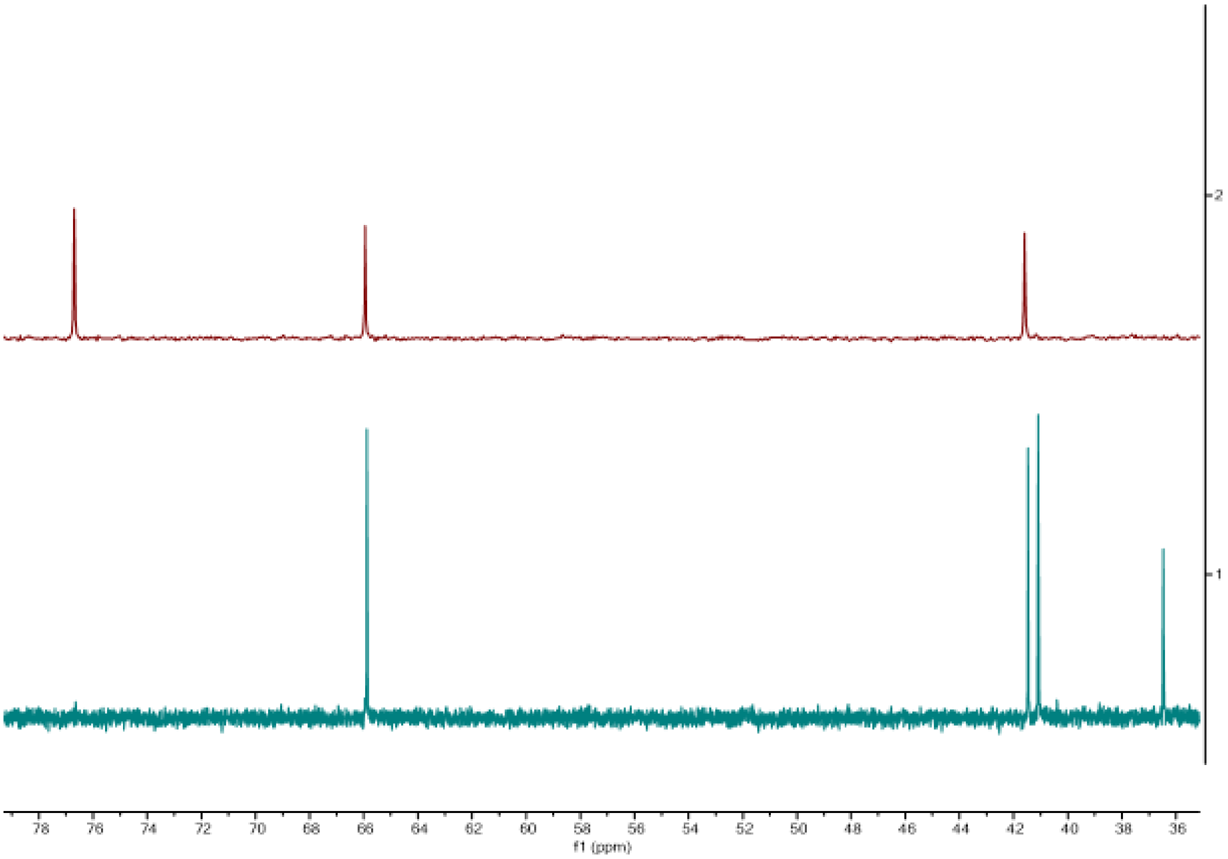

Figure S63: Stacked $^{31}P\{^{1}H\}$-NMR spectra of the reaction mixtures containing **5** out of the reactions of **4**+epoxide (top) and **4**+aldehyde (bottom) in C_6D_6 at 25 °C.

1.13 Reaction of 4 with phenyl isocyanate (6):

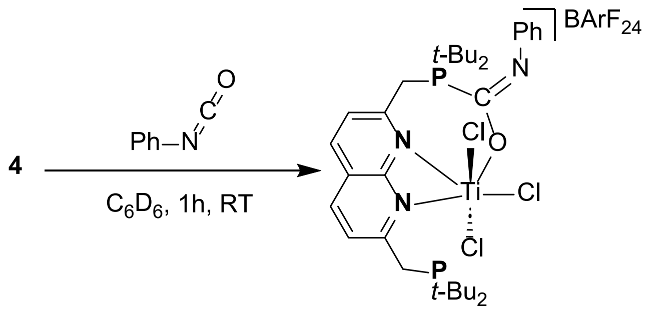

6

Phenyl isocyanate (0.4 µL, 3.4 µmol) was added by microsyringe to a stirring solution of **4** (5.0 mg, 3.4 µmol) in C_6D_6 (0.6 mL) at ambient temperature. The colour changed rapidly to dark brown/red and after 1 h of stirring the mixture was analysed.*

*Simple work-up by pumping down, washing with minimal pentane and drying led consistently to decomposition.

Through a combination of integrals, chemical shift and 2D-NMR some of the following resonances could be assigned:

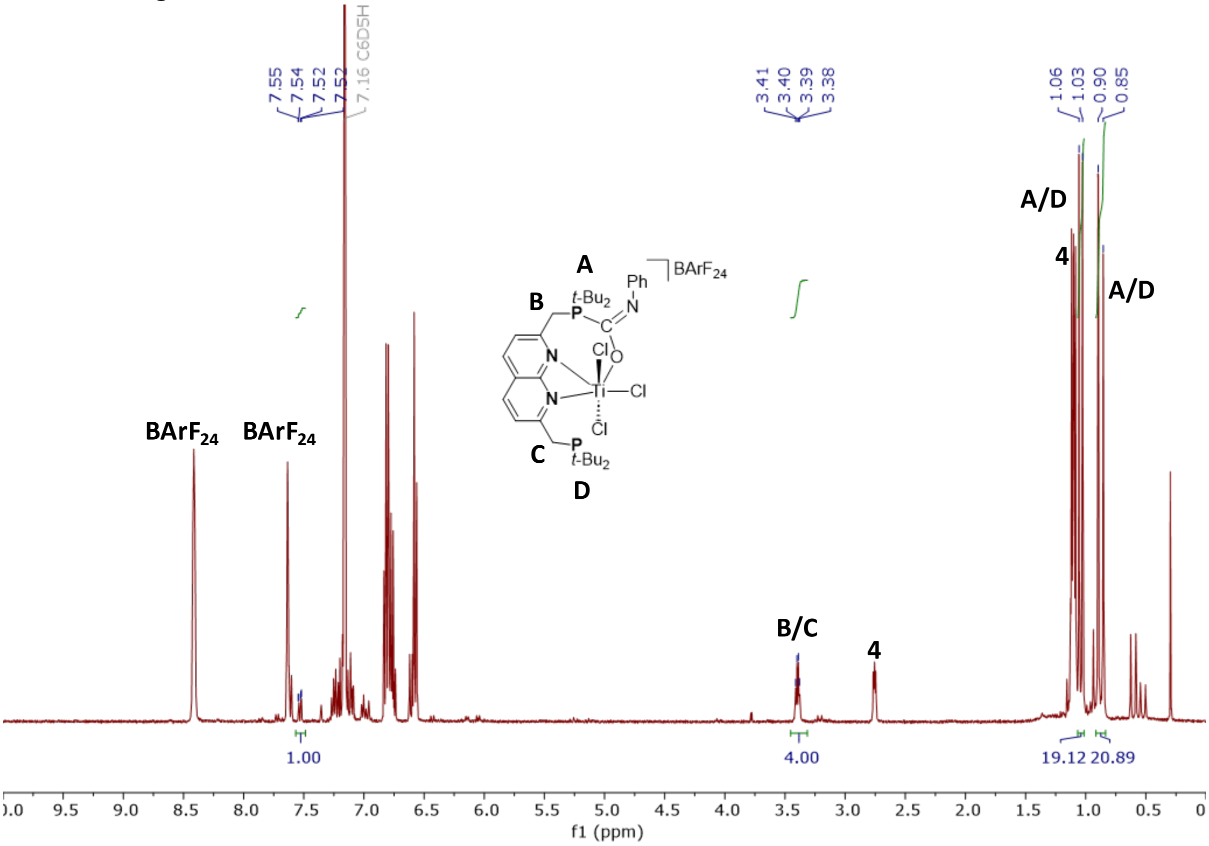

Figure S64: ^{1}H-NMR spectrum of the reaction mixture containing **6** in C_6D_6 at 25 °C.

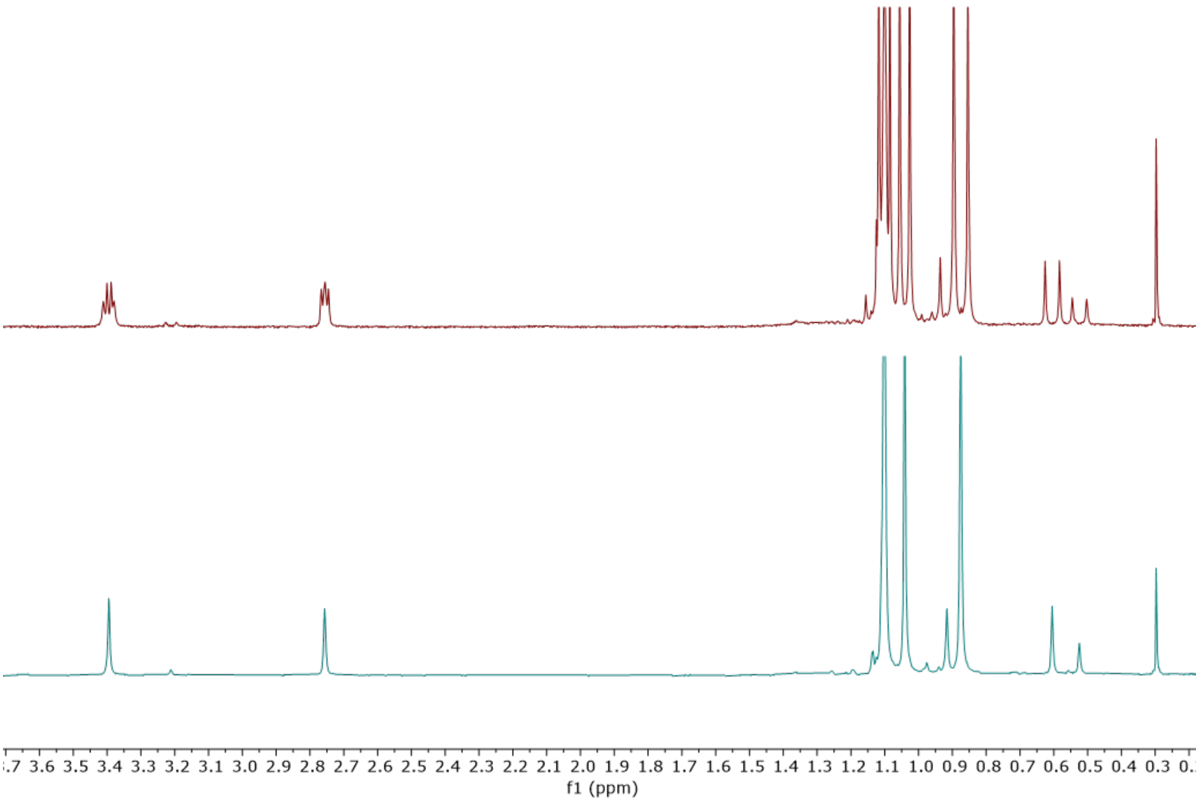

Figure S65: Zoom in of the stack of the ^{1}H-NMR (top) and ^{1}H{^{31}P}-NMR (bottom) spectra of the reaction mixture containing **6** in C_6D_6 at 25 °C.

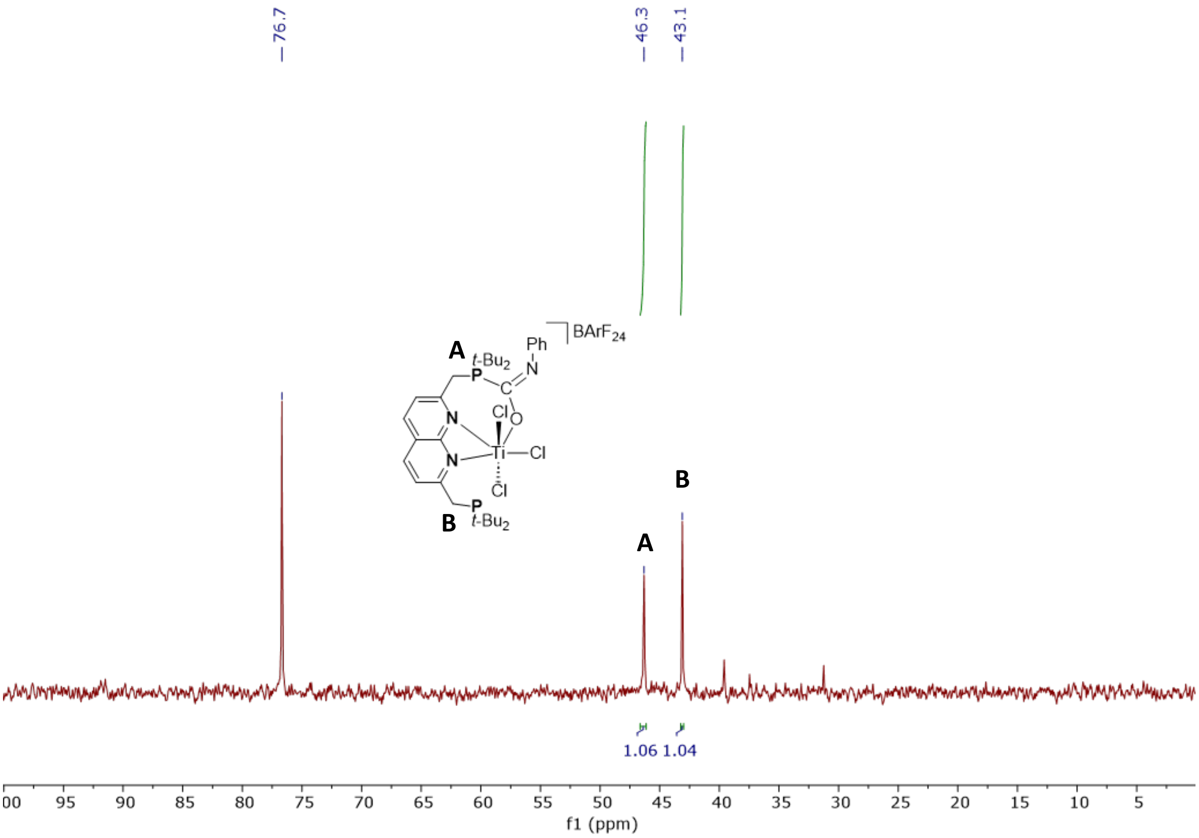

Figure S66: ^{31}P{^{1}H}-NMR spectrum of the reaction mixture containing **6** in C_6D_6 at 25 °C.

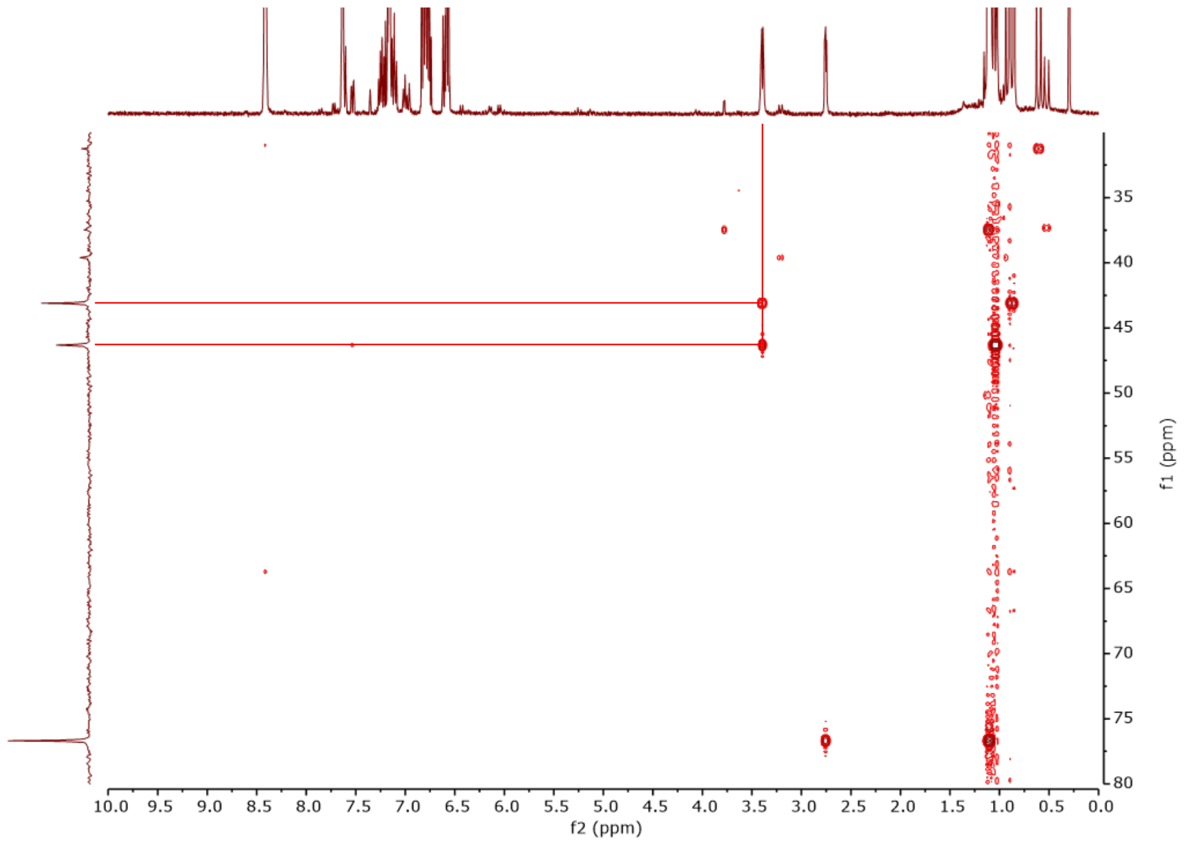

Figure S67: ^{1}H-^{31}P HMBC-NMR spectrum of the reaction mixture containing **6** in C_6D_6 at 25 °C.

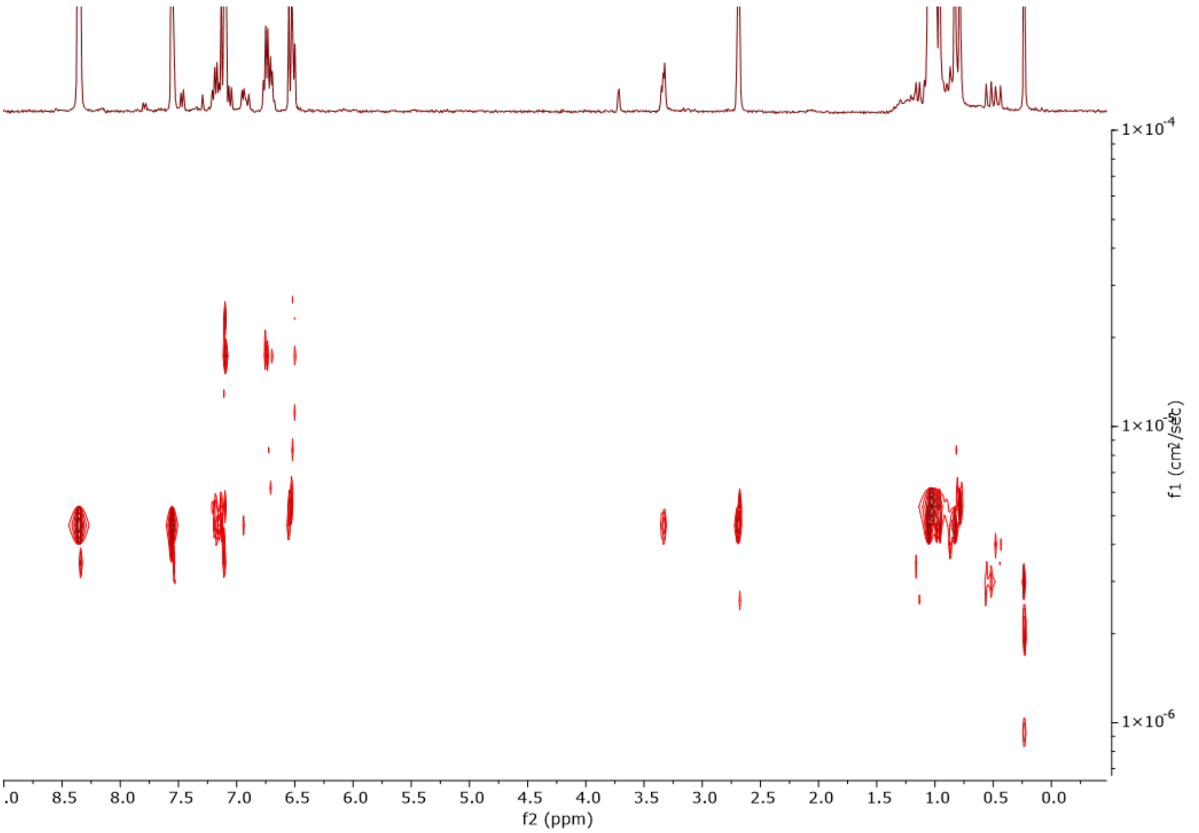

Figure S68: DOSY-NMR spectrum of the reaction mixture containing **6** in C_6D_6 at 25 °C. Measured in 32 increments from 5 to 80 Gcm^{-1}. Gradient length = 1 ms, diffusion delay = 45 ms.

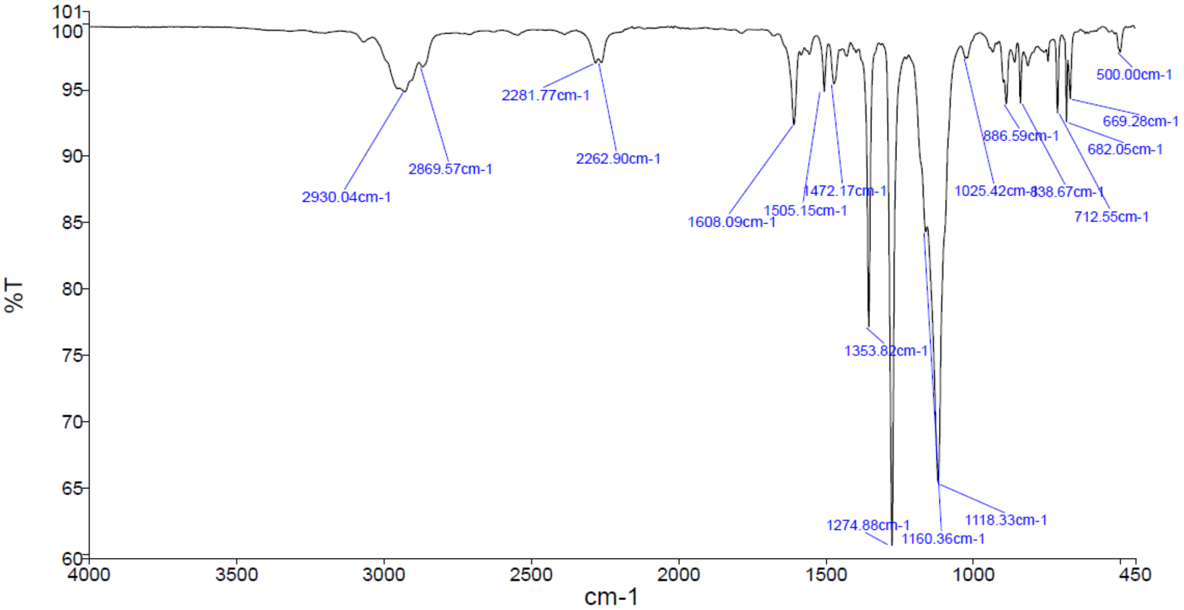

Figure S69: The ATR-IR spectrum of the reaction mixture of **4** with phenyl isocyanate measured as a film under N_2 flow at 25 °C. **Note:** Due to the observed instability of **6** upon removing the solvent and the method of recording the IR-spectrum we cannot exclude the possibility of **6** not being present in the spectrum or all the unreacted phenyl isocyanate not having evaporated.

2. Computational Methods

2.1 General remarks:

Calculations were performed using the Gaussian 09 rev. D.01 and Gaussian 16 rev. C.01 software.[5,6] The Becke 1988 exchange functional (B3LYP) was used.[7,8] The SDD basis set was used on Ti[9] and the 6-31G** basis set on the remaining elements for the geometry optimizations.[10–14] For single point calculations the 6-311G** basis set was used for the non-Ti elements.[11,15,16] Starting geometries for the optimizations were obtained from the coordinates of the crystal structures if possible, or by modification of the optimized geometry of the most similar complex. Additionally, Grimme's DFT-D3 scheme for atom-pairwise dispersion correction was used for all atoms in every calculation.[17,18] The absence of imaginary frequencies was checked to confirm that the optimised structures correspond to real local minima. A solvent model (SMD) for benzene was included in the single point calculations. The energies of the optimised structures were calculated by taking the SCF energy from the SP calculations and applying the thermal correction of the Gibbs energy obtained from the geometry optimisation/frequency calculations. NBO[19] calculations were performed using the NBO 6.0 software.[20] Natural population analysis,[21,22] Mulliken population analysis[23] and computation of Wiberg bond indices[24] were also performed using the NBO 6.0 software. PES scans were performed by using the opt=modredundant keyword and scanning along the desired coordinate. Input and output files can be downloaded free of charge from the Yoda data repository DOI: https://doi.org/10.24416/UU01-Q4AU5Q.

2.2 Example input file for geometry optimisation:

```
#p
scf=(maxcycle=300)
opt
freq=noraman
B3LYP/gen
EmpiricalDispersion=GD3
pseudo=read
nosym
int=ultrafine

"Title"

1 1
[cartesian coordinates here]

Cl P N C H O 0
6-31G**
****
Ti 0
SDD
****

Ti 0
SDD
```

2.3 Example input file for SP calculations:

```
#p
scf=(maxcycle=300)
SP
SCRF(SMD,Solvent=Benzene)
B3LYP/gen
EmpiricalDispersion=GD3
pseudo=read
nosym
int=ultrafine

"Title"

1 1
[cartesian coordinates here]

Cl P N C H O 0
6-31G**
****
Ti 0
SDD
****

Ti 0
SDD
```

2.4 Example input file for NBO calculations:

```
#p
B3LYP/gen
pop=NBO6Read
EmpiricalDispersion=GD3
pseudo=read
nosym
int=ultrafine

"Title"

1 1
[cartesian coordinates here]

$nbo plot archive file="name" nlmo bndidx $end
[Choose keylist]
```

Note: In the case of **4**⁺, running the NBO calculation kept converging to a non-intuitive Lewis structure (*i.e.* lone pairs on naphthyridine carbons) and as such we used the Choose keylist to enforce an intuitive Lewis structure. For the sake of consistency, similar constricts were applied to the NBO calculation of the titanocene-based Ti-FLP for proper comparison.

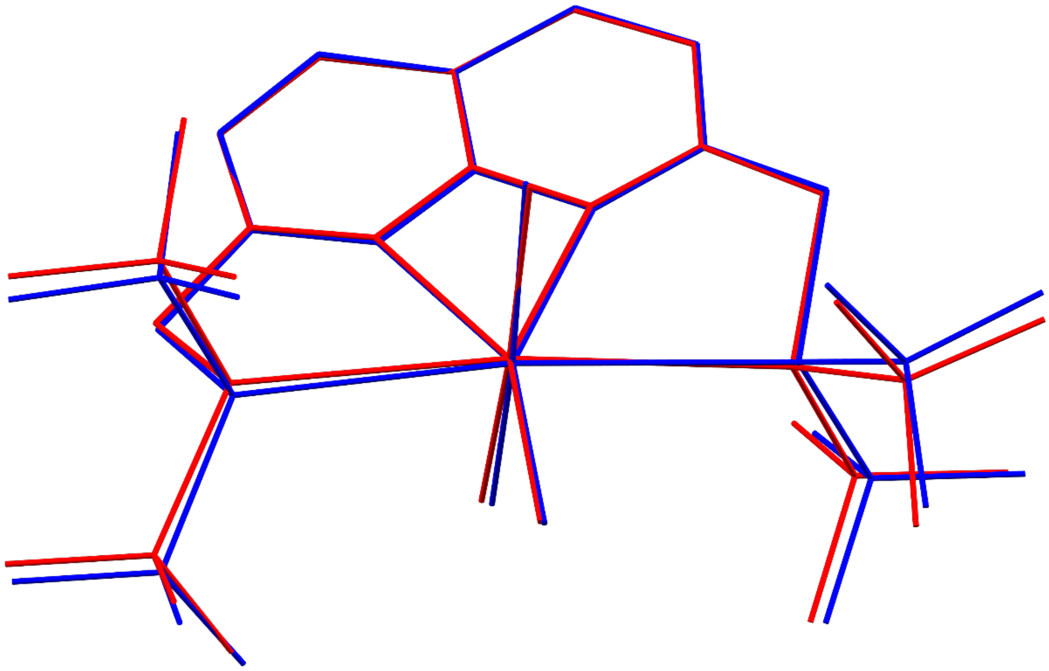

Figure S70: Overlay of the solid-state structure of the cation of **4** (red) and the gas-phase optimized structure of **4**$^{+}$ (blue) depicted in wireframe.

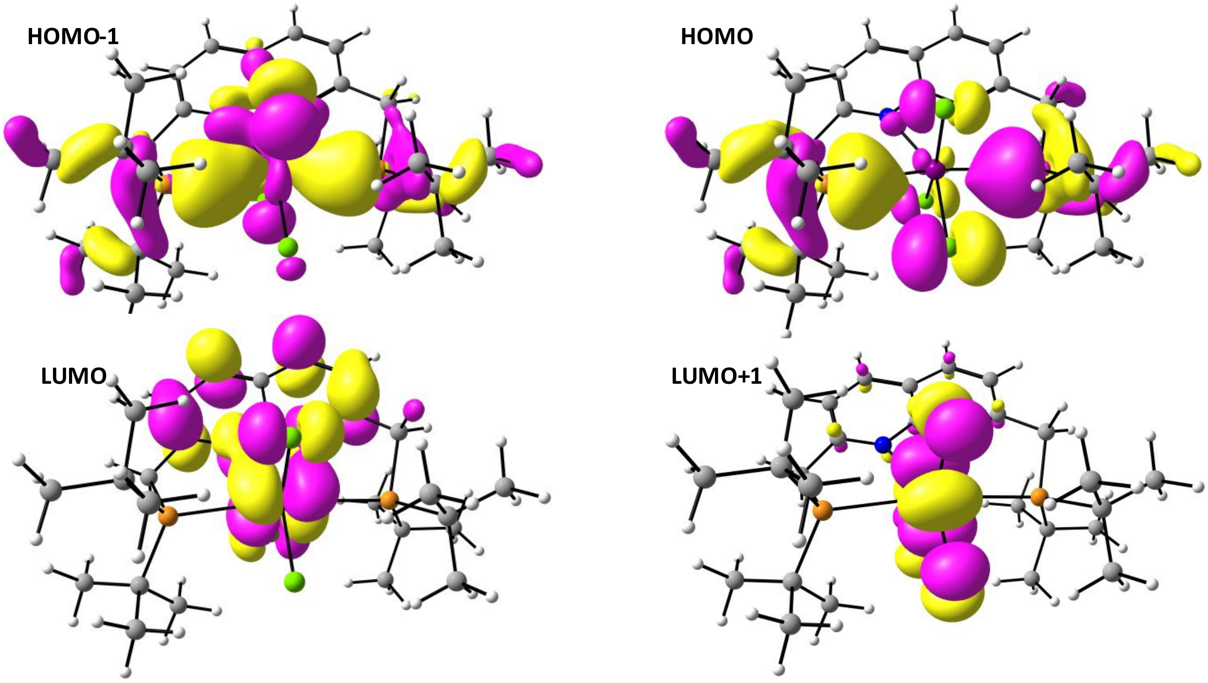

Figure S71: Selected frontier orbitals found computationally for **4**$^{+}$. Bonding Ti-P interactions with large coefficients can be found along the FLT vector for the HOMO-1 and HOMO, whereas the LUMO and LUMO+1 are dominated by Ti-Cl antibonding interactions.

2.5 XYZ Coordinates and energies of 4^{+}:

Electronic energy from SP calculation: -3249.1072522500 hartrees
Gibbs thermal correction: 0.5994440000 hartrees

Ti	13.348068000	3.900346000	3.769047000
Cl	15.008382000	5.357048000	3.189446000
Cl	11.816794000	2.209772000	3.864375000
Cl	12.818499000	4.862272000	5.752460000
P	11.352365000	5.623540000	2.649511000
P	15.245535000	2.367352000	5.265808000
N	12.962777000	3.588707000	1.595513000
N	14.639997000	2.564866000	2.538748000
C	10.924661000	4.684819000	1.067526000
C	12.106243000	3.864434000	0.630254000
C	12.338081000	3.315562000	-0.665845000
C	13.423938000	2.487025000	-0.904171000
C	14.313086000	2.157639000	0.159684000
C	13.984059000	2.747056000	1.384359000
C	15.477707000	1.337369000	0.200854000
C	16.182705000	1.197111000	1.386471000
C	15.746867000	1.847529000	2.578876000
C	16.459202000	1.883351000	3.902360000
C	11.976901000	7.307683000	1.987895000
C	12.995154000	7.022922000	0.859768000
C	12.677947000	8.061814000	3.138791000
C	10.861553000	8.193116000	1.399534000
C	9.671160000	5.795488000	3.542787000
C	8.500269000	6.113232000	2.588726000
C	9.376313000	4.450046000	4.237836000
C	9.784132000	6.887016000	4.627125000
C	16.342491000	3.256985000	6.553925000
C	17.714835000	2.581268000	6.760128000
C	15.583366000	3.337935000	7.894621000
C	16.583533000	4.693679000	6.046117000
C	14.630767000	0.672732000	5.910471000
C	14.242092000	-0.185401000	4.684563000
C	15.692273000	-0.105980000	6.710982000
C	13.386403000	0.902907000	6.795415000
H	10.120416000	3.984387000	1.324529000
H	10.546919000	5.333599000	0.273797000
H	11.646948000	3.552558000	-1.467248000
H	13.590260000	2.082908000	-1.898324000
H	15.819463000	0.830786000	-0.696861000
H	17.081891000	0.591665000	1.417677000
H	17.205852000	2.685243000	3.846866000
H	16.995781000	0.955998000	4.115934000
H	12.513131000	6.620534000	-0.037033000
H	13.790747000	6.345462000	1.168642000
H	13.464401000	7.969363000	0.570345000
H	13.482911000	7.476270000	3.583615000

H	11.982476000	8.344409000	3.930608000
H	13.113386000	8.984938000	2.739903000
H	11.325203000	9.083063000	0.958645000
H	10.156516000	8.539301000	2.156637000
H	10.301803000	7.693321000	0.602678000
H	7.581456000	6.169436000	3.183514000
H	8.346324000	5.327798000	1.842816000
H	8.608416000	7.064501000	2.069618000
H	10.133476000	4.200881000	4.981935000
H	9.306778000	3.617420000	3.532096000
H	8.408994000	4.529169000	4.746457000
H	9.863071000	7.890470000	4.204103000
H	10.635102000	6.715441000	5.291519000
H	8.875553000	6.864138000	5.238837000
H	18.278629000	3.170803000	7.492043000
H	18.311200000	2.561818000	5.843069000
H	17.645482000	1.565672000	7.147108000
H	15.490009000	2.365516000	8.382230000
H	14.586450000	3.769865000	7.773556000
H	16.146414000	3.987715000	8.573571000
H	15.652732000	5.253112000	5.947712000
H	17.098731000	4.718926000	5.081752000
H	17.220462000	5.213095000	6.770814000
H	13.761319000	-1.102148000	5.042579000
H	15.117536000	-0.492517000	4.103293000
H	13.535997000	0.316339000	4.023380000
H	15.942957000	0.377222000	7.656360000
H	16.614999000	-0.266074000	6.144423000
H	15.285376000	-1.094996000	6.951032000
H	12.980064000	-0.071135000	7.090356000
H	12.602699000	1.447672000	6.268457000
H	13.625778000	1.447421000	7.710120000

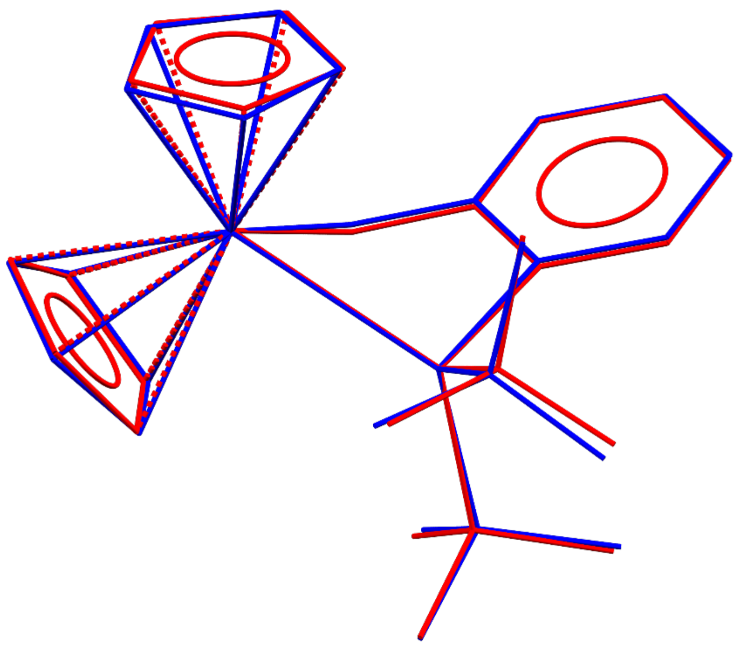

Figure S72: Overlay of the solid-state structure of the cation of **titanocene phosphinoaryloxide** (red) and the gas-phase optimized structure of **titanocene phosphinoaryloxide** (blue) depicted in wireframe.

2.6 XYZ Coordinates of the titanocene phosphinoaryloxide:

Ti	-3.097637000	13.071408000	22.595294000
P	-4.554514000	12.047096000	20.458890000
O	-4.478812000	12.015402000	23.304187000
C	-2.401170000	15.376015000	22.615824000
H	-1.368323000	15.638255000	22.440606000
C	-2.990102000	15.055272000	23.872135000
H	-2.488612000	15.049551000	24.830063000
C	-4.369127000	14.802608000	23.653367000
H	-5.087462000	14.504890000	24.404152000
C	-4.620156000	14.904931000	22.273986000
H	-5.576340000	14.749349000	21.802829000
C	-3.391778000	15.247301000	21.627623000
H	-3.242108000	15.421557000	20.573395000
C	-1.546817000	12.075346000	24.095671000
H	-1.769058000	12.072591000	25.154547000
C	-1.919569000	11.075927000	23.168941000
H	-2.509018000	10.205299000	23.404186000
C	-1.479800000	11.480846000	21.885932000
H	-1.597113000	10.935881000	20.964085000
C	-0.806562000	12.728167000	22.025322000
H	-0.356428000	13.300605000	21.225239000
C	-0.822110000	13.074295000	23.393677000
H	-0.379937000	13.955631000	23.833427000
C	-5.773304000	12.066783000	22.899676000
C	-6.810588000	12.051814000	23.838024000
H	-6.564439000	12.004173000	24.893678000
C	-8.131933000	12.101113000	23.396577000

H	-8.937970000	12.097608000	24.123725000
C	-8.426806000	12.149166000	22.029488000
H	-9.458281000	12.179514000	21.694755000
C	-7.391967000	12.143351000	21.094023000
H	-7.630648000	12.154565000	20.036213000
C	-6.054368000	12.112042000	21.513034000
C	-4.812277000	13.049911000	18.828423000
C	-5.562335000	14.372859000	19.098163000
H	-5.005778000	15.069301000	19.717914000
H	-6.542425000	14.212291000	19.552353000
H	-5.725584000	14.872943000	18.137625000
C	-3.392249000	13.361278000	18.302010000
H	-3.466140000	14.002054000	17.416593000
H	-2.853001000	12.457690000	18.007118000
H	-2.784823000	13.885831000	19.045344000
C	-5.604975000	12.324500000	17.719884000
H	-6.641264000	12.132982000	18.007754000
H	-5.152564000	11.387252000	17.401021000
H	-5.632219000	12.980226000	16.842574000
C	-4.458846000	10.154594000	20.027823000
C	-3.387773000	9.880001000	18.953646000
H	-3.257428000	8.796535000	18.858712000
H	-2.411322000	10.300926000	19.211301000
H	-3.662768000	10.257639000	17.968233000
C	-4.093321000	9.363197000	21.302391000
H	-4.671368000	9.675034000	22.174819000
H	-3.033452000	9.426199000	21.536559000
H	-4.311668000	8.305573000	21.121938000
C	-5.832433000	9.615492000	19.566154000
H	-5.704828000	8.561509000	19.294851000
H	-6.244603000	10.121939000	18.697164000
H	-6.567499000	9.659511000	20.372302000

2.7 NBO Donor-Acceptor interactions of the titanocene phosphinoaryloxide

The NLMO/NPA bond order found for the Ti-P bond of the titanocene-based compound was 0.4 (similar to **4**$^+$), and the second order perturbation analysis revealed energetically relevant delocalisation energies between the NBO donor and acceptors of similar orbital character as **4**$^+$ (See main text, **Figure 4**). More specifically, a delocalisation energy of ~111 kcal mol^{-1} from a P-based donor NBO having mixed s/p character (53% *vs*. 47%) to an acceptor NBO of mostly d_{z^2} character. Similar to **4**$^+$, a smaller delocalisation energy (~31 kcal mol^{-1}) of was found between the same donor to an acceptor NBO of mostly s character (90%). In contrast to **4**$^+$, an additional energetically relevant interaction was found between the donor NBO and an acceptor NBO of mostly d character (97%). Both these contributions are higher in energy than the respective contributions in **4**$^+$, which is likely due to better donor-acceptor overlap (the Ti-P distance of the titanocene-based compound is approx. 0.06 Å shorter than those in **4**$^+$).

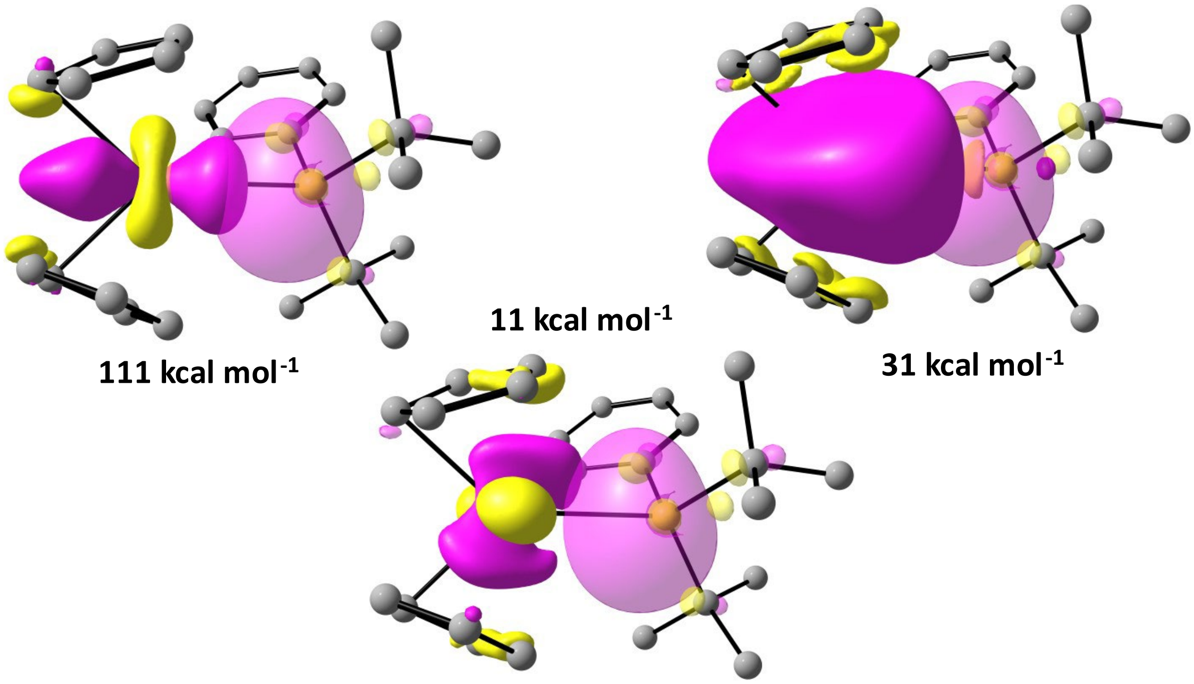

Figure S73: Overlap of the donor (translucent)-acceptor (opaque) NBOs with the respective delocalization energies obtained from second order perturbation analysis.

2.8 Computational Study Of [$^{t\text{-Bu}}$PNNP^{Me}TiCl$_3$]$^+$ and [$^{t\text{-Bu}}$PNN^{Me}TiCl$_3$]$^+$:

In order to investigate if the long Ti-P distance was mainly dominated by steric repulsion between the -*t*Bu substituents on the phosphines and the -Cl ligands on Ti, we ran a gas-phase geometry optimisation of a cation similar to **4**$^+$ with one of the -P*t*Bu$_2$ donors exchanged for a -PMe$_2$. Comparison of the two Ti-P bond lengths of the optimised structure revealed that the (slightly) less electron donating and sterically less encumbered -PMe$_2$ donor has a significantly shorter Ti-P distance of 2.65 Å *vs*. the Ti-P distance of 2.84 Å on the side of the -P*t*Bu$_2$ donor. This confirms the hypothesis that the bond is long mainly due to steric repulsion and not due to unforeseen electronic effects. Additionally, we optimised the geometry of the cation of [$^{t\text{-Bu}}$PNN^{Me}TiCl$_3$] to gauge the effect the absence of the additional phosphine has on Ti-P distance. In absence of the additional phosphine, the Ti-P*t*Bu$_2$ distance contracts to 2.65 Å.

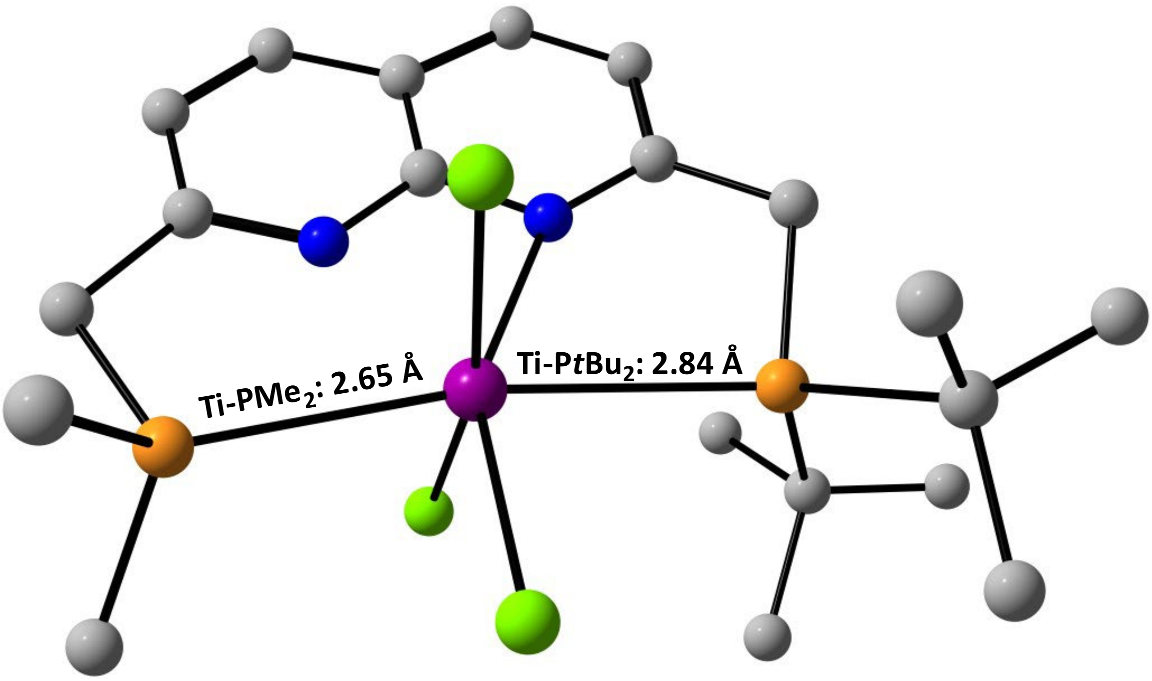

Figure S74: Gas-phase optimised structure of **[^{tBu}PNNP^{Me}TiCl$_3$]$^+$**, hydrogen atoms were omitted for clarity

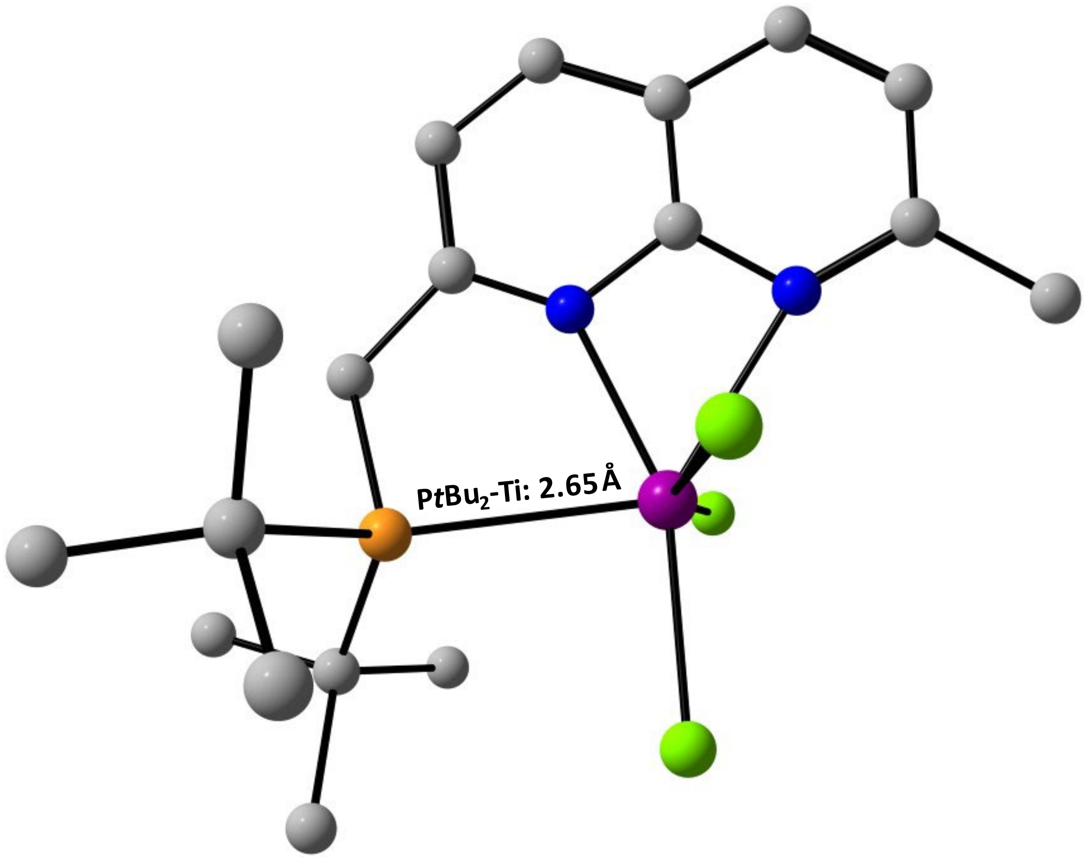

Figure S75: Gas-phase optimised structure of **[^{tBu}PNN^{Me}TiCl$_3$]$^+$**, hydrogen atoms were omitted for clarity.

2.9 XYZ coordinates of [$^{t\text{-}Bu}$PNNP^{Me}TiCl$_3$]$^+$:

1 1

Ti	13.316045000	3.845115000	3.704143000
Cl	14.806631000	5.439084000	3.011436000
Cl	11.703294000	2.218110000	3.804261000
Cl	12.762695000	4.854928000	5.676846000
P	11.393955000	5.404067000	2.745245000
P	15.212921000	2.376432000	5.227093000
N	12.979078000	3.553813000	1.523456000
N	14.602659000	2.484460000	2.502051000
C	11.151744000	4.945625000	0.924758000
C	12.226178000	3.962938000	0.523472000
C	12.493187000	3.451510000	-0.781234000
C	13.534393000	2.559289000	-0.993190000
C	14.350985000	2.142385000	0.098521000
C	13.980267000	2.687411000	1.330482000
C	15.500462000	1.300886000	0.158212000
C	16.164279000	1.124998000	1.362455000
C	15.707167000	1.763084000	2.554923000
C	16.407732000	1.791716000	3.884688000
C	11.744249000	7.202192000	2.752928000
C	9.743899000	5.220734000	3.516901000
C	16.336900000	3.322696000	6.444056000
C	17.701878000	2.642466000	6.678672000
C	15.600326000	3.501054000	7.787830000
C	16.579032000	4.717569000	5.829962000
C	14.561379000	0.739194000	5.964259000
C	14.137948000	-0.167523000	4.785935000
C	15.608101000	-0.020979000	6.800020000
C	13.327639000	1.055427000	6.837225000
H	10.163085000	4.489204000	0.797125000
H	11.175588000	5.838411000	0.290989000
H	11.876977000	3.775609000	-1.612782000
H	13.733530000	2.188691000	-1.994302000
H	15.869681000	0.815984000	-0.740610000
H	17.056683000	0.510331000	1.407004000
H	17.203730000	2.542864000	3.813745000
H	16.889075000	0.840540000	4.124932000
H	18.279631000	3.265137000	7.371115000
H	18.292037000	2.559792000	5.761168000
H	17.619364000	1.651861000	7.124894000
H	15.502764000	2.562459000	8.337509000
H	14.607481000	3.938162000	7.655840000
H	16.183465000	4.184585000	8.414731000
H	15.651010000	5.281474000	5.726896000
H	17.055646000	4.670133000	4.845881000
H	17.249304000	5.278206000	6.490936000
H	13.632907000	-1.050438000	5.192118000
H	14.998082000	-0.529465000	4.213545000
H	13.440752000	0.322928000	4.106259000
H	15.879211000	0.509535000	7.714140000

H	16.522006000	-0.235987000	6.236971000
H	15.179074000	-0.984099000	7.099342000
H	12.895936000	0.112508000	7.191107000
H	12.557781000	1.589420000	6.278769000
H	13.584842000	1.648225000	7.716511000
H	12.670827000	7.391557000	2.207074000
H	11.893092000	7.508869000	3.791680000
H	10.921359000	7.774501000	2.315326000
H	9.436493000	4.174517000	3.457163000
H	8.999557000	5.863914000	3.039241000
H	9.839225000	5.488920000	4.572318000

2.10 XYZ coordinates of [$^{t\text{-}Bu}$PNN^{Me}TiCl$_3$]$^+$:

Ti	13.109628000	3.769898000	4.029950000
Cl	14.367306000	5.599802000	3.678088000
Cl	11.311297000	2.459412000	3.651801000
Cl	12.378434000	4.436718000	6.029884000
P	14.747461000	2.125782000	5.301739000
N	12.781367000	4.021803000	1.679091000
N	14.339343000	2.738006000	2.603044000
C	11.006990000	5.486897000	0.941822000
C	12.117928000	4.528548000	0.646093000
C	12.477830000	4.144935000	-0.682897000
C	13.507212000	3.259205000	-0.922019000
C	14.220469000	2.718423000	0.181598000
C	13.780105000	3.150571000	1.444451000
C	15.320460000	1.820379000	0.186789000
C	15.906510000	1.443440000	1.384142000
C	15.397633000	1.939861000	2.609922000
C	16.002506000	1.717441000	3.967051000
C	15.790193000	2.849426000	6.727834000
C	17.114774000	2.075567000	6.913049000
C	14.986213000	2.828333000	8.042835000
C	16.114750000	4.311559000	6.358053000
C	13.935121000	0.448294000	5.713319000
C	13.556931000	-0.233868000	4.379818000
C	14.897634000	-0.480747000	6.479835000
C	12.663366000	0.693884000	6.551648000
H	10.247903000	4.989716000	1.556201000
H	11.391143000	6.333271000	1.521420000
H	11.921315000	4.571907000	-1.509703000
H	13.775332000	2.980441000	-1.936601000
H	15.711124000	1.442301000	-0.753533000
H	16.762981000	0.778808000	1.395289000
H	16.836351000	2.420078000	4.078128000
H	16.412957000	0.711617000	4.084102000
H	17.654666000	2.534562000	7.748194000
H	17.769360000	2.143638000	6.039901000
H	16.971319000	1.023683000	7.158842000

H	14.831058000	1.815935000	8.421159000
H	14.017986000	3.322853000	7.945628000
H	15.559638000	3.373141000	8.800252000
H	15.218362000	4.931652000	6.325448000
H	16.625461000	4.402400000	5.394300000
H	16.783827000	4.720193000	7.122619000
H	13.000663000	-1.148201000	4.610488000
H	14.436384000	-0.533186000	3.801237000
H	12.914614000	0.388043000	3.755620000
H	15.130008000	-0.111196000	7.479552000
H	15.834893000	-0.657595000	5.943032000
H	14.406554000	-1.452586000	6.599192000
H	12.183095000	-0.272080000	6.740764000
H	11.945467000	1.328729000	6.031393000
H	12.884160000	1.146050000	7.519599000
H	10.537382000	5.862792000	0.031993000

2.11 XYZ coordinates and energies of [4-P]$^+$:

Electronic energy from SP calculation: -3249.0890241400 hartrees
Gibbs thermal correction: 0.5933830000 hartrees

Ti	13.147304000	3.813804000	4.005121000
Cl	14.456013000	5.617753000	3.693569000
Cl	11.341621000	2.502170000	3.642154000
Cl	12.400677000	4.503199000	5.997176000
P	10.837360000	6.974323000	-0.152787000
P	14.763662000	2.150013000	5.292654000
N	12.822572000	4.054125000	1.688599000
N	14.384926000	2.775236000	2.599751000
C	10.986801000	5.450315000	0.972569000
C	12.110712000	4.519467000	0.667633000
C	12.422850000	4.084995000	-0.663583000
C	13.446779000	3.199333000	-0.909710000
C	14.206758000	2.696200000	0.184967000
C	13.811401000	3.171832000	1.445476000
C	15.295865000	1.787778000	0.189294000
C	15.905375000	1.429795000	1.383967000
C	15.426946000	1.954444000	2.607702000
C	16.029400000	1.737403000	3.967353000
C	9.045399000	7.478744000	0.318606000
C	8.140324000	6.576561000	-0.552089000
C	8.808864000	8.943818000	-0.098646000
C	8.648749000	7.301665000	1.797355000
C	12.115398000	8.170279000	0.621330000
C	11.864088000	8.594687000	2.076600000
C	13.478939000	7.448028000	0.536425000
C	12.192381000	9.418725000	-0.285828000
C	15.797529000	2.856366000	6.734067000
C	17.113566000	2.070607000	6.929558000
C	14.977025000	2.835145000	8.038812000

C	16.139983000	4.318138000	6.380122000
C	13.939422000	0.474782000	5.691626000
C	13.566283000	-0.198782000	4.352210000
C	14.889789000	-0.465448000	6.459607000
C	12.663623000	0.722965000	6.523227000
H	10.054648000	4.898154000	0.799339000
H	11.015849000	5.706010000	2.034843000
H	11.835716000	4.494986000	-1.477458000
H	13.676251000	2.885611000	-1.923590000
H	15.658828000	1.378198000	-0.748963000
H	16.751331000	0.751798000	1.390253000
H	16.860769000	2.442265000	4.082594000
H	16.439167000	0.732215000	4.090841000
H	8.266046000	5.510834000	-0.327318000
H	8.330319000	6.721495000	-1.619621000
H	7.088766000	6.819560000	-0.359267000
H	9.133803000	9.138619000	-1.125970000
H	9.318860000	9.646268000	0.565318000
H	7.736865000	9.166334000	-0.044206000
H	7.614693000	7.640626000	1.933384000
H	9.274638000	7.884172000	2.475331000
H	8.680887000	6.255820000	2.117192000
H	12.710416000	9.193462000	2.435294000
H	11.771088000	7.737376000	2.751453000
H	10.966953000	9.210349000	2.175176000
H	13.674370000	7.053837000	-0.467391000
H	13.560457000	6.633562000	1.259051000
H	14.279241000	8.158963000	0.772715000
H	11.280908000	10.017706000	-0.258255000
H	12.388929000	9.146829000	-1.327948000
H	13.014518000	10.060291000	0.052984000
H	17.649101000	2.521191000	7.772127000
H	17.776781000	2.137127000	6.062790000
H	16.959142000	1.018850000	7.168830000
H	14.809713000	1.822287000	8.410611000
H	14.013451000	3.336898000	7.930302000
H	15.544129000	3.372689000	8.806174000
H	15.249430000	4.946057000	6.340025000
H	16.664609000	4.411776000	5.424293000
H	16.802056000	4.714428000	7.157289000
H	13.002485000	-1.110650000	4.574420000
H	14.448134000	-0.500503000	3.778622000
H	12.932829000	0.430381000	3.726389000
H	15.117513000	-0.103309000	7.463107000
H	15.829684000	-0.645747000	5.928605000
H	14.391092000	-1.434528000	6.569859000
H	12.171936000	-0.240272000	6.696646000
H	11.956377000	1.371745000	6.005877000
H	12.881869000	1.160477000	7.498397000

2.12 XYZ coordinates and energies of $[4\text{-}2P]^+$:

Electronic energy from SP calculation: -3249.0663923300 hartrees
Gibbs thermal correction: 0.5884490000 hartrees

Ti	14.118203000	4.559632000	3.069659000
Cl	15.975793000	5.584309000	3.555994000
Cl	13.628665000	3.136050000	4.615728000
Cl	12.675854000	6.189383000	3.367491000
P	9.622228000	4.142067000	0.162686000
P	17.821615000	0.514149000	3.297404000
N	13.385402000	4.132580000	1.060542000
N	15.240166000	3.180615000	1.836473000
C	11.231432000	5.156161000	0.413973000
C	12.453239000	4.366086000	0.118712000
C	12.641329000	3.817296000	-1.186557000
C	13.738607000	3.051334000	-1.505460000
C	14.705240000	2.773966000	-0.504337000
C	14.449294000	3.339251000	0.752156000
C	15.895527000	2.007250000	-0.598407000
C	16.698174000	1.849780000	0.511659000
C	16.355591000	2.442705000	1.760407000
C	17.190007000	2.284104000	2.985159000
C	9.320161000	3.431638000	1.915436000
C	10.603638000	2.664534000	2.299858000
C	8.173074000	2.403939000	1.792765000
C	9.002629000	4.443751000	3.027421000
C	8.426632000	5.599939000	-0.195719000
C	8.621140000	6.877533000	0.642752000
C	8.657977000	5.938788000	-1.686634000
C	6.978112000	5.095223000	-0.035110000
C	16.339320000	-0.278690000	4.212057000
C	15.990474000	0.304707000	5.590236000
C	16.641060000	-1.788750000	4.342879000
C	15.118592000	-0.124500000	3.276216000
C	19.238112000	0.936365000	4.519598000
C	20.419673000	1.366176000	3.619132000
C	18.956279000	2.056888000	5.539233000
C	19.646040000	-0.348195000	5.268883000
H	11.160830000	5.952368000	-0.333465000
H	11.275199000	5.615608000	1.396787000
H	11.873185000	4.013350000	-1.925390000
H	13.862891000	2.652237000	-2.507727000
H	16.164625000	1.543382000	-1.542807000
H	17.596919000	1.245953000	0.467650000
H	16.666537000	2.705382000	3.844931000
H	18.103298000	2.873901000	2.840960000
H	11.416032000	3.347150000	2.557124000
H	10.946903000	1.995014000	1.502298000
H	10.411846000	2.051754000	3.188005000

H	8.392857000	1.643151000	1.037153000
H	7.219008000	2.868608000	1.537196000
H	8.040197000	1.894086000	2.754374000
H	8.948246000	3.921341000	3.990727000
H	8.039488000	4.934590000	2.873331000
H	9.773017000	5.215428000	3.121748000
H	7.894987000	7.631961000	0.317127000
H	9.613445000	7.319935000	0.512543000
H	8.461795000	6.711542000	1.708544000
H	8.470029000	5.076486000	-2.333285000
H	9.674907000	6.298347000	-1.885118000
H	7.975892000	6.742007000	-1.988272000
H	6.708726000	4.945704000	1.013257000
H	6.804701000	4.158690000	-0.575381000
H	6.288630000	5.843190000	-0.443101000
H	15.066929000	-0.157207000	5.961056000
H	15.819697000	1.384963000	5.555483000
H	16.769721000	0.104480000	6.328787000
H	17.484501000	-1.992327000	5.005040000
H	16.856916000	-2.241488000	3.369959000
H	15.765695000	-2.297970000	4.763188000
H	15.337222000	-0.456154000	2.254794000
H	14.754570000	0.905491000	3.241709000
H	14.294276000	-0.741140000	3.652830000
H	21.290265000	1.594667000	4.244769000
H	20.201080000	2.270417000	3.038749000
H	20.705332000	0.575467000	2.919242000
H	18.121322000	1.825230000	6.201711000
H	18.754539000	3.019465000	5.059330000
H	19.843133000	2.198761000	6.168321000
H	20.586743000	-0.171118000	5.802756000
H	19.808279000	-1.189477000	4.587025000
H	18.902896000	-0.644182000	6.013431000

2.13 Structural description of [Int-A]$^+$:

The coordination of diphenylacetaldehyde to [**4**-**P**]$^+$ causes a slight elongation of the C=O bond length from 1.21 Å to 1.23 Å and in parallel the Ti-P bond length is increased to 2.72 Å. Both observations can be rationalised when the resonance form of the bound alkoxide/carbocation zwitterion (**Figure S76**) is considered. Upon coordination of the aldehyde, the bound zwitterion resonance form will have a higher contribution, which is consistent with the elongation of the C-O distance. Moreover, the larger contribution of this resonance form results in a higher anionic character on the O-donor (larger X-type ligand character) and hence higher electron density on the Ti centre. This alleviates the need of stabilisation by the P-donor, which in turn is expressed in a larger Ti-P distance. Based on the C=O bond length, the structure of [**Int**-**A**]$^+$ is best described as the left resonance structure of **Figure S76**, but with a minor contribution of the alkoxide/carbocation resonance structure. These trends are also observed for [**Int**-**A**-**P**]$^+$: The C=O bond is elongated to 1.25 Å and the Ti-O bond is contracted to 2.00 Å compared to the Ti-O bond length of 2.14 Å found for [**Int**-**A**]$^+$. Both observations are consistent with an even larger contribution of the alkoxide/carbocation resonance form in [**Int**-**A**-**P**]$^+$, which is unsurprising considering the more electron-poor Ti centre in this compound.

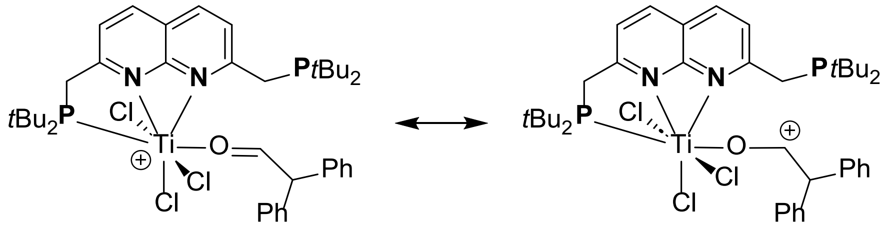

Figure S76: The two resonance forms of [**Int**-**A**]$^+$.

2.14 XYZ coordinates and energies of [Int-A]$^+$:

Electronic energy from SP calculation: -3865.2450125600 hartrees
Gibbs thermal correction: 0.7993470000 hartrees

Ti	13.667324000	3.980104000	2.985848000
Cl	13.403760000	6.260708000	2.954925000
Cl	14.600896000	1.936687000	3.021061000
Cl	11.730948000	3.550190000	4.150260000
P	11.936275000	3.439678000	0.959163000
P	19.150364000	4.482991000	5.056750000
N	14.691508000	4.168883000	1.054390000
N	16.084936000	4.798115000	2.700039000
C	13.133137000	3.098473000	-0.428240000
C	14.394390000	3.877583000	-0.210548000
C	15.278411000	4.231444000	-1.256397000
C	16.475976000	4.855019000	-0.966023000
C	16.829150000	5.099113000	0.381826000
C	15.896542000	4.707610000	1.366348000
C	18.030400000	5.683484000	0.864329000
C	18.221561000	5.781722000	2.218805000
C	17.235833000	5.300286000	3.142207000
C	17.511778000	5.348079000	4.610427000
C	10.935235000	4.945144000	0.336072000
C	11.932144000	5.989849000	-0.214725000
C	10.150649000	5.547227000	1.520621000
C	9.962212000	4.585754000	-0.804676000
C	10.872996000	1.845205000	1.030244000
C	10.585567000	1.288865000	-0.383253000
C	11.671180000	0.782146000	1.812290000
C	9.537462000	2.101814000	1.758550000
C	18.617448000	2.638857000	5.092201000
C	17.258443000	2.318912000	5.741791000
C	19.728152000	1.825292000	5.786817000
C	18.558294000	2.202517000	3.608281000
C	19.466765000	5.132202000	6.837382000
C	19.345522000	6.672255000	6.799281000

C	18.551752000	4.596981000	7.949004000
C	20.937555000	4.783257000	7.164036000
H	13.391388000	2.034473000	-0.365470000
H	12.703744000	3.272405000	-1.417460000
H	15.002597000	4.000562000	-2.279068000
H	17.157854000	5.140294000	-1.761974000
H	18.785391000	6.029721000	0.164790000
H	19.139458000	6.192850000	2.622074000
H	16.659078000	4.965647000	5.163148000
H	17.658562000	6.393949000	4.893718000
H	12.407368000	5.655149000	-1.142070000
H	12.706008000	6.258033000	0.503356000
H	11.374974000	6.902277000	-0.452674000
H	10.810754000	5.850202000	2.333627000
H	9.410772000	4.852953000	1.922702000
H	9.614925000	6.435645000	1.168084000
H	9.498710000	5.512984000	-1.159898000
H	9.156231000	3.925068000	-0.483778000
H	10.468677000	4.131383000	-1.661903000
H	9.979616000	0.383133000	-0.269885000
H	11.495391000	0.993940000	-0.913011000
H	10.023984000	1.976004000	-1.015156000
H	11.849723000	1.081779000	2.844986000
H	12.637592000	0.559586000	1.351116000
H	11.090456000	-0.146846000	1.819967000
H	8.875715000	2.765706000	1.199352000
H	9.683932000	2.511178000	2.758310000
H	9.018235000	1.142867000	1.863747000
H	17.071269000	1.240154000	5.668868000
H	16.429233000	2.814979000	5.234254000
H	17.229756000	2.580883000	6.801176000
H	19.758089000	2.003004000	6.864821000
H	20.718143000	2.045616000	5.373829000
H	19.539467000	0.755182000	5.639949000
H	19.495403000	2.412586000	3.083595000
H	17.738574000	2.686323000	3.070114000
H	18.375871000	1.122377000	3.555998000
H	19.783515000	7.088808000	7.713564000
H	18.305802000	7.009898000	6.766694000
H	19.886711000	7.108983000	5.951762000
H	18.666756000	3.521547000	8.100867000
H	17.501270000	4.814588000	7.746300000
H	18.802482000	5.088772000	8.897257000
H	21.200125000	5.200286000	8.143712000
H	21.623357000	5.206882000	6.424038000
H	21.114794000	3.706728000	7.210162000
O	14.418937000	4.228859000	4.977839000
C	14.678442000	5.009720000	7.219294000
C	13.977926000	4.917781000	5.901117000
H	13.033418000	5.463889000	5.774390000
C	13.659941000	4.469216000	8.223475000

C	13.681100000	3.106180000	8.548467000
C	12.659008000	5.292836000	8.757802000
C	12.728141000	2.577080000	9.418927000
C	11.706485000	4.760650000	9.625641000
C	11.740015000	3.403616000	9.958088000
H	14.447860000	2.463242000	8.123572000
H	12.642552000	6.350332000	8.511198000
H	12.757989000	1.522675000	9.675413000
H	10.939930000	5.405257000	10.044533000
H	10.998400000	2.992447000	10.636006000
C	15.195244000	6.422961000	7.474048000
C	15.640588000	6.770571000	8.756053000
C	15.311310000	7.354988000	6.434652000
C	16.200779000	8.025000000	8.991384000
C	15.863738000	8.616070000	6.674225000
C	16.313520000	8.952013000	7.951310000
H	15.546525000	6.054821000	9.567086000
H	14.979405000	7.109627000	5.428201000
H	16.548062000	8.279770000	9.987972000
H	15.940796000	9.331930000	5.861392000
H	16.746648000	9.929853000	8.137388000
H	15.526670000	4.318668000	7.175473000

2.15 XYZ coordinates and energies of [Int-A-P]$^+$:

Electronic energy from SP calculation: -3865.2252688100 hartrees
Gibbs thermal correction: 0.7930040000 hartrees

Ti	13.571454000	3.946855000	3.174487000
Cl	14.183918000	6.035453000	2.533041000
Cl	13.335706000	1.811418000	3.953203000
Cl	11.463692000	4.153901000	2.543124000
P	12.638082000	1.070171000	-1.311216000
P	17.873198000	3.749917000	6.713904000
N	14.629430000	3.088583000	1.506586000
N	15.804203000	3.637641000	3.331910000
C	13.125233000	2.642544000	-0.357853000
C	14.485194000	2.648123000	0.251228000
C	15.639396000	2.229547000	-0.472172000
C	16.892459000	2.272522000	0.095557000
C	17.043598000	2.743326000	1.425424000
C	15.862738000	3.127214000	2.078943000
C	18.240345000	2.914313000	2.167904000
C	18.180076000	3.463128000	3.428340000
C	16.934392000	3.853397000	4.005176000
C	16.880482000	4.574324000	5.315685000
C	12.170398000	-0.131528000	0.104108000
C	13.460244000	-0.331370000	0.932153000
C	11.820079000	-1.494071000	-0.535479000

C	11.036252000	0.316139000	1.040013000
C	11.066420000	1.776789000	-2.162745000
C	10.213287000	2.751788000	-1.327178000
C	11.595358000	2.520751000	-3.410934000
C	10.178757000	0.610163000	-2.639152000
C	16.515446000	2.732140000	7.592756000
C	15.412409000	3.540622000	8.293059000
C	17.206209000	1.798829000	8.610340000
C	15.875243000	1.855754000	6.496730000
C	18.325822000	5.304156000	7.739922000
C	19.537899000	5.917141000	6.999858000
C	17.238013000	6.383198000	7.885503000
C	18.792297000	4.853112000	9.138414000
H	12.386562000	2.908751000	0.395829000
H	13.106402000	3.429216000	-1.121980000
H	15.494863000	1.862684000	-1.481697000
H	17.765543000	1.956891000	-0.468146000
H	19.193361000	2.630760000	1.731156000
H	19.080086000	3.612654000	4.013078000
H	15.845509000	4.790757000	5.584816000
H	17.372059000	5.540660000	5.155567000
H	13.708529000	0.546638000	1.530822000
H	14.318813000	-0.585897000	0.300962000
H	13.311403000	-1.157344000	1.637438000
H	12.614004000	-1.840718000	-1.205002000
H	10.887161000	-1.469296000	-1.100090000
H	11.701554000	-2.242936000	0.256718000
H	10.930694000	-0.407585000	1.857951000
H	10.074351000	0.363185000	0.523529000
H	11.232218000	1.289778000	1.499369000
H	9.331275000	3.044730000	-1.909539000
H	10.749281000	3.673557000	-1.082882000
H	9.858632000	2.309217000	-0.394865000
H	12.142038000	1.850401000	-4.080570000
H	12.257111000	3.356337000	-3.154572000
H	10.751634000	2.943289000	-3.969392000
H	9.657146000	0.127772000	-1.808724000
H	10.750351000	-0.151405000	-3.179607000
H	9.413328000	0.994887000	-3.323274000
H	14.608801000	2.865395000	8.617781000
H	14.976870000	4.298296000	7.636056000
H	15.779441000	4.056357000	9.183373000
H	17.680641000	2.346338000	9.427332000
H	17.969604000	1.179620000	8.129540000
H	16.460897000	1.127334000	9.053832000
H	16.624723000	1.301979000	5.920400000
H	15.275047000	2.437707000	5.795644000
H	15.207345000	1.117972000	6.957260000
H	19.895678000	6.796118000	7.548800000
H	19.284567000	6.256286000	5.988348000
H	20.367289000	5.207790000	6.921646000

H	16.362886000	6.039473000	8.436864000
H	16.890040000	6.769142000	6.924144000
H	17.649898000	7.237640000	8.436565000
H	19.255192000	5.700353000	9.657812000
H	19.536432000	4.051368000	9.087004000
H	17.959111000	4.510886000	9.757843000
O	13.429035000	4.583865000	5.064217000
C	12.381011000	5.174206000	7.142692000
C	12.779673000	4.231252000	6.070660000
H	12.404323000	3.199805000	6.113994000
C	10.917949000	5.375210000	6.658690000
C	9.872294000	4.724293000	7.330205000
C	10.639452000	6.154927000	5.525531000
C	8.557124000	4.892198000	6.901187000
C	9.322113000	6.319669000	5.101106000
C	8.280820000	5.689852000	5.786820000
H	10.084757000	4.109348000	8.200502000
H	11.443543000	6.639600000	4.983561000
H	7.749222000	4.404494000	7.437359000
H	9.112338000	6.932455000	4.230423000
H	7.255966000	5.816687000	5.452365000
C	13.207423000	6.425590000	7.366427000
C	13.763047000	7.194532000	6.331687000
C	13.389785000	6.843177000	8.692540000
C	14.476651000	8.357437000	6.627865000
C	14.104046000	8.004584000	8.984797000
C	14.648290000	8.767793000	7.950476000
H	13.658349000	6.895969000	5.296275000
H	12.965093000	6.257879000	9.504256000
H	14.898469000	8.942536000	5.816155000
H	14.232997000	8.311579000	10.018033000
H	15.203074000	9.673685000	8.173967000
H	12.326490000	4.599313000	8.072000000

2.16 XYZ coordinates and energies of [Int-B]$^+$:

Electronic energy from SP calculation: -3865.2413692100 hartrees
Gibbs thermal correction: 0.8017070000 hartrees

Ti	14.327152000	3.655416000	3.495581000
Cl	14.500381000	5.774170000	2.688068000
Cl	14.801842000	1.502838000	4.159717000
Cl	12.224298000	3.859769000	4.310912000
P	12.816012000	2.699573000	1.361560000
P	17.765476000	4.119370000	6.778828000
N	15.608386000	3.002127000	1.783794000
N	16.808412000	3.986567000	3.383234000
C	14.104740000	1.729859000	0.418007000
C	15.453261000	2.344242000	0.641560000
C	16.543721000	2.202982000	-0.256438000
C	17.777138000	2.735395000	0.059403000

C	17.958036000	3.387612000	1.305345000
C	16.828183000	3.457199000	2.145854000
C	19.135358000	3.995353000	1.812300000
C	19.103269000	4.572067000	3.061454000
C	17.916330000	4.539922000	3.858797000
C	17.872705000	5.187049000	5.204006000
C	12.256028000	4.034158000	0.110142000
C	13.521927000	4.674433000	-0.503261000
C	11.448423000	5.109700000	0.867888000
C	11.411906000	3.471380000	-1.049969000
C	11.436438000	1.404848000	1.672885000
C	11.234888000	0.462814000	0.464743000
C	11.860574000	0.546719000	2.882649000
C	10.102054000	2.103186000	2.008390000
C	18.549528000	5.356432000	8.028445000
C	20.015137000	5.724689000	7.733571000
C	18.463577000	4.802158000	9.466875000
C	17.671740000	6.629674000	7.967556000
C	18.880162000	2.580877000	6.535358000
C	18.032505000	1.572067000	5.728005000
C	20.215368000	2.813088000	5.804328000
C	19.171117000	1.960267000	7.918474000
H	14.126198000	0.727879000	0.863932000
H	13.873778000	1.612563000	-0.643264000
H	16.387975000	1.675787000	-1.190976000
H	18.610219000	2.649273000	-0.632177000
H	20.042272000	4.009989000	1.215142000
H	19.984716000	5.060364000	3.461319000
H	16.952328000	5.779114000	5.245888000
H	18.721807000	5.865793000	5.298212000
H	14.054657000	3.981024000	-1.161962000
H	14.213079000	5.050666000	0.250308000
H	13.213077000	5.525885000	-1.119318000
H	12.021543000	5.549341000	1.684660000
H	10.518155000	4.714213000	1.279156000
H	11.186252000	5.910080000	0.166741000
H	11.211926000	4.286373000	-1.754963000
H	10.446360000	3.084718000	-0.722149000
H	11.933000000	2.685810000	-1.606206000
H	10.441312000	-0.248913000	0.719052000
H	12.127647000	-0.127884000	0.240864000
H	10.926644000	0.979900000	-0.443150000
H	11.969226000	1.142200000	3.789148000
H	12.801585000	0.017149000	2.711003000
H	11.085998000	-0.208822000	3.056615000
H	9.681364000	2.636773000	1.154064000
H	10.200706000	2.798220000	2.843410000
H	9.376585000	1.334520000	2.297392000
H	20.324354000	6.527115000	8.413842000
H	20.177994000	6.089893000	6.714893000
H	20.685920000	4.880069000	7.906863000

H	19.152268000	3.976193000	9.640363000
H	17.459574000	4.472246000	9.725138000
H	18.739308000	5.602631000	10.163250000
H	16.624039000	6.407911000	8.191248000
H	17.720955000	7.145253000	7.004843000
H	18.022570000	7.337144000	8.727005000
H	18.623661000	0.665172000	5.553926000
H	17.719681000	1.955807000	4.755215000
H	17.128571000	1.275689000	6.266969000
H	20.827674000	3.585331000	6.271694000
H	20.067681000	3.075665000	4.756341000
H	20.793678000	1.881191000	5.820847000
H	19.545132000	0.940141000	7.774022000
H	18.280389000	1.903283000	8.550853000
H	19.942708000	2.513665000	8.457846000
O	14.821807000	4.301411000	5.390638000
C	14.465451000	4.343040000	7.761121000
C	14.991885000	3.718561000	6.490180000
H	15.189683000	2.643771000	6.473440000
C	15.008197000	3.797219000	9.068692000
C	15.545821000	2.511125000	9.207918000
C	14.895582000	4.604196000	10.209208000
C	15.981063000	2.053457000	10.454160000
C	15.326183000	4.148466000	11.454606000
C	15.876992000	2.870774000	11.580235000
H	15.637509000	1.852204000	8.350710000
H	14.462357000	5.596909000	10.117869000
H	16.399757000	1.055253000	10.542009000
H	15.234490000	4.791096000	12.324857000
H	16.216245000	2.514105000	12.547663000
C	12.942779000	4.141361000	7.670164000
C	12.108577000	5.230728000	7.401447000
C	12.384297000	2.865907000	7.824132000
C	10.728489000	5.051672000	7.304797000
C	11.006492000	2.685477000	7.710504000
C	10.175484000	3.778499000	7.453441000
H	12.538541000	6.218621000	7.263038000
H	13.023215000	2.018026000	8.052421000
H	10.087855000	5.905637000	7.107125000
H	10.581670000	1.693775000	7.833106000
H	9.101586000	3.639069000	7.374975000
H	14.657378000	5.417550000	7.697373000

2.17 XYZ coordinates and energies of [Int-C]$^+$:

Electronic energy from SP calculation: -3865.2526974300 hartrees
Gibbs thermal correction: 0.8082020000 hartrees

Ti	14.516181000	3.880832000	3.352645000
Cl	14.956977000	5.789792000	2.130633000
Cl	14.665981000	1.685109000	4.150074000
Cl	12.419720000	4.515214000	3.882111000
P	12.887780000	2.736803000	1.151437000
P	17.524541000	4.503499000	6.344368000
N	15.683792000	2.740500000	1.627675000
N	16.943794000	3.849961000	3.057559000
C	14.061359000	1.463517000	0.423190000
C	15.474541000	1.935767000	0.600595000
C	16.569783000	1.536913000	-0.224903000
C	17.850307000	1.974886000	0.042710000
C	18.080018000	2.806129000	1.174807000
C	16.937091000	3.118044000	1.936599000
C	19.292467000	3.381849000	1.636572000
C	19.282124000	4.176734000	2.769812000
C	18.066099000	4.387451000	3.480290000
C	17.957106000	5.264604000	4.694795000
C	12.552600000	3.897662000	-0.333118000
C	13.917422000	4.277476000	-0.951308000
C	11.876130000	5.178863000	0.202046000
C	11.689204000	3.274218000	-1.445290000
C	11.336165000	1.696808000	1.573315000
C	11.027477000	0.598422000	0.534294000
C	11.605049000	1.016726000	2.933143000
C	10.109815000	2.619672000	1.732639000
C	18.111083000	5.889323000	7.545079000
C	19.471059000	6.492697000	7.106131000
C	18.266054000	5.368518000	8.988422000
C	17.071366000	7.030248000	7.483455000
C	18.498912000	2.874347000	6.538532000
C	18.187674000	1.916677000	5.361835000
C	20.013672000	3.164890000	6.550363000
C	18.098795000	2.144606000	7.837925000
H	13.944279000	0.558539000	1.032323000
H	13.844095000	1.193478000	-0.613188000
H	16.374316000	0.888782000	-1.072328000
H	18.677851000	1.687522000	-0.599321000
H	20.218364000	3.206708000	1.096484000
H	20.197224000	4.639682000	3.123780000
H	17.149745000	5.982744000	4.508619000
H	18.886414000	5.811456000	4.836560000
H	14.391448000	3.429913000	-1.457567000
H	14.609964000	4.685847000	-0.214457000
H	13.752277000	5.049597000	-1.711092000
H	12.477814000	5.659995000	0.974951000

H	10.885122000	4.983557000	0.615213000
H	11.754618000	5.887310000	-0.625809000
H	11.626882000	3.982819000	-2.279761000
H	10.668344000	3.068796000	-1.119802000
H	12.120556000	2.348321000	-1.839797000
H	10.137949000	0.048623000	0.863915000
H	11.837406000	-0.132208000	0.449126000
H	10.815691000	0.992482000	-0.459525000
H	11.779472000	1.744443000	3.727472000
H	12.464652000	0.341751000	2.900364000
H	10.726665000	0.417622000	3.201695000
H	9.786593000	3.055106000	0.784973000
H	10.297035000	3.426674000	2.444144000
H	9.273774000	2.023275000	2.115902000
H	19.810728000	7.139367000	7.921460000
H	19.382966000	7.129330000	6.222825000
H	20.254162000	5.754504000	6.935591000
H	19.148796000	4.732799000	9.092378000
H	17.393721000	4.829891000	9.350934000
H	18.409681000	6.232543000	9.645457000
H	16.141344000	6.766925000	7.976680000
H	16.853261000	7.350604000	6.459923000
H	17.486272000	7.895255000	8.011479000
H	18.545388000	0.922390000	5.649327000
H	18.714809000	2.195514000	4.450604000
H	17.124074000	1.828255000	5.133829000
H	20.335046000	3.685155000	7.454497000
H	20.337083000	3.737015000	5.675331000
H	20.541950000	2.206130000	6.521371000
H	18.800699000	1.316569000	7.983081000
H	17.103258000	1.709054000	7.753188000
H	18.126263000	2.764843000	8.731582000
O	15.157267000	4.483718000	4.996513000
C	14.612959000	4.647091000	7.323677000
C	15.573798000	4.077975000	6.233700000
H	15.614419000	2.981386000	6.291155000
C	14.966208000	4.386231000	8.780258000
C	15.280114000	3.103620000	9.247334000
C	14.858797000	5.423513000	9.719185000
C	15.565631000	2.879086000	10.594786000
C	15.129820000	5.203160000	11.069555000
C	15.505864000	3.932366000	11.509641000
H	15.271875000	2.264760000	8.561679000
H	14.547343000	6.412378000	9.393551000
H	15.817650000	1.877901000	10.931678000
H	15.044118000	6.022451000	11.776853000
H	15.724260000	3.758711000	12.558651000
C	13.184592000	4.141261000	7.092565000
C	12.131715000	5.059091000	7.166079000
C	12.888108000	2.788379000	6.898242000
C	10.808758000	4.639073000	7.033222000

C	11.567138000	2.367001000	6.750258000
C	10.522545000	3.290342000	6.817779000
H	12.347522000	6.113908000	7.314624000
H	13.679962000	2.051861000	6.827576000
H	10.004885000	5.366981000	7.086448000
H	11.357262000	1.315251000	6.579941000
H	9.494117000	2.961220000	6.703968000
H	14.588850000	5.725593000	7.154131000

2.18 XYZ coordinates and energies of 5^+:

Electronic energy from SP calculation: -3865.2652887900 hartrees
Gibbs thermal correction: 0.8025020000 hartrees

Ti	14.516444000	4.446389000	3.094609000
Cl	16.288688000	5.649388000	2.250462000
Cl	13.106784000	2.695957000	3.809452000
Cl	12.914473000	5.641841000	2.133639000
P	12.386377000	2.027164000	-1.466715000
P	16.375878000	3.957152000	6.708723000
N	14.960323000	2.865205000	1.464350000
N	16.107990000	2.925916000	3.360712000
C	13.658441000	3.104049000	-0.555572000
C	14.686720000	2.391600000	0.253029000
C	15.409631000	1.253287000	-0.240644000
C	16.390312000	0.647559000	0.506777000
C	16.699644000	1.167292000	1.797010000
C	15.926123000	2.269360000	2.200550000
C	17.710844000	0.765953000	2.702578000
C	17.924602000	1.490922000	3.865411000
C	17.111337000	2.613709000	4.163410000
C	17.451210000	3.626740000	5.226777000
C	11.197285000	1.512206000	-0.057262000
C	12.047108000	0.670716000	0.921720000
C	10.129586000	0.572264000	-0.661158000
C	10.517370000	2.651743000	0.718797000
C	11.614429000	3.421485000	-2.542725000
C	11.471461000	4.802866000	-1.873328000
C	12.560265000	3.552075000	-3.759043000
C	10.234723000	2.964347000	-3.055217000
C	17.165248000	5.531917000	7.417854000
C	18.704774000	5.413436000	7.484325000
C	16.639639000	5.826109000	8.840562000
C	16.808185000	6.697350000	6.459727000
C	16.449869000	2.379593000	7.766133000
C	15.579469000	1.315125000	7.055867000
C	17.904764000	1.862788000	7.833971000
C	15.932284000	2.596455000	9.202261000
H	13.194562000	3.869229000	0.066883000
H	14.186265000	3.621644000	-1.366737000

H	15.148340000	0.880418000	-1.224576000
H	16.936171000	-0.207202000	0.118582000
H	18.345157000	-0.082813000	2.464252000
H	18.740601000	1.228239000	4.529423000
H	17.500244000	4.590686000	4.708881000
H	18.440871000	3.415720000	5.632595000
H	12.738035000	1.283418000	1.502332000
H	12.609701000	-0.115475000	0.405544000
H	11.383773000	0.182326000	1.645429000
H	10.586464000	-0.249684000	-1.222242000
H	9.434773000	1.089909000	-1.323606000
H	9.538201000	0.132845000	0.151201000
H	9.941229000	2.231376000	1.552927000
H	9.818290000	3.211230000	0.091882000
H	11.237120000	3.353159000	1.150529000
H	10.978710000	5.489441000	-2.572587000
H	12.437768000	5.248751000	-1.621679000
H	10.868899000	4.771072000	-0.964028000
H	12.633202000	2.614567000	-4.318212000
H	13.574014000	3.855582000	-3.473118000
H	12.177474000	4.323113000	-4.438360000
H	9.477519000	2.992219000	-2.267688000
H	10.262575000	1.953491000	-3.475135000
H	9.901517000	3.642700000	-3.849536000
H	19.086618000	6.332200000	7.941145000
H	19.169245000	5.333098000	6.498954000
H	19.041779000	4.582357000	8.107511000
H	17.114249000	5.183702000	9.583998000
H	15.560789000	5.715551000	8.944606000
H	16.895325000	6.860323000	9.091586000
H	15.740177000	6.903612000	6.413948000
H	17.149152000	6.533563000	5.434775000
H	17.308812000	7.596344000	6.833735000
H	15.761184000	0.352915000	7.545749000
H	15.823849000	1.193824000	5.997105000
H	14.515836000	1.531160000	7.148219000
H	18.598168000	2.589252000	8.262395000
H	18.284224000	1.541361000	6.861345000
H	17.914602000	0.984395000	8.486956000
H	15.819697000	1.612359000	9.668505000
H	14.961790000	3.088481000	9.239329000
H	16.640885000	3.163686000	9.807794000
O	14.804144000	4.937166000	4.817492000
C	13.638859000	5.155626000	6.955085000
C	14.614781000	4.321918000	6.049365000
H	14.188405000	3.327731000	5.889463000
C	13.069909000	4.332671000	8.099596000
C	13.015031000	4.891790000	9.384545000
C	12.554869000	3.039387000	7.914681000
C	12.502384000	4.169041000	10.462907000
C	12.040982000	2.314436000	8.991212000

C	12.023290000	2.871826000	10.271043000
H	13.365849000	5.908611000	9.541731000
H	12.537686000	2.593435000	6.924557000
H	12.474222000	4.621013000	11.449659000
H	11.648743000	1.315425000	8.826273000
H	11.626775000	2.306102000	11.108286000
C	12.560603000	5.818248000	6.092374000
C	12.722483000	7.162616000	5.733429000
C	11.425787000	5.135090000	5.641096000
C	11.777266000	7.812170000	4.940172000
C	10.478833000	5.782241000	4.847670000
C	10.650170000	7.120918000	4.494044000
H	13.593936000	7.712958000	6.078651000
H	11.269354000	4.096495000	5.901724000
H	11.921281000	8.854825000	4.674167000
H	9.606518000	5.234674000	4.504421000
H	9.912004000	7.621748000	3.875436000
H	14.205790000	5.977582000	7.393304000

2.19 XYZ coordinates and energies of diphenylacetaldehyde:

Electronic energy from SP calculation: -616.1205337980 hartrees
Gibbs thermal correction: 0.1786560000 hartrees

O	-3.560593000	1.017054000	-1.890275000
C	-2.377695000	1.032206000	-1.638243000
C	-1.804226000	1.146958000	-0.222465000
H	-1.611450000	0.974200000	-2.442412000
C	-1.241693000	2.561703000	-0.030451000
C	-2.000994000	3.657800000	-0.467855000
C	-0.022344000	2.804176000	0.612647000
C	0.430487000	4.110436000	0.807009000
C	-1.546861000	4.962060000	-0.276428000
C	-0.326208000	5.193636000	0.359945000
H	-2.148439000	5.796774000	-0.624284000
H	0.029745000	6.208787000	0.508589000
H	0.574759000	1.971751000	0.968008000
H	1.378109000	4.278703000	1.310656000
H	-2.957496000	3.487822000	-0.953826000
C	-0.829968000	0.003041000	-0.007612000
H	-2.659633000	1.024409000	0.451694000
C	0.362074000	-0.084518000	-0.744394000
C	1.221026000	-1.170869000	-0.582328000
C	-1.140829000	-1.026810000	0.888437000
C	-0.280592000	-2.112671000	1.055100000
C	0.902377000	-2.188771000	0.318781000
H	-2.063910000	-0.976128000	1.459607000
H	-0.536331000	-2.899150000	1.759026000
H	2.140419000	-1.220724000	-1.158322000
H	1.572198000	-3.033839000	0.446813000
H	0.626778000	0.714056000	-1.431387000

2.20 Potential Energy Scan of Phosphine Dissociation from 4⁺:

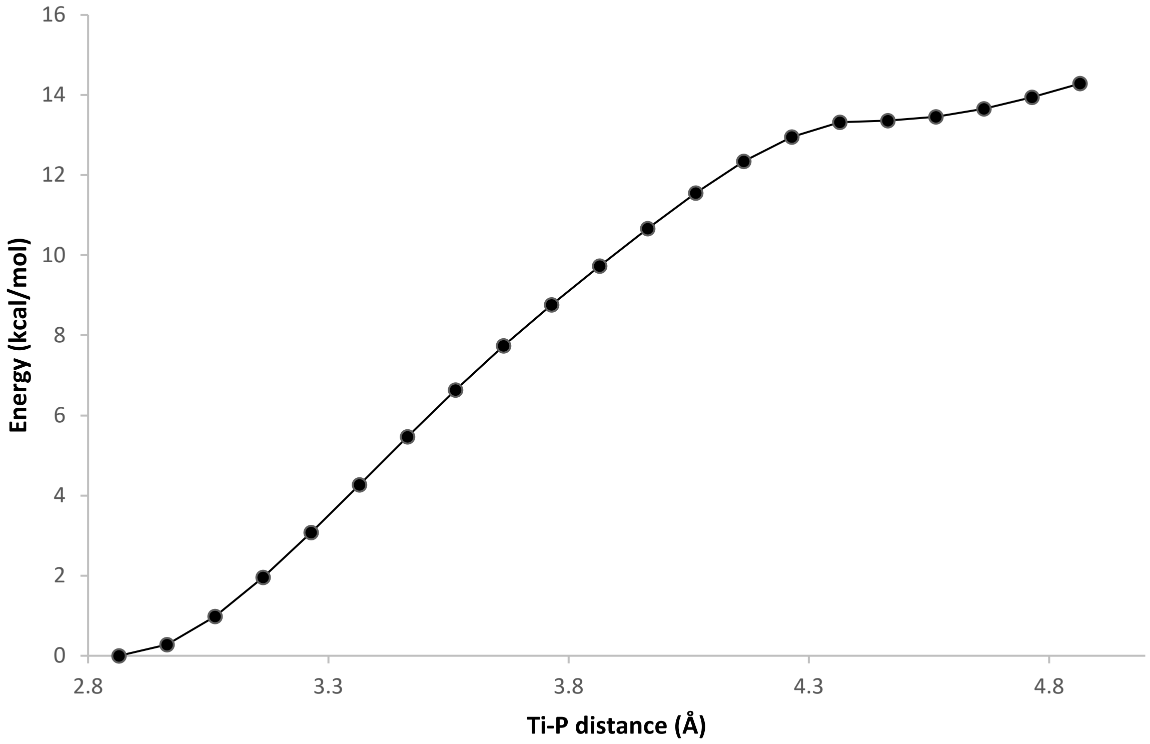

Figure S77: Potential energy surface scan of the phosphine dissociation from **4**$^+$ by varying the Ti-P distance.

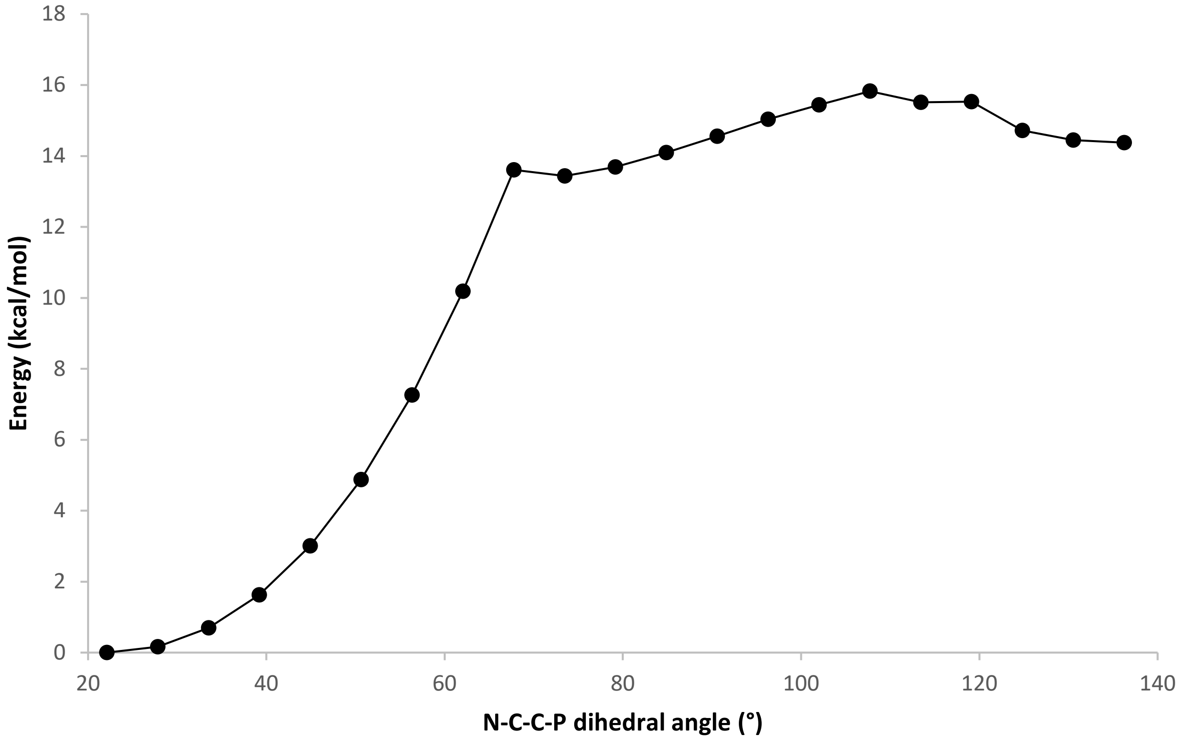

Figure S78: Potential energy surface scan of the phosphine dissociation from **4**$^+$ by rotation around the $C_{(napy)}$-$C_{(methylene)}$ bond.

2.21 Potential Energy Scan of Aldehyde Association to [4-P]$^+$:

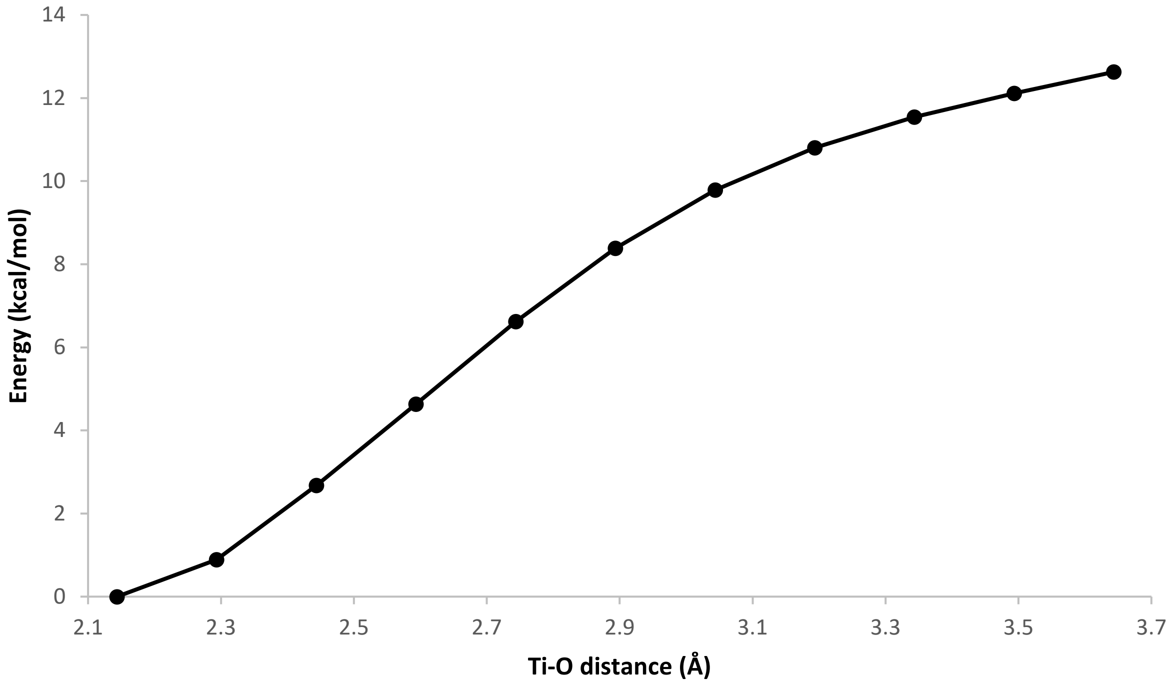

Figure S79: Potential energy surface scan of the association of diphenylacetaldehyde to [**4-P**]$^+$ by varying the Ti-O distance.

2.22 Potential Energy Scan of the Phosphine Arm Twisting in [Int-A]$^+$:

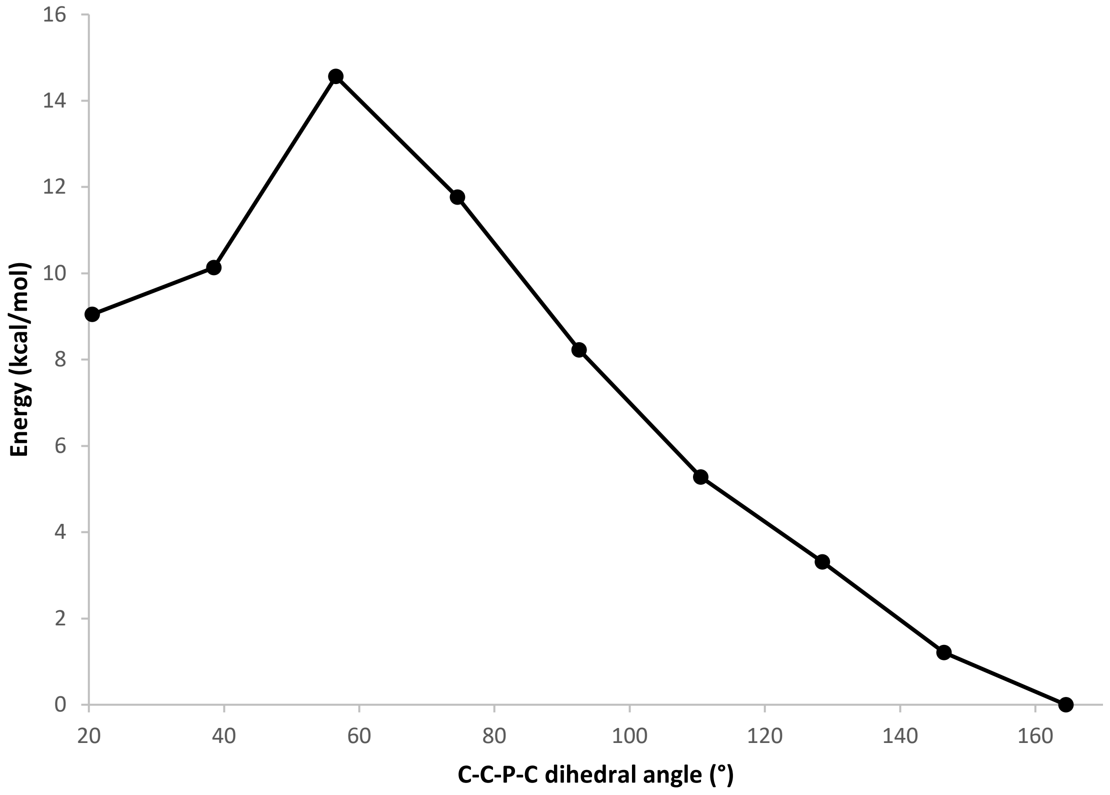

Figure S80: Potential energy surface scan of the phosphine twisting in [**Int-A**]$^+$ by rotation of the P-$C_{(methylene)}$ bond. A barrier of approx. 15 kcal/mol was found, showing that the twisting of the phosphine arm is accessible at room temperature.

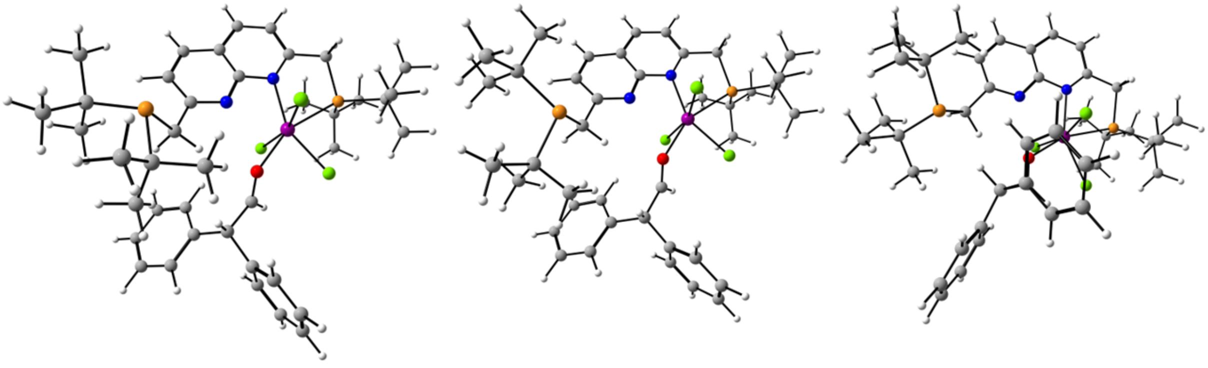

Figure S81: The three structures highest in energy of the potential energy surface scan of the phosphine twisting in **Int**-**A** by rotation of the P-$C_{(methylene)}$ bond with their respective C-C-P-C dihedral angle values.

2.23 Potential Energy Scan of the P-C bond from [Int-B]$^+$ to [Int-C]$^+$:

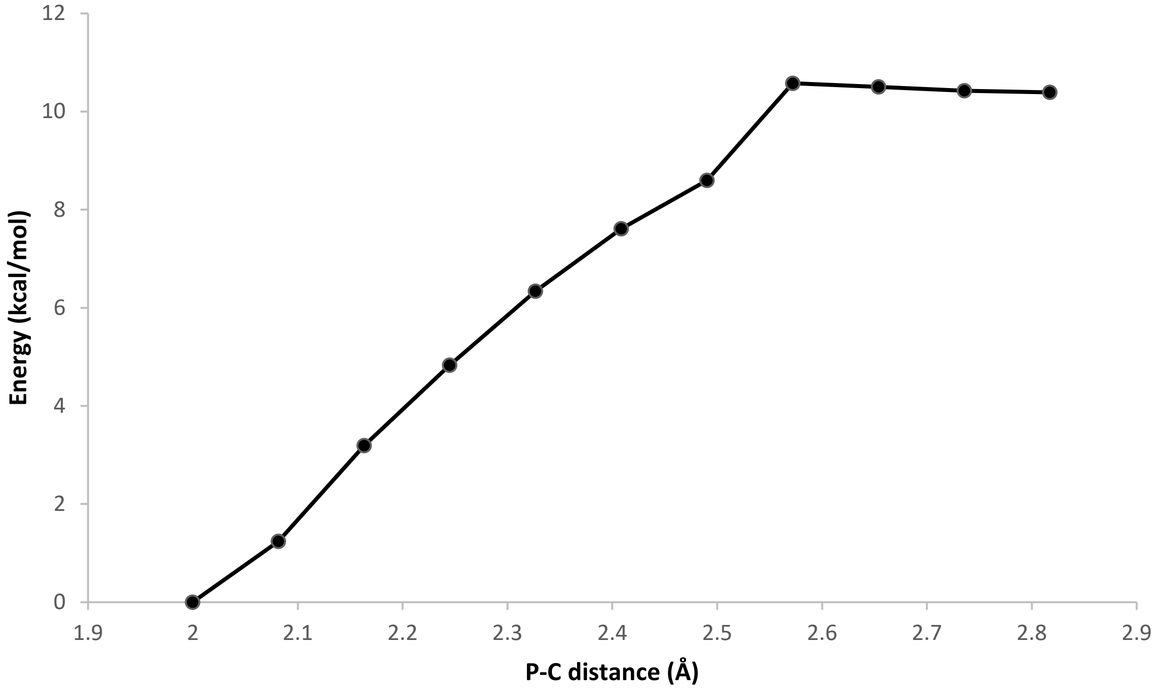

Figure S82: Potential energy surface scan of the P-C bond from [**Int**-**B**]$^+$ to [**Int**-**C**]$^+$.

2.24 Potential Energy Scan of the Phosphine Dissociation from [Int-C]$^+$:

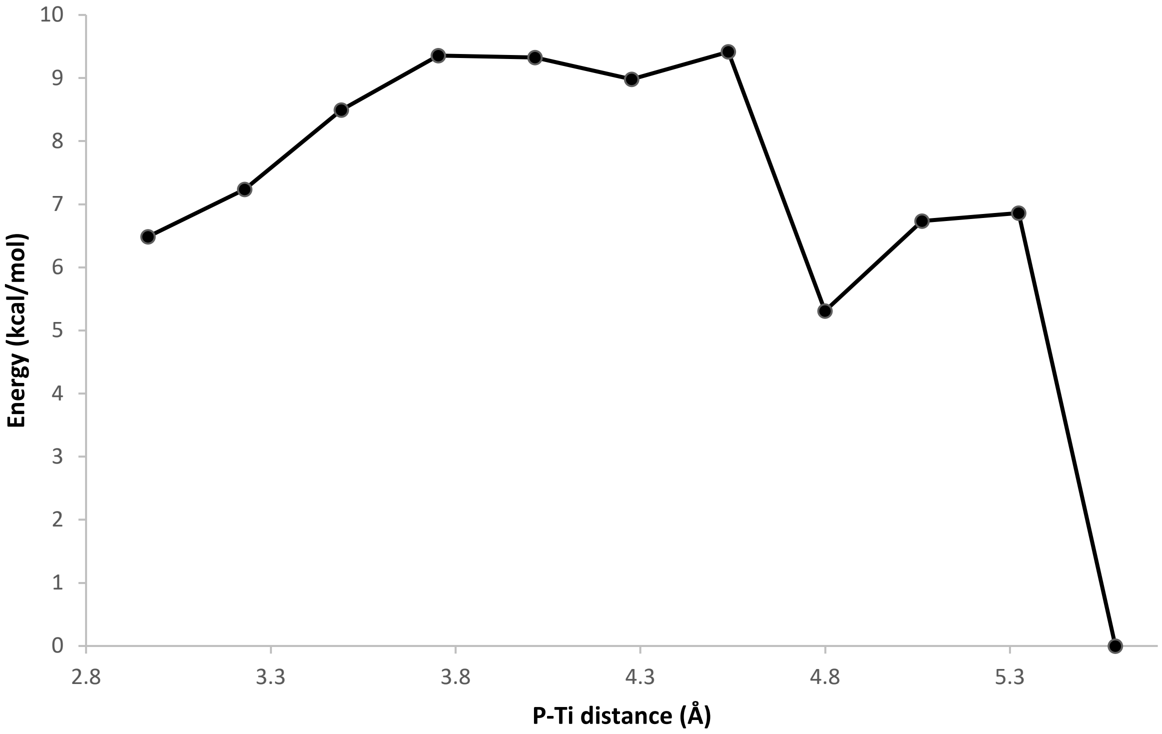

Figure S83: Potential energy surface scan of the phosphine dissociation from [**Int**-**C**]$^+$ by increasing the Ti-P bond length.

3. Crystallographic Information

3.1 X-ray crystal structure determination of 1:

$[C_{26}H_{44}Cl_3N_2P_2Ti](C_4H_8Cl_5OTi) \cdot 2CH_2Cl_2$, Fw = 1067.92, green needle, 0.49 × 0.09 × 0.04 mm^3, monoclinic, $P2_1/c$ (no. 14), a = 9.6830(8), b = 38.907(4), c = 13.2918(11) Å, β = 106.154(4) °, V = 4809.8(8) $Å^3$, Z = 4, D_x = 1.475 g/cm^3, μ = 1.09 mm^{-1}. The diffraction experiment was performed on a Bruker Kappa ApexII diffractometer with sealed tube and Triumph monochromator (λ = 0.71073 Å) at a temperature of 150(2) K up to a resolution of $(\sin \theta/\lambda)_{max}$ = 0.55 $Å^{-1}$. The diffraction pattern is characterized by the presence of diffuse streaks in the *hkl*=(0,1,0) direction. The Eval15 software[25] was used for the intensity integration. The prediction of the reflection profiles involves a *mica*-type simulation of the diffuse contribution and additionally a very large anisotropic mosaicity[26] about *hkl*=(0,0,1). A multi-scan absorption correction and scaling was performed with SADABS[27] (correction range 0.52-0.74). A total of 33576 reflections was measured, 5689 reflections were unique (R_{int} = 0.189), 3104 reflections were observed [$I>2\sigma(I)$]. Because of the long *b*-axis length and the large reflection profiles, many reflections were overlapping and the overall completeness is consequently only 84.7%. The structure was solved with Patterson superposition methods using SHELXT.[28] Structure refinement was performed with SHELXL-2018[29] on F^2 of all reflections. Non-hydrogen atoms were refined freely with anisotropic displacement parameters. The coordinated THF and the co-crystallized dichloromethane molecules were refined with disorder models. Hydrogen atoms were introduced in calculated positions and refined with a riding model. 553 Parameters were refined with 1097 restraints (geometries of the disordered groups and of the *t*-butyl substituents, displacement parameters of all atoms). R1/wR2 [$I > 2\sigma(I)$]: 0.1107 / 0.2692. R1/wR2 [all refl.]: 0.1900 / 0.3155. S = 1.047. Residual electron density between -0.74 and 1.59 e/$Å^3$. Geometry calculations and checking for higher symmetry was performed with the PLATON program.[30]

3.2 X-ray crystal structure determination of 2:

$C_{26}H_{44}Cl_4N_2P_2Ti \cdot ½C_6H_6 \cdot C_5H_{12}$, Fw = 747.47, orange needle, 0.41 × 0.09 × 0.04 mm^3, monoclinic, $P2_1/c$ (no. 14), a = 16.8812(14), b = 16.6100(11), c = 15.6315(9) Å, β = 111.688(3) °, V = 4072.8(5) $Å^3$, Z = 4, D_x = 1.219 g/cm^3, μ = 0.58 mm^{-1}. The diffraction experiment was performed on a Bruker Kappa ApexII diffractometer with sealed tube and Triumph monochromator (λ = 0.71073 Å) at a temperature of 150(2) K up to a resolution of $(\sin \theta/\lambda)_{max}$ = 0.61 $Å^{-1}$. The Eval15 software[25] was used for the intensity integration of this weakly diffracting crystal. A numerical absorption correction and scaling was performed with SADABS[27] (correction range 0.69-1.00). A total of 56073 reflections was measured, 7587 reflections were unique (R_{int} = 0.149), 4198 reflections were observed [$I>2\sigma(I)$]. The structure was solved with Patterson superposition methods using SHELXT.[28] Structure refinement was performed with SHELXL-2018[29] on F^2 of all reflections. Non-hydrogen atoms were refined freely with anisotropic displacement parameters. The co-crystallized *n*-pentane molecule was refined with a disorder model. Hydrogen atoms were introduced in calculated positions and refined with a riding model. 446 Parameters were refined with 416 restraints (geometries and displacement parameters of disordered *n*-pentane). R1/wR2 [$I > 2\sigma(I)$]: 0.0715 / 0.1625. R1/wR2 [all refl.]: 0.1489 / 0.1987. S = 1.020. Residual electron density between -0.49 and 0.89 e/$Å^3$. Geometry calculations and checking for higher symmetry was performed with the PLATON program.[30]

3.3 X-ray crystal structure determination of 3:

$C_{26}H_{44}Au_2Cl_6N_2P_2Ti \cdot C_5H_{12}$, Fw = 1173.25, pale yellow needle, 0.33 × 0.09 × 0.04 mm^3, orthorhombic, Pbca (no. 61), a = 15.1125(3), b = 20.1870(5), c = 27.1948(6) Å, V = 8296.5(3) $Å^3$, Z = 8, D_x = 1.879 g/cm^3, μ = 7.73 mm^{-1}. The diffraction experiment was performed on a Bruker Kappa ApexII diffractometer with sealed tube and Triumph monochromator (λ = 0.71073 Å) at a temperature of 150(2) K up to a resolution of $(\sin \theta/\lambda)_{max}$ = 0.65 $Å^{-1}$. The Eval15 software[22] was used for the intensity integration. A numerical absorption correction and scaling was performed with SADABS[24] (correction range 0.43-0.74). A total of 104625 reflections was measured, 9529 reflections were unique (R_{int} = 0.078), 6784 reflections were observed [$I>2\sigma(I)$]. The structure was solved with Patterson superposition methods using SHELXT.[28] Structure refinement was performed with SHELXL-2018[29] on F^2 of all reflections. Non-hydrogen atoms were refined freely with anisotropic displacement parameters. Hydrogen atoms were introduced in calculated positions and refined with a riding model. 411 Parameters were refined with no restraints. R1/wR2 [$I > 2\sigma(I)$]: 0.0373 / 0.0739. R1/wR2 [all refl.]: 0.0669 / 0.0830. S = 1.030. Residual electron density between -1.05 and 2.38 e/$Å^3$. Geometry calculations and checking for higher symmetry was performed with the PLATON program.[30]

3.4 X-ray crystal structure determination of 4:

$[C_{26}H_{44}Cl_3N_2P_2Ti](C_{32}H_{12}BF_{24})$, Fw = 1464.04, dark red block, 0.42 × 0.31 × 0.08 mm^3, monoclinic, $P2_1/c$ (no. 14), a = 18.2372(13), b = 18.2261(10), c = 19.3593(8) Å, β = 90.255(3) °, V = 6434.8(6) $Å^3$, Z = 4, D_x = 1.511 g/cm^3, μ = 0.42 mm^{-1}. The diffraction experiment was performed on a Bruker Kappa ApexII diffractometer with sealed tube and Triumph monochromator (λ = 0.71073 Å) at a temperature of 150(2) K up to a resolution of $(\sin \theta/\lambda)_{max}$ = 0.60 $Å^{-1}$. The Eval15 software[25] was used for the intensity integration. A multi-scan absorption correction and scaling was performed with SADABS[24] (correction range 0.61-0.75). A total of 41765 reflections was measured, 11528 reflections were unique (R_{int} = 0.059), 7453 reflections were observed [$I>2\sigma(I)$]. The structure was solved with Patterson superposition methods using SHELXT.[28] Structure refinement was performed with SHELXL-2018[29] on F^2 of all reflections. Non-hydrogen atoms were refined freely with anisotropic displacement parameters. Hydrogen atoms were introduced in calculated positions and refined with a riding model. 446 Parameters were refined with no restraints. R1/wR2 [$I > 2\sigma(I)$]: 0.0589 / 0.1456. R1/wR2 [all refl.]: 0.1030 / 0.1699. S = 1.029. Residual electron density between -0.75 and 0.68 e/$Å^3$. Geometry calculations and checking for higher symmetry was performed with the PLATON program.[30]

3.5 X-ray crystal structure determination of 5:

$[C_{40}H_{56}Cl_3N_2OP_2Ti](C_{32}H_{12}BF_{24}) \cdot 2C_7H_8$, Fw = 1844.55, orange plate, 0.37 × 0.16 × 0.04 mm^3, triclinic, $P\overline{1}$ (no. 2), a = 12.8524(3), b = 18.0304(7), c = 21.3060(6) Å, α = 102.735(1), β = 104.569(1), γ = 105.177(1) °, V = 4389.9(2) $Å^3$, Z = 2, D_x = 1.395 g/cm^3, μ = 0.32 mm^{-1}. The diffraction experiment was performed on a Bruker Kappa ApexII diffractometer with sealed tube and Triumph monochromator (λ = 0.71073 Å) at a temperature of 150(2) K up to a resolution of $(\sin \theta/\lambda)_{max}$ = 0.65 $Å^{-1}$. The Eval15 software[25] was used for the intensity integration. A multi-scan absorption correction and scaling was performed with SADABS[27] (correction range 0.69-0.75). A total of 123576 reflections was measured, 20170 reflections were unique (R_{int} = 0.114), 11260 reflections were observed [$I>2\sigma(I)$]. The structure was solved with Patterson superposition methods using SHELXT.[28] Structure refinement was performed with SHELXL-2018[29] on F^2 of all reflections. Non-hydrogen atoms were refined freely with anisotropic displacement parameters. Four of the trifluoromethyl groups were refined with a disorder model. Hydrogen atoms of the Ti complex were located in difference Fourier maps. All other hydrogen atoms were introduced in calculated positions. The hydrogen atoms were refined with a riding model. 1207 Parameters were refined with 2818 restraints (geometries and displacement parameters of the trifluoromethyl groups). R1/wR2 [$I > 2\sigma(I)$]: 0.0566 / 0.1154. R1/wR2 [all refl.]: 0.1277 / 0.1418. S = 1.014. Residual electron density between -0.38 and 0.50 e/$Å^3$. Geometry calculations and checking for higher symmetry was performed with the PLATON program.[30]

4. References

1 E. Kounalis, M. Lutz and D. L. J. Broere, *Organometallics*, 2020, **39**, 585–592.
2 J. Echeverría and S. Alvarez, *Chem. Sci.*, 2023, **14**, 11647–11688.
3 B. Cordero, V. Gómez, A. E. Platero-Prats, M. Revés, J. Echeverría, E. Cremades, F. Barragán and S. Alvarez, *Dalton Trans.*, 2008, 2832–2838.
4 S. Alvarez, *Dalton Trans.*, 2013, **42**, 8617–8636.
5 M. J. Frisch, G. W. Trucks, H. B. Schlegel, G. E. Scuseria, M. A. Robb, J. R. Cheeseman, G. Scalmani, V. Barone, G. A. Petersson, H. Nakatsuji, X. Li, M. Caricato, A. Marenich, J. Bloino, B. G. Janesko, R. Gomperts, B. Mennucci, H. P. Hratchian, J. V. Ortiz, A. F. Izmaylov, J. L. Sonnenberg, D. Williams-Young, F. Ding, F. Lipparini, F. Egidi, J. Goings, B. Peng, A. Petrone, T. Henderson, D. Ranasinghe, V. G. Zakrzewski, J. Gao, N. Rega, G. Zheng, W. Liang, M. Hada, M. Ehara, K. Toyota, R. Fukuda, J. Hasegawa, M. Ishida, T. Nakajima, Y. Honda, O. Kitao, H. Nakai, T. Vreven, K. Throssell, J. A. Montgomery, Jr., J. E. Peralta, F. Ogliaro, M. Bearpark, J. J. Heyd, E. Brothers, K. N. Kudin, V. N. Staroverov, T. Keith, R. Kobayashi, J. Normand, K. Raghavachari, A. Rendell, J. C. Burant, S. S. Iyengar, J. Tomasi, M. Cossi, J. M. Millam, M. Klene, C. Adamo, R. Cammi, J. W. Ochterski, R. L. Martin, K. Morokuma, O. Farkas, J. B. Foresman, and D. J. Fox, Gaussian 09, Revision D.01, Wallingford CT, (2016).
6 Frisch, M. J.; Trucks, G. W.; Schlegel, H. B.; Scuseria, G. E.; Robb, M. A.; Cheeseman, J. R.; Scalmani, G.; Barone, V.; Petersson, G. A.; Nakatsuji, H.; Li, X.; Caricato, M.; Marenich, A. V.; Bloino, J.; Janesko, B. G.; Gomperts, R.; Mennucci, B.; Hratchian, H. P.; Ortiz, J. V.; Izmaylov, A. F.; Sonnenberg, J. L.; Williams; Ding, F.; Lipparini, F.; Egidi, F.; Goings, J.; Peng, B.; Petrone, A.; Henderson, T.; Ranasinghe, D.; Zakrzewski, V. G.; Gao, J.; Rega, N.; Zheng, G.; Liang, W.; Hada, M.; Ehara, M.; Toyota, K.; Fukuda, R.; Hasegawa, J.; Ishida, M.; Nakajima, T.; Honda, Y.; Kitao, O.; Nakai, H.; Vreven, T.; Throssell, K.; Montgomery Jr., J. A.; Peralta, J. E.; Ogliaro, F.; Bearpark, M. J.; Heyd, J. J.; Brothers, E. N.; Kudin, K. N.; Staroverov, V. N.; Keith, T. A.; Kobayashi, R.; Normand, J.; Raghavachari, K.; Rendell, A. P.; Burant, J. C.; Iyengar, S. S.; Tomasi, J.; Cossi, M.; Millam, J. M.; Klene, M.; Adamo, C.; Cammi, R.; Ochterski, J. W.; Martin, R. L.; Morokuma, K.; Farkas, O.; Foresman, J. B.; Fox, D. J. Gaussian 16 Rev. C.01, Wallingford, CT, (2016).
7 A. D. Becke, *J. Chem. Phys.*, 1993, **98**, 5648–5652.
8 C. Lee, W. Yang and R. G. Parr, *Phys. Rev. B*, 1988, **37**, 785–789.
9 M. Dolg, U. Wedig, H. Stoll and H. Preuss, *J. Chem. Phys.*, 1987, **86**, 866–872.
10 R. Ditchfield, W. J. Hehre and J. A. Pople, *J. Chem. Phys.*, 2003, **54**, 724–728.
11 M. M. Francl, W. J. Pietro, W. J. Hehre, J. S. Binkley, M. S. Gordon, D. J. DeFrees and J. A. Pople, *J. Chem. Phys.*, 1982, **77**, 3654–3665.
12 M. S. Gordon, J. S. Binkley, J. A. Pople, W. J. Pietro and W. J. Hehre, *J. Am. Chem. Soc.*, 1982, **104**, 2797–2803.
13 P. C. Hariharan and J. A. Pople, *Theor. Chim. Acta*, 1973, **28**, 213–222.
14 W. J. Hehre, R. Ditchfield and J. A. Pople, *J. Chem. Phys.*, 2003, **56**, 2257–2261.
15 R. Krishnan, J. S. Binkley, R. Seeger and J. A. Pople, *J. Chem. Phys.*, 2008, **72**, 650–654.
16 A. D. McLean and G. S. Chandler, *J. Chem. Phys.*, 1980, **72**, 5639–5648.
17 S. Grimme, J. Antony, S. Ehrlich and H. Krieg, *J. Chem. Phys.*, 2010, **132**, 154104.
18 S. Grimme, S. Ehrlich and L. Goerigk, *J. Comput. Chem.*, 2011, **32**, 1456–1465.
19 J. P. Foster and F. Weinhold, *J. Am. Chem. Soc.*, 1980, **102**, 7211–7218.
20 NBO 6.0 E. D. Glendening, J. K. Badenhoop, A. E. Reed, J. E. Carpenter, J. A. Bohmann, C. M. Morales, C. R. Landis, and F. Weinhold (Theoretical Chemistry Institute, University of Wisconsin, Madison, WI, 2013).
21 A. E. Reed and F. Weinhold, *J. Chem. Phys.*, 1983, **78**, 4066–4073.
22 A. E. Reed, R. B. Weinstock and F. Weinhold, *J. Chem. Phys.*, 1985, **83**, 735–746.
23 R. S. Mulliken, *J. Chem. Phys.*, 1955, **23**, 1833–1840.
24 K. B. Wiberg, *Tetrahedron*, 1968, **24**, 1083–1096.
25 A. M. M. Schreurs, X. Xian and L. M. J. Kroon-Batenburg, *J. Appl. Cryst.*, 2010, **43**, 70–82.

26 A. J. M. Duisenberg, *Acta Cryst. A*, 1983, **39**, 211–216.
27 L. Krause, R. Herbst-Irmer, G. M. Sheldrick and D. Stalke, *J. Appl. Cryst.*, 2015, **48**, 3–10.
28 G. M. Sheldrick, *Acta Cryst. A.*, 2015, **71**, 3–8.
29 G. M. Sheldrick, *Acta Cryst. C.*, 2015, **71**, 3–8.
30 A. L. Spek, *Acta Cryst. D.* 2009, **65**, 148–155.
